# Supplementary material for: Guideline-level monitoring, biomarker levels and pharmacological treatment in migrants and native Danes with type 2 diabetes: Population-wide analyses
Source: PLOS Glob Public Health. 2023 Oct 18;3(10):e0001277. doi: 10.1371/journal.pgph.0001277 (PMC10584163; doi:10.1371/journal.pgph.0001277)
Supplement: S1 File — (HTML) [file pgph.0001277.s001.html]

S1. Register codes used to define variables.


# S1. Register codes used to define variables.

- S1. Register
  codes used to define variables.
  - Codes used to define
    outcomes.
    - Health services
      register:
    - National Patient
      Register:
    - Laboratory data codes:
  - Codes
    used to define macrovascular complication status

# S1. Register codes used to define variables.

## Codes used to define outcomes.

### Health services register:

- HbA1c monitoring (point-of-care-testing): SPECIALE codes ..7403
- Diabetic retinopathy screening in primary sector: SPECIALE codes
  190111, 190112, 193501, 193601, 196429
- Diabetic foot disease screening at podiatrist: SPEC2 code 54

### National Patient Register:

- Diabetic retinopathy screening in secondary sector (public and
  private hospitals): SKS Procedure code zz7051

### Laboratory data codes:

- HbA1c: NPU27300, NPU03835
- LDL-C: NPU01568, NPU10171
- UACR: NPU19661

## Codes used to define macrovascular complication status

#### Primary diagnoses and procedure codes from the National Patient Register:

#### Stroke:

- ICD-10 codes DI61, DI63-66 & DI69.3-DI69.4
- SKS procedure codes KAAL10 & KAAL11.

#### Ischemic heart disease:

- ICD-10 codes DI20-DI25
- SKS procedure codes KFNA-KFNG excl. KFNG20 & KFNG22.

#### Peripheral arterial disease:

- ICD-10 codes DI70, DI73.9, DI74 & DZ89.4-DZ89.7
- SKS procedure codes KPDH, KPDQ, KPAE, KPBE, KPDE, KPFE,KNGQ19,
  KNGQ29, KNFQ19 & KNFQ29.
