# Supplementary material for: Guideline-level monitoring, biomarker levels and pharmacological treatment in migrants and native Danes with type 2 diabetes: Population-wide analyses
Source: PLOS Glob Public Health. 2023 Oct 18;3(10):e0001277. doi: 10.1371/journal.pgph.0001277 (PMC10584163; doi:10.1371/journal.pgph.0001277)
Supplement: S2 File — (HTML) [file pgph.0001277.s002.html]

S2: Characteristics of study populations for analysis of T2D prevalence and pharmacological treatment.


# S2: Characteristics of study populations for analysis of T2D prevalence and pharmacological treatment.

- S2:
  Characteristics of secondary study populations
  - Population for
    analysis of T2D prevalence:
  - Population
    for analysis of pharmacological treatment: glucose-lowering
    drugs:
  - Population
    for analysis of pharmacological treatment: lipid-lowering
    drugs:
  - Population
    for analysis of pharmacological treatment: ACE inhibitors or angiotensin
    receptor blockers:

# S2: Characteristics of secondary study populations

*In compliance with the statistical disclosure policy of
Statistics Denmark, some variables (duration of residence, region of
residence) containing cells with very low counts were omitted from the
tables of populations used in analyses of pharmacological
treatment*

### Population for analysis of T2D prevalence:

| Characteristics of study population |  | Denmark | Middle East | Europe | Turkey | F. Yugoslavia | Pakistan | Sri Lanka | Somalia | Vietnam |
| --- | --- | --- | --- | --- | --- | --- | --- | --- | --- | --- |
|  |  | 3,531,328 (91.4) | 73,648 (1.9) | 161,060 (4.2) | 30,145 (0.8) | 32,678 (0.8) | 11,597 (0.3) | 6,472 (0.2) | 9,037 (0.2) | 8,563 (0.2) |
| Sex | Female (%) | 1,801,632 (51.0) | 31,784 (43.2) | 83,127 (51.6) | 14,542 (48.2) | 16,430 (50.3) | 5,445 (47.0) | 3,249 (50.2) | 4,286 (47.4) | 4,676 (54.6) |
| Age | Mean (SD) | 54.2 (16.8) | 45.9 (13.2) | 46.8 (16.4) | 48.4 (12.0) | 48.4 (14.3) | 48.4 (13.9) | 49.1 (12.3) | 42.6 (12.4) | 48.8 (12.8) |
| Employment | Employed | 2,163,769 (61.3) | 36,956 (50.2) | 119,297 (74.1) | 16,439 (54.5) | 16,656 (51.0) | 7,283 (62.8) | 3,891 (60.1) | 4,189 (46.4) | 5,631 (65.8) |
|  | Retired | 1,007,616 (28.5) | 5,450 (7.4) | 26,328 (16.3) | 3,234 (10.7) | 4,285 (13.1) | 1,759 (15.2) | 579 (8.9) | 366 (4.1) | 834 (9.7) |
|  | Unemployed | 359,943 (10.2) | 31,242 (42.4) | 15,435 (9.6) | 10,472 (34.7) | 11,737 (35.9) | 2,555 (22.0) | 2,002 (30.9) | 4,482 (49.6) | 2,098 (24.5) |
| Household income percentile | Mean (SD) | 55.2 (27.3) | 25.7 (23.3) | 44.2 (29.9) | 29.8 (21.9) | 35.4 (23.9) | 26.9 (22.7) | 38.3 (24.0) | 15.6 (14.4) | 37.0 (25.2) |
| Duration of residence (years) | < 10 | 0 (0.0) | 14,296 (19.4) | 67,456 (41.9) | 2,139 (7.1) | 2,233 (6.8) | 2,555 (22.0) | 437 (6.8) | 1,035 (11.5) | 691 (8.1) |
|  | 10 - 15 | 0 (0.0) | 4,656 (6.3) | 21,045 (13.1) | 1,271 (4.2) | 1,630 (5.0) | 619 (5.3) | 255 (3.9) | 357 (4.0) | 441 (5.2) |
|  | 15 - 20 | 0 (0.0) | 17,748 (24.1) | 13,479 (8.4) | 3,563 (11.8) | 3,552 (10.9) | 1,320 (11.4) | 603 (9.3) | 2,468 (27.3) | 802 (9.4) |
|  | > 20 | 3,531,328 (100.0) | 36,948 (50.2) | 59,080 (36.7) | 23,172 (76.9) | 25,263 (77.3) | 7,103 (61.2) | 5,177 (80.0) | 5,177 (57.3) | 6,629 (77.4) |
| Region of residence | Capital | 1,020,948 (28.9) | 33,553 (45.6) | 68,451 (42.5) | 17,891 (59.3) | 13,611 (41.7) | 10,431 (89.9) | 700 (10.8) | 3,520 (39.0) | 1,721 (20.1) |
|  | Central Denmark | 810,249 (22.9) | 14,890 (20.2) | 30,981 (19.2) | 4,069 (13.5) | 4,933 (15.1) | 224 (1.9) | 2,655 (41.0) | 2,648 (29.3) | 2,527 (29.5) |
|  | North Denmark | 383,192 (10.9) | 3,828 (5.2) | 12,398 (7.7) | 523 (1.7) | 1,915 (5.9) | 65 (0.6) | 521 (8.1) | 591 (6.5) | 1,049 (12.3) |
|  | South Denmark | 770,502 (21.8) | 12,549 (17.0) | 32,694 (20.3) | 3,058 (10.1) | 8,800 (26.9) | 474 (4.1) | 1,961 (30.3) | 1,760 (19.5) | 2,828 (33.0) |
|  | Zealand | 546,437 (15.5) | 8,828 (12.0) | 16,536 (10.3) | 4,604 (15.3) | 3,419 (10.5) | 403 (3.5) | 635 (9.8) | 518 (5.7) | 438 (5.1) |
| Type 2 diabetes | Prevalent (%) | 233,553 (6.6) | 8,110 (11.0) | 6,655 (4.1) | 4,606 (15.3) | 3,634 (11.1) | 2,783 (24.0) | 1,702 (26.3) | 989 (10.9) | 805 (9.4) |

### Population for analysis of pharmacological treatment: glucose-lowering drugs:

**Subgroup alive and resident in Denmark in the year following
the index date, with indication for treatment with glucose-lowering
drugs according to guidelines: HbA1c exceeding 48 mmol/mol.**

| Characteristics of subgroup |  | Denmark | Middle East | Europe | Turkey | F. Yugoslavia | Pakistan | Sri Lanka | Somalia | Vietnam |
| --- | --- | --- | --- | --- | --- | --- | --- | --- | --- | --- |
|  |  | 122,239 (87.2) | 4,934 (3.5) | 3,505 (2.5) | 3,097 (2.2) | 2,323 (1.7) | 1,878 (1.3) | 1,169 (0.8) | 606 (0.4) | 457 (0.3) |
| Sex | Female (%) | 50,489 (41.3) | 1,889 (38.3) | 1,541 (44.0) | 1,547 (50.0) | 1,111 (47.8) | 851 (45.3) | 485 (41.5) | 255 (42.1) | 216 (47.3) |
| Age | Mean (SD) | 66.6 (12.1) | 59.4 (10.8) | 67.3 (12.0) | 59.3 (10.8) | 62.5 (10.9) | 60.7 (11.0) | 57.3 (9.7) | 53.4 (10.9) | 63.5 (12.0) |
| Diabetes duration (years) | Mean (SD) | 9.6 (6.2) | 9.4 (6.0) | 8.8 (6.0) | 9.3 (5.9) | 9.1 (5.9) | 10.6 (6.3) | 10.7 (6.2) | 8.5 (5.8) | 8.9 (5.8) |
| Macrovascular complications | N (%) | 35,916 (29.4) | 1,441 (29.2) | 1,085 (31.0) | 887 (28.6) | 764 (32.9) | 625 (33.3) | 278 (23.8) | 80 (13.2) | 89 (19.5) |
| Diabetic kidney disease | N (%) | 7,482 (6.1) | 337 (6.8) | 190 (5.4) | 198 (6.4) | 162 (7.0) | 104 (5.5) | 88 (7.5) | 26 (4.3) | 48 (10.5) |
| Employment | Employed | 35,173 (28.8) | 908 (18.4) | 923 (26.3) | 765 (24.7) | 358 (15.4) | 643 (34.2) | 420 (35.9) | 131 (21.6) | 122 (26.7) |
|  | Retired | 70,035 (57.3) | 1,283 (26.0) | 2,102 (60.0) | 985 (31.8) | 925 (39.8) | 683 (36.4) | 218 (18.6) | 67 (11.1) | 170 (37.2) |
|  | Unemployed | 17,031 (13.9) | 2,743 (55.6) | 480 (13.7) | 1,347 (43.5) | 1,040 (44.8) | 552 (29.4) | 531 (45.4) | 408 (67.3) | 165 (36.1) |
| Household income percentile | Mean (SD) | 46.6 (25.4) | 23.1 (18.4) | 40.9 (26.8) | 24.2 (17.9) | 27.5 (18.9) | 24.9 (20.8) | 34.6 (21.7) | 15.5 (11.6) | 27.1 (20.1) |
| No hemoglobin-A1c monitoring | N (%) | 5,630 (4.6) | 252 (5.1) | 203 (5.8) | 159 (5.1) | 79 (3.4) | 109 (5.8) | 38 (3.3) | 60 (9.9) | 27 (5.9) |
| No LDL-cholesterol monitoring | N (%) | 13,842 (11.3) | 629 (12.7) | 452 (12.9) | 440 (14.2) | 243 (10.5) | 254 (13.5) | 104 (8.9) | 105 (17.3) | 52 (11.4) |
| No diabetic nephropathy screening | N (%) | 45,765 (37.4) | 2,077 (42.1) | 1,486 (42.4) | 1,289 (41.6) | 935 (40.2) | 847 (45.1) | 374 (32.0) | 328 (54.1) | 180 (39.4) |
| No diabetic retinopathy screening | N (%) | 45,961 (37.6) | 2,279 (46.2) | 1,598 (45.6) | 1,440 (46.5) | 1,111 (47.8) | 1,060 (56.4) | 313 (26.8) | 325 (53.6) | 182 (39.8) |
| No diabetic foot disease screening | N (%) | 65,794 (53.8) | 3,939 (79.8) | 2,171 (61.9) | 2,468 (79.7) | 1,828 (78.7) | 1,480 (78.8) | 759 (64.9) | 525 (86.6) | 387 (84.7) |
| Hemoglobin-A1c level | Mean (SD) | 59.9 (12.8) | 63.1 (15.0) | 60.1 (13.3) | 64.5 (15.8) | 62.3 (14.6) | 63.4 (14.4) | 61.9 (13.7) | 66.1 (17.8) | 59.4 (13.3) |
| LDL-cholesterol level | Mean (SD) | 2.1 (0.9) | 2.2 (0.9) | 2.2 (1.0) | 2.2 (0.9) | 2.2 (0.9) | 2.1 (0.9) | 2.1 (0.9) | 2.6 (0.9) | 2.1 (1.0) |
| No glucose-lowering drugs | N (%) | 8,435 (6.9) | 314 (6.4) | 350 (10.0) | 158 (5.1) | 134 (5.8) | 148 (7.9) | 49 (4.2) | 70 (11.6) | 33 (7.2) |
| No lipid-lowering drugs | N (%) | 31,032 (25.4) | 1,364 (27.6) | 1,093 (31.2) | 773 (25.0) | 479 (20.6) | 518 (27.6) | 260 (22.2) | 324 (53.5) | 111 (24.3) |
| No ACEI/ARB | N (%) | 38,345 (31.4) | 2,331 (47.2) | 1,254 (35.8) | 1,379 (44.5) | 817 (35.2) | 870 (46.3) | 545 (46.6) | 401 (66.2) | 218 (47.7) |
| No antiplatelet therapy | N (%) | 76,324 (62.4) | 3,221 (65.3) | 2,184 (62.3) | 2,032 (65.6) | 1,470 (63.3) | 1,113 (59.3) | 812 (69.5) | 532 (87.8) | 336 (73.5) |

### Population for analysis of pharmacological treatment: lipid-lowering drugs:

**Subgroup alive and resident in Denmark in the year following
the index date, with indication for treatment with lipid-lowering drugs
according to guidelines: LDL-C exceeding 2.5 mmol/L and 40 years or
older, or anyone with prevalent complications.**

| Characteristics of subgroup |  | Denmark | Middle East | Europe | Turkey | F. Yugoslavia | Pakistan | Sri Lanka | Somalia | Vietnam |
| --- | --- | --- | --- | --- | --- | --- | --- | --- | --- | --- |
|  |  | 119,183 (88.5) | 4,309 (3.2) | 3,530 (2.6) | 2,460 (1.8) | 1,942 (1.4) | 1,541 (1.1) | 821 (0.6) | 530 (0.4) | 360 (0.3) |
| Sex | Female (%) | 50,568 (42.4) | 1,591 (36.9) | 1,609 (45.6) | 1,215 (49.4) | 866 (44.6) | 611 (39.6) | 349 (42.5) | 238 (44.9) | 166 (46.1) |
| Age | Mean (SD) | 69.1 (11.7) | 60.8 (10.8) | 69.0 (11.4) | 60.4 (10.8) | 63.4 (11.0) | 62.6 (10.6) | 58.9 (9.6) | 55.6 (10.6) | 64.7 (12.1) |
| Diabetes duration (years) | Mean (SD) | 8.6 (6.1) | 8.7 (6.0) | 8.0 (5.9) | 8.6 (6.0) | 8.6 (6.0) | 9.9 (6.4) | 9.7 (6.6) | 8.0 (5.7) | 8.2 (5.9) |
| Macrovascular complications | N (%) | 67,486 (56.6) | 2,187 (50.8) | 1,893 (53.6) | 1,261 (51.3) | 1,096 (56.4) | 869 (56.4) | 381 (46.4) | 120 (22.6) | 153 (42.5) |
| Diabetic kidney disease | N (%) | 12,313 (10.3) | 444 (10.3) | 293 (8.3) | 249 (10.1) | 207 (10.7) | 137 (8.9) | 110 (13.4) | 38 (7.2) | 73 (20.3) |
| Employment | Employed | 26,658 (22.4) | 727 (16.9) | 798 (22.6) | 541 (22.0) | 273 (14.1) | 472 (30.6) | 237 (28.9) | 90 (17.0) | 83 (23.1) |
|  | Retired | 76,873 (64.5) | 1,291 (30.0) | 2,249 (63.7) | 871 (35.4) | 833 (42.9) | 652 (42.3) | 189 (23.0) | 81 (15.3) | 149 (41.4) |
|  | Unemployed | 15,652 (13.1) | 2,291 (53.2) | 483 (13.7) | 1,048 (42.6) | 836 (43.0) | 417 (27.1) | 395 (48.1) | 359 (67.7) | 128 (35.6) |
| Household income percentile | Mean (SD) | 44.7 (24.9) | 24.0 (19.2) | 41.0 (26.5) | 23.7 (17.8) | 27.5 (19.7) | 24.3 (20.6) | 32.7 (21.1) | 15.4 (11.4) | 26.9 (18.8) |
| No hemoglobin-A1c monitoring | N (%) | 8,121 (6.8) | 358 (8.3) | 285 (8.1) | 171 (7.0) | 132 (6.8) | 115 (7.5) | 43 (5.2) | 49 (9.2) | 33 (9.2) |
| No LDL-cholesterol monitoring | N (%) | 15,421 (12.9) | 657 (15.2) | 492 (13.9) | 382 (15.5) | 237 (12.2) | 236 (15.3) | 78 (9.5) | 91 (17.2) | 44 (12.2) |
| No diabetic nephropathy screening | N (%) | 52,970 (44.4) | 2,096 (48.6) | 1,767 (50.1) | 1,158 (47.1) | 887 (45.7) | 763 (49.5) | 293 (35.7) | 305 (57.5) | 143 (39.7) |
| No diabetic retinopathy screening | N (%) | 56,604 (47.5) | 2,359 (54.7) | 1,911 (54.1) | 1,347 (54.8) | 1,109 (57.1) | 957 (62.1) | 279 (34.0) | 311 (58.7) | 167 (46.4) |
| No diabetic foot disease screening | N (%) | 69,747 (58.5) | 3,562 (82.7) | 2,330 (66.0) | 2,015 (81.9) | 1,581 (81.4) | 1,260 (81.8) | 547 (66.6) | 471 (88.9) | 311 (86.4) |
| Hemoglobin-A1c level | Mean (SD) | 52.3 (13.8) | 56.2 (16.6) | 53.0 (14.2) | 58.5 (17.2) | 56.6 (16.1) | 58.4 (16.2) | 58.0 (15.5) | 59.3 (19.6) | 53.3 (13.8) |
| LDL-cholesterol level | Mean (SD) | 2.5 (1.0) | 2.6 (1.0) | 2.6 (1.1) | 2.6 (1.0) | 2.5 (1.0) | 2.5 (1.0) | 2.5 (1.0) | 3.0 (0.8) | 2.6 (1.1) |
| No glucose-lowering drugs | N (%) | 29,511 (24.8) | 873 (20.3) | 933 (26.4) | 368 (15.0) | 329 (16.9) | 298 (19.3) | 116 (14.1) | 97 (18.3) | 64 (17.8) |
| No lipid-lowering drugs | N (%) | 42,752 (35.9) | 1,538 (35.7) | 1,424 (40.3) | 786 (32.0) | 567 (29.2) | 496 (32.2) | 257 (31.3) | 304 (57.4) | 108 (30.0) |
| No ACEI/ARB | N (%) | 43,502 (36.5) | 2,135 (49.5) | 1,420 (40.2) | 1,140 (46.3) | 777 (40.0) | 763 (49.5) | 393 (47.9) | 337 (63.6) | 152 (42.2) |
| No antiplatelet therapy | N (%) | 64,613 (54.2) | 2,523 (58.6) | 2,033 (57.6) | 1,410 (57.3) | 1,095 (56.4) | 810 (52.6) | 493 (60.0) | 445 (84.0) | 219 (60.8) |

### Population for analysis of pharmacological treatment: ACE inhibitors or angiotensin receptor blockers:

**Subgroup alive and resident in Denmark in the year following
the index date, with indication for treatment with ACE inhibitors or
angiotensin receptor blockers drugs according to guidelines: Prevalent
complications.**

| Characteristics of subgroup |  | Denmark | Middle East | Europe | Turkey | F.Yugoslavia | Pakistan | Sri Lanka | Somalia | Vietnam |
| --- | --- | --- | --- | --- | --- | --- | --- | --- | --- | --- |
|  |  | 68,966 (89.1) | 2,370 (3.1) | 1,906 (2.5) | 1,351 (1.7) | 1,155 (1.5) | 884 (1.1) | 439 (0.6) | 145 (0.2) | 198 (0.3) |
| Sex | Female (%) | 24,893 (36.1) | 757 (31.9) | 726 (38.1) | 602 (44.6) | 497 (43.0) | 297 (33.6) | 161 (36.7) | 50 (34.5) | 87 (43.9) |
| Age | Mean (SD) | 70.9 (10.5) | 63.4 (10.4) | 71.3 (9.9) | 63.2 (10.5) | 65.2 (10.4) | 65.6 (9.4) | 61.1 (9.5) | 57.9 (10.9) | 67.7 (11.7) |
| Diabetes duration (years) | Mean (SD) | 9.5 (6.3) | 10.2 (6.2) | 9.1 (6.1) | 10.1 (6.2) | 9.6 (6.1) | 11.6 (6.4) | 11.8 (6.6) | 10.5 (6.4) | 9.3 (6.0) |
| Macrovascular complications | N (%) | 62,388 (90.5) | 2,122 (89.5) | 1,762 (92.4) | 1,223 (90.5) | 1,042 (90.2) | 817 (92.4) | 373 (85.0) | 117 (80.7) | 151 (76.3) |
| Diabetic kidney disease | N (%) | 11,180 (16.2) | 432 (18.2) | 256 (13.4) | 241 (17.8) | 198 (17.1) | 127 (14.4) | 105 (23.9) | 37 (25.5) | 69 (34.8) |
| Employment | Employed | 10,737 (15.6) | 263 (11.1) | 268 (14.1) | 197 (14.6) | 86 (7.4) | 195 (22.1) | 85 (19.4) | 12 (8.3) | 26 (13.1) |
|  | Retired | 49,654 (72.0) | 924 (39.0) | 1,393 (73.1) | 620 (45.9) | 587 (50.8) | 471 (53.3) | 132 (30.1) | 31 (21.4) | 108 (54.5) |
|  | Unemployed | 8,575 (12.4) | 1,183 (49.9) | 245 (12.9) | 534 (39.5) | 482 (41.7) | 218 (24.7) | 222 (50.6) | 102 (70.3) | 64 (32.3) |
| Household income percentile | Mean (SD) | 42.5 (23.9) | 23.5 (17.9) | 40.1 (25.4) | 21.8 (16.2) | 25.2 (17.4) | 23.6 (19.4) | 30.8 (20.4) | 15.3 (9.1) | 24.8 (18.2) |
| No hemoglobin-A1c monitoring | N (%) | 3,246 (4.7) | 132 (5.6) | 91 (4.8) | 54 (4.0) | 61 (5.3) | 42 (4.8) | 23 (5.2) | 13 (9.0) | 16 (8.1) |
| No LDL-cholesterol monitoring | N (%) | 7,169 (10.4) | 265 (11.2) | 209 (11.0) | 168 (12.4) | 104 (9.0) | 104 (11.8) | 30 (6.8) | 24 (16.6) | 26 (13.1) |
| No diabetic nephropathy screening | N (%) | 26,464 (38.4) | 971 (41.0) | 826 (43.3) | 518 (38.3) | 480 (41.6) | 387 (43.8) | 126 (28.7) | 74 (51.0) | 66 (33.3) |
| No diabetic retinopathy screening | N (%) | 29,864 (43.3) | 1,193 (50.3) | 959 (50.3) | 677 (50.1) | 637 (55.2) | 499 (56.4) | 123 (28.0) | 78 (53.8) | 92 (46.5) |
| No diabetic foot disease screening | N (%) | 36,663 (53.2) | 1,854 (78.2) | 1,140 (59.8) | 1,042 (77.1) | 922 (79.8) | 689 (77.9) | 264 (60.1) | 125 (86.2) | 166 (83.8) |
| Hemoglobin-A1c level | Mean (SD) | 52.9 (13.6) | 57.6 (16.5) | 54.0 (14.1) | 59.8 (17.1) | 57.9 (16.4) | 59.0 (15.4) | 58.7 (15.0) | 60.0 (18.0) | 53.6 (14.2) |
| LDL-cholesterol level | Mean (SD) | 2.0 (0.9) | 2.0 (0.9) | 2.0 (0.9) | 2.0 (0.9) | 2.1 (0.9) | 2.0 (0.9) | 1.9 (0.9) | 2.4 (0.9) | 1.9 (0.9) |
| No glucose-lowering drugs | N (%) | 13,725 (19.9) | 296 (12.5) | 398 (20.9) | 136 (10.1) | 131 (11.3) | 128 (14.5) | 31 (7.1) | 19 (13.1) | 23 (11.6) |
| No lipid-lowering drugs | N (%) | 13,073 (19.0) | 454 (19.2) | 416 (21.8) | 230 (17.0) | 169 (14.6) | 140 (15.8) | 68 (15.5) | 56 (38.6) | 29 (14.6) |
| No ACEI/ARB | N (%) | 19,608 (28.4) | 843 (35.6) | 553 (29.0) | 477 (35.3) | 351 (30.4) | 326 (36.9) | 160 (36.4) | 62 (42.8) | 49 (24.7) |
| No antiplatelet therapy | N (%) | 24,010 (34.8) | 853 (36.0) | 684 (35.9) | 449 (33.2) | 433 (37.5) | 253 (28.6) | 145 (33.0) | 77 (53.1) | 77 (38.9) |
