# Supplementary material for: Guideline-level monitoring, biomarker levels and pharmacological treatment in migrants and native Danes with type 2 diabetes: Population-wide analyses
Source: PLOS Glob Public Health. 2023 Oct 18;3(10):e0001277. doi: 10.1371/journal.pgph.0001277 (PMC10584163; doi:10.1371/journal.pgph.0001277)
Supplement: S3 File — (HTML) [file pgph.0001277.s003.html]

S3: Supplementary analyses stratified by sex.


# S3: Supplementary analyses stratified by sex.

- S3: Supplementary analyses stratified by sex.
  - Prevalence: Analysis of prevalent type 2 diabetes
  - Monitoring: Analysis of HbA1c monitoring
  - Monitoring: Analysis of LDL-C monitoring
  - Monitoring: Analysis of screening for diabetic nephropathy
  - Monitoring: Analysis of screening for diabetic retinopathy
  - Monitoring: Analysis of screening for diabetic foot disease
  - Biomarker levels: Analysis of HbA1c levels
  - Biomarker levels: Analysis of LDL-C levels
  - Pharmacological treatment: Analysis of glucose-lowering drugs
  - Pharmacological treatment: Analysis of lipid-lowering drugs
  - Pharmacological treatment: Analysis of ACEI/ARB
  - Pharmacological treatment: Analysis of antiplatelet therapy

# S3: Supplementary analyses stratified by sex.

### Prevalence: Analysis of prevalent type 2 diabetes

| Prevalence: Analysis of prevalent type 2 diabetes | Sex | Model | Estimate | Lower 95% CI | Upper 95% CI |
| --- | --- | --- | --- | --- | --- |
| Middle East | F | 0 | 1.735 | 1.678 | 1.794 |
| Middle East | M | 0 | 1.569 | 1.528 | 1.612 |
| Middle East | F | 1 | 3.304 | 3.201 | 3.410 |
| Middle East | M | 1 | 2.474 | 2.413 | 2.537 |
| Middle East | F | 2 | 2.026 | 1.955 | 2.100 |
| Middle East | M | 2 | 1.723 | 1.675 | 1.771 |
| Europe | F | 0 | 0.650 | 0.627 | 0.673 |
| Europe | M | 0 | 0.606 | 0.587 | 0.626 |
| Europe | F | 1 | 0.935 | 0.904 | 0.968 |
| Europe | M | 1 | 0.947 | 0.918 | 0.977 |
| Europe | F | 2 | 0.973 | 0.940 | 1.008 |
| Europe | M | 2 | 0.961 | 0.929 | 0.993 |
| Turkey | F | 0 | 2.804 | 2.700 | 2.911 |
| Turkey | M | 0 | 1.931 | 1.858 | 2.006 |
| Turkey | F | 1 | 4.544 | 4.391 | 4.702 |
| Turkey | M | 1 | 2.882 | 2.780 | 2.988 |
| Turkey | F | 2 | 2.717 | 2.621 | 2.816 |
| Turkey | M | 2 | 2.061 | 1.987 | 2.139 |
| Former Yugoslavia | F | 0 | 1.853 | 1.772 | 1.938 |
| Former Yugoslavia | M | 0 | 1.544 | 1.479 | 1.611 |
| Former Yugoslavia | F | 1 | 2.694 | 2.584 | 2.809 |
| Former Yugoslavia | M | 1 | 2.134 | 2.050 | 2.221 |
| Former Yugoslavia | F | 2 | 1.751 | 1.678 | 1.828 |
| Former Yugoslavia | M | 2 | 1.533 | 1.472 | 1.597 |
| Pakistan | F | 0 | 4.005 | 3.813 | 4.206 |
| Pakistan | M | 0 | 3.308 | 3.167 | 3.456 |
| Pakistan | F | 1 | 6.218 | 5.962 | 6.486 |
| Pakistan | M | 1 | 4.502 | 4.331 | 4.679 |
| Pakistan | F | 2 | 4.125 | 3.945 | 4.312 |
| Pakistan | M | 2 | 3.550 | 3.411 | 3.695 |
| Sri Lanka | F | 0 | 3.947 | 3.702 | 4.207 |
| Sri Lanka | M | 0 | 3.983 | 3.778 | 4.200 |
| Sri Lanka | F | 1 | 6.074 | 5.716 | 6.454 |
| Sri Lanka | M | 1 | 5.266 | 5.019 | 5.525 |
| Sri Lanka | F | 2 | 4.143 | 3.898 | 4.404 |
| Sri Lanka | M | 2 | 3.957 | 3.769 | 4.153 |
| Somalia | F | 0 | 1.726 | 1.576 | 1.889 |
| Somalia | M | 0 | 1.579 | 1.461 | 1.706 |
| Somalia | F | 1 | 4.039 | 3.704 | 4.405 |
| Somalia | M | 1 | 3.498 | 3.247 | 3.769 |
| Somalia | F | 2 | 2.204 | 2.020 | 2.406 |
| Somalia | M | 2 | 2.219 | 2.057 | 2.394 |
| Vietnam | F | 0 | 1.436 | 1.305 | 1.581 |
| Vietnam | M | 0 | 1.435 | 1.310 | 1.570 |
| Vietnam | F | 1 | 2.303 | 2.108 | 2.516 |
| Vietnam | M | 1 | 1.930 | 1.773 | 2.101 |
| Vietnam | F | 2 | 1.675 | 1.532 | 1.831 |
| Vietnam | M | 2 | 1.505 | 1.382 | 1.639 |
| Native Danes (reference risk) | F | 0 | 0.057 | 0.057 | 0.058 |
| Native Danes (reference risk) | M | 0 | 0.075 | 0.075 | 0.076 |

### Monitoring: Analysis of HbA1c monitoring

| Monitoring: Analysis of HbA1c monitoring | Sex | Model | Estimate | Lower 95% CI | Upper 95% CI |
| --- | --- | --- | --- | --- | --- |
| Middle East | F | 0 | 0.975 | 0.856 | 1.110 |
| Middle East | M | 0 | 1.335 | 1.214 | 1.468 |
| Middle East | F | 1 | 0.788 | 0.691 | 0.897 |
| Middle East | M | 1 | 1.102 | 1.002 | 1.212 |
| Middle East | F | 2 | 0.794 | 0.687 | 0.917 |
| Middle East | M | 2 | 0.965 | 0.865 | 1.077 |
| Europe | F | 0 | 1.312 | 1.173 | 1.468 |
| Europe | M | 0 | 1.320 | 1.179 | 1.477 |
| Europe | F | 1 | 1.328 | 1.190 | 1.483 |
| Europe | M | 1 | 1.335 | 1.195 | 1.491 |
| Europe | F | 2 | 1.267 | 1.129 | 1.421 |
| Europe | M | 2 | 1.259 | 1.121 | 1.415 |
| Turkey | F | 0 | 0.694 | 0.581 | 0.830 |
| Turkey | M | 0 | 1.301 | 1.130 | 1.497 |
| Turkey | F | 1 | 0.583 | 0.487 | 0.698 |
| Turkey | M | 1 | 1.034 | 0.899 | 1.190 |
| Turkey | F | 2 | 0.526 | 0.438 | 0.631 |
| Turkey | M | 2 | 0.830 | 0.720 | 0.957 |
| Former Yugoslavia | F | 0 | 0.705 | 0.574 | 0.866 |
| Former Yugoslavia | M | 0 | 1.213 | 1.034 | 1.422 |
| Former Yugoslavia | F | 1 | 0.667 | 0.543 | 0.820 |
| Former Yugoslavia | M | 1 | 1.065 | 0.909 | 1.249 |
| Former Yugoslavia | F | 2 | 0.672 | 0.546 | 0.826 |
| Former Yugoslavia | M | 2 | 0.995 | 0.847 | 1.169 |
| Pakistan | F | 0 | 0.846 | 0.679 | 1.053 |
| Pakistan | M | 0 | 1.372 | 1.163 | 1.618 |
| Pakistan | F | 1 | 0.728 | 0.583 | 0.908 |
| Pakistan | M | 1 | 1.171 | 0.994 | 1.381 |
| Pakistan | F | 2 | 0.553 | 0.441 | 0.693 |
| Pakistan | M | 2 | 0.816 | 0.688 | 0.967 |
| Sri Lanka | F | 0 | 0.554 | 0.388 | 0.792 |
| Sri Lanka | M | 0 | 0.714 | 0.533 | 0.956 |
| Sri Lanka | F | 1 | 0.421 | 0.294 | 0.604 |
| Sri Lanka | M | 1 | 0.562 | 0.419 | 0.754 |
| Sri Lanka | F | 2 | 0.495 | 0.346 | 0.710 |
| Sri Lanka | M | 2 | 0.595 | 0.444 | 0.798 |
| Somalia | F | 0 | 1.125 | 0.815 | 1.553 |
| Somalia | M | 0 | 2.061 | 1.660 | 2.559 |
| Somalia | F | 1 | 0.772 | 0.555 | 1.074 |
| Somalia | M | 1 | 1.343 | 1.080 | 1.670 |
| Somalia | F | 2 | 0.821 | 0.583 | 1.156 |
| Somalia | M | 2 | 1.184 | 0.944 | 1.484 |
| Vietnam | F | 0 | 1.196 | 0.863 | 1.658 |
| Vietnam | M | 0 | 1.583 | 1.184 | 2.118 |
| Vietnam | F | 1 | 1.133 | 0.817 | 1.571 |
| Vietnam | M | 1 | 1.380 | 1.037 | 1.835 |
| Vietnam | F | 2 | 1.240 | 0.894 | 1.720 |
| Vietnam | M | 2 | 1.381 | 1.038 | 1.837 |
| Native Danes (reference risk) | F | 0 | 0.073 | 0.072 | 0.075 |
| Native Danes (reference risk) | M | 0 | 0.064 | 0.063 | 0.065 |

### Monitoring: Analysis of LDL-C monitoring

| Monitoring: Analysis of LDL-C monitoring | Sex | Model | Estimate | Lower 95% CI | Upper 95% CI |
| --- | --- | --- | --- | --- | --- |
| Middle East | F | 0 | 1.025 | 0.938 | 1.119 |
| Middle East | M | 0 | 1.280 | 1.198 | 1.368 |
| Middle East | F | 1 | 0.913 | 0.836 | 0.998 |
| Middle East | M | 1 | 1.100 | 1.029 | 1.176 |
| Middle East | F | 2 | 0.815 | 0.739 | 0.899 |
| Middle East | M | 2 | 0.948 | 0.879 | 1.022 |
| Europe | F | 0 | 1.174 | 1.081 | 1.275 |
| Europe | M | 0 | 1.172 | 1.079 | 1.273 |
| Europe | F | 1 | 1.181 | 1.089 | 1.282 |
| Europe | M | 1 | 1.190 | 1.097 | 1.291 |
| Europe | F | 2 | 1.102 | 1.011 | 1.200 |
| Europe | M | 2 | 1.140 | 1.046 | 1.241 |
| Turkey | F | 0 | 1.018 | 0.919 | 1.128 |
| Turkey | M | 0 | 1.340 | 1.219 | 1.473 |
| Turkey | F | 1 | 0.950 | 0.857 | 1.053 |
| Turkey | M | 1 | 1.121 | 1.020 | 1.232 |
| Turkey | F | 2 | 0.797 | 0.717 | 0.886 |
| Turkey | M | 2 | 0.877 | 0.797 | 0.965 |
| Former Yugoslavia | F | 0 | 0.781 | 0.681 | 0.896 |
| Former Yugoslavia | M | 0 | 1.110 | 0.989 | 1.245 |
| Former Yugoslavia | F | 1 | 0.786 | 0.685 | 0.902 |
| Former Yugoslavia | M | 1 | 1.007 | 0.898 | 1.130 |
| Former Yugoslavia | F | 2 | 0.745 | 0.649 | 0.855 |
| Former Yugoslavia | M | 2 | 0.920 | 0.820 | 1.033 |
| Pakistan | F | 0 | 1.031 | 0.899 | 1.184 |
| Pakistan | M | 0 | 1.366 | 1.220 | 1.530 |
| Pakistan | F | 1 | 0.987 | 0.860 | 1.133 |
| Pakistan | M | 1 | 1.219 | 1.089 | 1.365 |
| Pakistan | F | 2 | 0.713 | 0.619 | 0.821 |
| Pakistan | M | 2 | 0.840 | 0.747 | 0.944 |
| Sri Lanka | F | 0 | 0.634 | 0.501 | 0.803 |
| Sri Lanka | M | 0 | 0.767 | 0.630 | 0.934 |
| Sri Lanka | F | 1 | 0.536 | 0.424 | 0.679 |
| Sri Lanka | M | 1 | 0.634 | 0.520 | 0.771 |
| Sri Lanka | F | 2 | 0.578 | 0.457 | 0.731 |
| Sri Lanka | M | 2 | 0.666 | 0.548 | 0.810 |
| Somalia | F | 0 | 1.028 | 0.811 | 1.303 |
| Somalia | M | 0 | 1.721 | 1.464 | 2.022 |
| Somalia | F | 1 | 0.794 | 0.627 | 1.007 |
| Somalia | M | 1 | 1.211 | 1.029 | 1.425 |
| Somalia | F | 2 | 0.746 | 0.586 | 0.948 |
| Somalia | M | 2 | 1.058 | 0.896 | 1.248 |
| Vietnam | F | 0 | 0.840 | 0.636 | 1.109 |
| Vietnam | M | 0 | 1.071 | 0.835 | 1.374 |
| Vietnam | F | 1 | 0.815 | 0.619 | 1.074 |
| Vietnam | M | 1 | 0.946 | 0.739 | 1.212 |
| Vietnam | F | 2 | 0.840 | 0.639 | 1.104 |
| Vietnam | M | 2 | 0.963 | 0.754 | 1.230 |
| Native Danes (reference risk) | F | 0 | 0.139 | 0.137 | 0.141 |
| Native Danes (reference risk) | M | 0 | 0.125 | 0.123 | 0.127 |

### Monitoring: Analysis of screening for diabetic nephropathy

| Monitoring: Analysis of screening for diabetic nephropathy | Sex | Model | Estimate | Lower 95% CI | Upper 95% CI |
| --- | --- | --- | --- | --- | --- |
| Middle East | F | 0 | 1.019 | 0.982 | 1.057 |
| Middle East | M | 0 | 1.200 | 1.164 | 1.237 |
| Middle East | F | 1 | 1.024 | 0.987 | 1.061 |
| Middle East | M | 1 | 1.169 | 1.135 | 1.205 |
| Middle East | F | 2 | 0.975 | 0.936 | 1.016 |
| Middle East | M | 2 | 1.050 | 1.015 | 1.086 |
| Europe | F | 0 | 1.133 | 1.096 | 1.171 |
| Europe | M | 0 | 1.133 | 1.091 | 1.176 |
| Europe | F | 1 | 1.118 | 1.083 | 1.155 |
| Europe | M | 1 | 1.120 | 1.079 | 1.162 |
| Europe | F | 2 | 1.076 | 1.041 | 1.113 |
| Europe | M | 2 | 1.089 | 1.048 | 1.131 |
| Turkey | F | 0 | 1.008 | 0.966 | 1.052 |
| Turkey | M | 0 | 1.116 | 1.065 | 1.170 |
| Turkey | F | 1 | 1.027 | 0.985 | 1.072 |
| Turkey | M | 1 | 1.078 | 1.029 | 1.129 |
| Turkey | F | 2 | 0.942 | 0.902 | 0.983 |
| Turkey | M | 2 | 0.942 | 0.898 | 0.987 |
| Former Yugoslavia | F | 0 | 0.976 | 0.928 | 1.028 |
| Former Yugoslavia | M | 0 | 1.085 | 1.029 | 1.144 |
| Former Yugoslavia | F | 1 | 1.001 | 0.952 | 1.053 |
| Former Yugoslavia | M | 1 | 1.072 | 1.018 | 1.129 |
| Former Yugoslavia | F | 2 | 0.962 | 0.915 | 1.012 |
| Former Yugoslavia | M | 2 | 0.980 | 0.931 | 1.032 |
| Pakistan | F | 0 | 1.050 | 0.993 | 1.111 |
| Pakistan | M | 0 | 1.205 | 1.143 | 1.270 |
| Pakistan | F | 1 | 1.078 | 1.020 | 1.139 |
| Pakistan | M | 1 | 1.205 | 1.144 | 1.269 |
| Pakistan | F | 2 | 0.906 | 0.856 | 0.959 |
| Pakistan | M | 2 | 0.969 | 0.918 | 1.022 |
| Sri Lanka | F | 0 | 0.758 | 0.688 | 0.835 |
| Sri Lanka | M | 0 | 0.821 | 0.749 | 0.900 |
| Sri Lanka | F | 1 | 0.758 | 0.689 | 0.833 |
| Sri Lanka | M | 1 | 0.827 | 0.755 | 0.905 |
| Sri Lanka | F | 2 | 0.850 | 0.774 | 0.932 |
| Sri Lanka | M | 2 | 0.921 | 0.843 | 1.006 |
| Somalia | F | 0 | 1.186 | 1.090 | 1.290 |
| Somalia | M | 0 | 1.393 | 1.292 | 1.501 |
| Somalia | F | 1 | 1.131 | 1.038 | 1.231 |
| Somalia | M | 1 | 1.282 | 1.189 | 1.382 |
| Somalia | F | 2 | 1.136 | 1.039 | 1.242 |
| Somalia | M | 2 | 1.184 | 1.095 | 1.281 |
| Vietnam | F | 0 | 0.829 | 0.732 | 0.939 |
| Vietnam | M | 0 | 1.057 | 0.943 | 1.185 |
| Vietnam | F | 1 | 0.861 | 0.763 | 0.972 |
| Vietnam | M | 1 | 1.060 | 0.949 | 1.184 |
| Vietnam | F | 2 | 0.912 | 0.809 | 1.029 |
| Vietnam | M | 2 | 1.083 | 0.972 | 1.207 |
| Native Danes (reference risk) | F | 0 | 0.481 | 0.478 | 0.484 |
| Native Danes (reference risk) | M | 0 | 0.400 | 0.398 | 0.403 |

### Monitoring: Analysis of screening for diabetic retinopathy

| Monitoring: Analysis of screening for diabetic retinopathy | Sex | Model | Estimate | Lower 95% CI | Upper 95% CI |
| --- | --- | --- | --- | --- | --- |
| Middle East | F | 0 | 1.109 | 1.068 | 1.152 |
| Middle East | M | 0 | 1.210 | 1.177 | 1.244 |
| Middle East | F | 1 | 1.053 | 1.015 | 1.094 |
| Middle East | M | 1 | 1.114 | 1.084 | 1.146 |
| Middle East | F | 2 | 0.927 | 0.889 | 0.966 |
| Middle East | M | 2 | 0.963 | 0.933 | 0.993 |
| Europe | F | 0 | 1.178 | 1.136 | 1.221 |
| Europe | M | 0 | 1.192 | 1.153 | 1.232 |
| Europe | F | 1 | 1.161 | 1.122 | 1.203 |
| Europe | M | 1 | 1.175 | 1.138 | 1.213 |
| Europe | F | 2 | 1.114 | 1.074 | 1.156 |
| Europe | M | 2 | 1.145 | 1.107 | 1.185 |
| Turkey | F | 0 | 1.114 | 1.066 | 1.163 |
| Turkey | M | 0 | 1.182 | 1.135 | 1.232 |
| Turkey | F | 1 | 1.097 | 1.050 | 1.146 |
| Turkey | M | 1 | 1.071 | 1.028 | 1.116 |
| Turkey | F | 2 | 0.923 | 0.883 | 0.965 |
| Turkey | M | 2 | 0.887 | 0.852 | 0.925 |
| Former Yugoslavia | F | 0 | 1.147 | 1.093 | 1.205 |
| Former Yugoslavia | M | 0 | 1.260 | 1.208 | 1.315 |
| Former Yugoslavia | F | 1 | 1.153 | 1.098 | 1.210 |
| Former Yugoslavia | M | 1 | 1.189 | 1.140 | 1.239 |
| Former Yugoslavia | F | 2 | 1.061 | 1.011 | 1.113 |
| Former Yugoslavia | M | 2 | 1.082 | 1.038 | 1.127 |
| Pakistan | F | 0 | 1.321 | 1.258 | 1.388 |
| Pakistan | M | 0 | 1.399 | 1.343 | 1.459 |
| Pakistan | F | 1 | 1.351 | 1.286 | 1.419 |
| Pakistan | M | 1 | 1.357 | 1.303 | 1.414 |
| Pakistan | F | 2 | 1.046 | 0.994 | 1.101 |
| Pakistan | M | 2 | 1.036 | 0.993 | 1.081 |
| Sri Lanka | F | 0 | 0.651 | 0.579 | 0.733 |
| Sri Lanka | M | 0 | 0.776 | 0.709 | 0.849 |
| Sri Lanka | F | 1 | 0.605 | 0.539 | 0.678 |
| Sri Lanka | M | 1 | 0.734 | 0.672 | 0.802 |
| Sri Lanka | F | 2 | 0.630 | 0.562 | 0.706 |
| Sri Lanka | M | 2 | 0.764 | 0.700 | 0.833 |
| Somalia | F | 0 | 1.240 | 1.133 | 1.358 |
| Somalia | M | 0 | 1.335 | 1.242 | 1.434 |
| Somalia | F | 1 | 1.068 | 0.975 | 1.170 |
| Somalia | M | 1 | 1.150 | 1.067 | 1.239 |
| Somalia | F | 2 | 0.968 | 0.882 | 1.062 |
| Somalia | M | 2 | 0.992 | 0.919 | 1.069 |
| Vietnam | F | 0 | 0.939 | 0.830 | 1.062 |
| Vietnam | M | 0 | 1.046 | 0.940 | 1.165 |
| Vietnam | F | 1 | 0.926 | 0.821 | 1.044 |
| Vietnam | M | 1 | 0.975 | 0.878 | 1.083 |
| Vietnam | F | 2 | 0.933 | 0.827 | 1.052 |
| Vietnam | M | 2 | 0.985 | 0.888 | 1.092 |
| Native Danes (reference risk) | F | 0 | 0.431 | 0.428 | 0.434 |
| Native Danes (reference risk) | M | 0 | 0.433 | 0.430 | 0.436 |

### Monitoring: Analysis of screening for diabetic foot disease

| Monitoring: Analysis of screening for diabetic foot disease | Sex | Model | Estimate | Lower 95% CI | Upper 95% CI |
| --- | --- | --- | --- | --- | --- |
| Middle East | F | 0 | 1.444 | 1.417 | 1.473 |
| Middle East | M | 0 | 1.415 | 1.396 | 1.434 |
| Middle East | F | 1 | 1.349 | 1.322 | 1.376 |
| Middle East | M | 1 | 1.318 | 1.300 | 1.336 |
| Middle East | F | 2 | 1.239 | 1.210 | 1.269 |
| Middle East | M | 2 | 1.261 | 1.241 | 1.282 |
| Europe | F | 0 | 1.151 | 1.118 | 1.184 |
| Europe | M | 0 | 1.142 | 1.115 | 1.170 |
| Europe | F | 1 | 1.133 | 1.103 | 1.164 |
| Europe | M | 1 | 1.119 | 1.094 | 1.144 |
| Europe | F | 2 | 1.107 | 1.076 | 1.138 |
| Europe | M | 2 | 1.112 | 1.086 | 1.139 |
| Turkey | F | 0 | 1.449 | 1.418 | 1.481 |
| Turkey | M | 0 | 1.399 | 1.371 | 1.427 |
| Turkey | F | 1 | 1.380 | 1.348 | 1.413 |
| Turkey | M | 1 | 1.283 | 1.257 | 1.310 |
| Turkey | F | 2 | 1.270 | 1.239 | 1.302 |
| Turkey | M | 2 | 1.227 | 1.201 | 1.253 |
| Former Yugoslavia | F | 0 | 1.410 | 1.372 | 1.448 |
| Former Yugoslavia | M | 0 | 1.410 | 1.381 | 1.440 |
| Former Yugoslavia | F | 1 | 1.377 | 1.339 | 1.415 |
| Former Yugoslavia | M | 1 | 1.334 | 1.305 | 1.363 |
| Former Yugoslavia | F | 2 | 1.296 | 1.260 | 1.334 |
| Former Yugoslavia | M | 2 | 1.300 | 1.271 | 1.329 |
| Pakistan | F | 0 | 1.415 | 1.372 | 1.460 |
| Pakistan | M | 0 | 1.404 | 1.371 | 1.437 |
| Pakistan | F | 1 | 1.394 | 1.350 | 1.439 |
| Pakistan | M | 1 | 1.377 | 1.343 | 1.411 |
| Pakistan | F | 2 | 1.242 | 1.200 | 1.285 |
| Pakistan | M | 2 | 1.281 | 1.249 | 1.315 |
| Sri Lanka | F | 0 | 1.111 | 1.047 | 1.180 |
| Sri Lanka | M | 0 | 1.192 | 1.143 | 1.243 |
| Sri Lanka | F | 1 | 1.024 | 0.965 | 1.086 |
| Sri Lanka | M | 1 | 1.136 | 1.091 | 1.184 |
| Sri Lanka | F | 2 | 0.990 | 0.933 | 1.050 |
| Sri Lanka | M | 2 | 1.120 | 1.074 | 1.167 |
| Somalia | F | 0 | 1.499 | 1.431 | 1.570 |
| Somalia | M | 0 | 1.555 | 1.516 | 1.596 |
| Somalia | F | 1 | 1.289 | 1.227 | 1.354 |
| Somalia | M | 1 | 1.359 | 1.320 | 1.399 |
| Somalia | F | 2 | 1.163 | 1.104 | 1.224 |
| Somalia | M | 2 | 1.268 | 1.229 | 1.308 |
| Vietnam | F | 0 | 1.555 | 1.489 | 1.624 |
| Vietnam | M | 0 | 1.485 | 1.432 | 1.541 |
| Vietnam | F | 1 | 1.507 | 1.438 | 1.580 |
| Vietnam | M | 1 | 1.383 | 1.330 | 1.438 |
| Vietnam | F | 2 | 1.418 | 1.352 | 1.487 |
| Vietnam | M | 2 | 1.342 | 1.292 | 1.395 |
| Native Danes (reference risk) | F | 0 | 0.544 | 0.541 | 0.547 |
| Native Danes (reference risk) | M | 0 | 0.590 | 0.587 | 0.593 |

### Biomarker levels: Analysis of HbA1c levels

| Biomarker levels: Analysis of HbA1c levels | Sex | Model | Estimate | Lower 95% CI | Upper 95% CI |
| --- | --- | --- | --- | --- | --- |
| Middle East | F | 0 | 1.359 | 1.306 | 1.414 |
| Middle East | M | 0 | 1.204 | 1.168 | 1.241 |
| Middle East | F | 1 | 1.189 | 1.144 | 1.235 |
| Middle East | M | 1 | 1.062 | 1.031 | 1.093 |
| Middle East | F | 2 | 1.109 | 1.063 | 1.158 |
| Middle East | M | 2 | 1.031 | 0.998 | 1.065 |
| Europe | F | 0 | 1.013 | 0.963 | 1.067 |
| Europe | M | 0 | 1.042 | 1.000 | 1.085 |
| Europe | F | 1 | 1.080 | 1.029 | 1.134 |
| Europe | M | 1 | 1.108 | 1.066 | 1.152 |
| Europe | F | 2 | 1.030 | 0.980 | 1.083 |
| Europe | M | 2 | 1.063 | 1.021 | 1.107 |
| Turkey | F | 0 | 1.508 | 1.447 | 1.572 |
| Turkey | M | 0 | 1.415 | 1.362 | 1.469 |
| Turkey | F | 1 | 1.310 | 1.260 | 1.361 |
| Turkey | M | 1 | 1.265 | 1.221 | 1.311 |
| Turkey | F | 2 | 1.275 | 1.224 | 1.328 |
| Turkey | M | 2 | 1.247 | 1.202 | 1.294 |
| Former Yugoslavia | F | 0 | 1.437 | 1.367 | 1.511 |
| Former Yugoslavia | M | 0 | 1.254 | 1.196 | 1.314 |
| Former Yugoslavia | F | 1 | 1.338 | 1.276 | 1.403 |
| Former Yugoslavia | M | 1 | 1.163 | 1.112 | 1.216 |
| Former Yugoslavia | F | 2 | 1.271 | 1.211 | 1.334 |
| Former Yugoslavia | M | 2 | 1.145 | 1.094 | 1.198 |
| Pakistan | F | 0 | 1.549 | 1.466 | 1.636 |
| Pakistan | M | 0 | 1.409 | 1.345 | 1.475 |
| Pakistan | F | 1 | 1.274 | 1.211 | 1.340 |
| Pakistan | M | 1 | 1.203 | 1.152 | 1.256 |
| Pakistan | F | 2 | 1.224 | 1.160 | 1.291 |
| Pakistan | M | 2 | 1.151 | 1.100 | 1.204 |
| Sri Lanka | F | 0 | 1.405 | 1.299 | 1.520 |
| Sri Lanka | M | 0 | 1.362 | 1.283 | 1.445 |
| Sri Lanka | F | 1 | 1.141 | 1.061 | 1.227 |
| Sri Lanka | M | 1 | 1.052 | 0.995 | 1.112 |
| Sri Lanka | F | 2 | 1.085 | 1.008 | 1.167 |
| Sri Lanka | M | 2 | 1.035 | 0.978 | 1.095 |
| Somalia | F | 0 | 1.358 | 1.222 | 1.509 |
| Somalia | M | 0 | 1.314 | 1.212 | 1.426 |
| Somalia | F | 1 | 1.216 | 1.100 | 1.345 |
| Somalia | M | 1 | 1.076 | 0.997 | 1.162 |
| Somalia | F | 2 | 1.112 | 1.001 | 1.234 |
| Somalia | M | 2 | 1.025 | 0.947 | 1.110 |
| Vietnam | F | 0 | 1.023 | 0.888 | 1.178 |
| Vietnam | M | 0 | 0.957 | 0.842 | 1.087 |
| Vietnam | F | 1 | 0.984 | 0.861 | 1.124 |
| Vietnam | M | 1 | 0.907 | 0.803 | 1.026 |
| Vietnam | F | 2 | 0.908 | 0.794 | 1.038 |
| Vietnam | M | 2 | 0.863 | 0.765 | 0.975 |
| Native Danes (reference risk) | F | 0 | 0.338 | 0.335 | 0.341 |
| Native Danes (reference risk) | M | 0 | 0.399 | 0.396 | 0.402 |

### Biomarker levels: Analysis of LDL-C levels

| Biomarker levels: Analysis of LDL-C levels | Sex | Model | Estimate | Lower 95% CI | Upper 95% CI |
| --- | --- | --- | --- | --- | --- |
| Middle East | F | 0 | 1.115 | 1.063 | 1.170 |
| Middle East | M | 0 | 1.251 | 1.197 | 1.307 |
| Middle East | F | 1 | 1.041 | 0.993 | 1.092 |
| Middle East | M | 1 | 1.134 | 1.086 | 1.184 |
| Middle East | F | 2 | 1.026 | 0.971 | 1.083 |
| Middle East | M | 2 | 1.147 | 1.093 | 1.205 |
| Europe | F | 0 | 1.166 | 1.113 | 1.223 |
| Europe | M | 0 | 1.167 | 1.105 | 1.232 |
| Europe | F | 1 | 1.147 | 1.095 | 1.202 |
| Europe | M | 1 | 1.141 | 1.082 | 1.203 |
| Europe | F | 2 | 1.134 | 1.080 | 1.190 |
| Europe | M | 2 | 1.166 | 1.102 | 1.233 |
| Turkey | F | 0 | 1.039 | 0.981 | 1.102 |
| Turkey | M | 0 | 1.246 | 1.169 | 1.328 |
| Turkey | F | 1 | 0.999 | 0.944 | 1.058 |
| Turkey | M | 1 | 1.112 | 1.045 | 1.184 |
| Turkey | F | 2 | 0.967 | 0.911 | 1.025 |
| Turkey | M | 2 | 1.084 | 1.017 | 1.155 |
| Former Yugoslavia | F | 0 | 0.921 | 0.856 | 0.992 |
| Former Yugoslavia | M | 0 | 1.252 | 1.167 | 1.343 |
| Former Yugoslavia | F | 1 | 0.913 | 0.849 | 0.982 |
| Former Yugoslavia | M | 1 | 1.176 | 1.099 | 1.259 |
| Former Yugoslavia | F | 2 | 0.896 | 0.832 | 0.964 |
| Former Yugoslavia | M | 2 | 1.164 | 1.087 | 1.247 |
| Pakistan | F | 0 | 1.014 | 0.936 | 1.099 |
| Pakistan | M | 0 | 1.265 | 1.172 | 1.365 |
| Pakistan | F | 1 | 1.008 | 0.932 | 1.091 |
| Pakistan | M | 1 | 1.248 | 1.159 | 1.343 |
| Pakistan | F | 2 | 0.947 | 0.874 | 1.028 |
| Pakistan | M | 2 | 1.201 | 1.113 | 1.296 |
| Sri Lanka | F | 0 | 1.012 | 0.911 | 1.124 |
| Sri Lanka | M | 0 | 1.012 | 0.905 | 1.132 |
| Sri Lanka | F | 1 | 0.922 | 0.831 | 1.024 |
| Sri Lanka | M | 1 | 0.929 | 0.834 | 1.036 |
| Sri Lanka | F | 2 | 0.941 | 0.847 | 1.045 |
| Sri Lanka | M | 2 | 0.942 | 0.845 | 1.049 |
| Somalia | F | 0 | 1.673 | 1.531 | 1.829 |
| Somalia | M | 0 | 1.897 | 1.732 | 2.078 |
| Somalia | F | 1 | 1.399 | 1.279 | 1.530 |
| Somalia | M | 1 | 1.482 | 1.354 | 1.623 |
| Somalia | F | 2 | 1.389 | 1.263 | 1.528 |
| Somalia | M | 2 | 1.492 | 1.358 | 1.639 |
| Vietnam | F | 0 | 0.763 | 0.639 | 0.911 |
| Vietnam | M | 0 | 1.087 | 0.923 | 1.279 |
| Vietnam | F | 1 | 0.732 | 0.616 | 0.870 |
| Vietnam | M | 1 | 0.951 | 0.811 | 1.114 |
| Vietnam | F | 2 | 0.736 | 0.618 | 0.876 |
| Vietnam | M | 2 | 0.948 | 0.809 | 1.111 |
| Native Danes (reference risk) | F | 0 | 0.328 | 0.325 | 0.331 |
| Native Danes (reference risk) | M | 0 | 0.248 | 0.245 | 0.250 |

### Pharmacological treatment: Analysis of glucose-lowering drugs

| Pharmacological treatment: Analysis of glucose-lowering drugs | Sex | Model | Estimate | Lower 95% CI | Upper 95% CI |
| --- | --- | --- | --- | --- | --- |
| Middle East | F | 0 | 0.693 | 0.573 | 0.838 |
| Middle East | M | 0 | 1.114 | 0.975 | 1.272 |
| Middle East | F | 1 | 0.935 | 0.778 | 1.123 |
| Middle East | M | 1 | 1.285 | 1.128 | 1.463 |
| Middle East | F | 2 | 0.835 | 0.681 | 1.025 |
| Middle East | M | 2 | 1.128 | 0.971 | 1.311 |
| Europe | F | 0 | 1.543 | 1.345 | 1.771 |
| Europe | M | 0 | 1.334 | 1.148 | 1.549 |
| Europe | F | 1 | 1.350 | 1.187 | 1.535 |
| Europe | M | 1 | 1.198 | 1.036 | 1.385 |
| Europe | F | 2 | 1.272 | 1.114 | 1.453 |
| Europe | M | 2 | 1.171 | 1.007 | 1.361 |
| Turkey | F | 0 | 0.592 | 0.471 | 0.743 |
| Turkey | M | 0 | 0.892 | 0.725 | 1.097 |
| Turkey | F | 1 | 0.857 | 0.688 | 1.067 |
| Turkey | M | 1 | 1.006 | 0.819 | 1.236 |
| Turkey | F | 2 | 0.724 | 0.577 | 0.910 |
| Turkey | M | 2 | 0.804 | 0.652 | 0.991 |
| Former Yugoslavia | F | 0 | 0.687 | 0.536 | 0.880 |
| Former Yugoslavia | M | 0 | 0.981 | 0.786 | 1.226 |
| Former Yugoslavia | F | 1 | 0.834 | 0.658 | 1.057 |
| Former Yugoslavia | M | 1 | 1.049 | 0.848 | 1.298 |
| Former Yugoslavia | F | 2 | 0.757 | 0.593 | 0.965 |
| Former Yugoslavia | M | 2 | 0.929 | 0.750 | 1.152 |
| Pakistan | F | 0 | 0.911 | 0.714 | 1.163 |
| Pakistan | M | 0 | 1.362 | 1.111 | 1.668 |
| Pakistan | F | 1 | 1.506 | 1.195 | 1.897 |
| Pakistan | M | 1 | 1.782 | 1.469 | 2.162 |
| Pakistan | F | 2 | 1.173 | 0.924 | 1.488 |
| Pakistan | M | 2 | 1.297 | 1.060 | 1.587 |
| Sri Lanka | F | 0 | 0.498 | 0.320 | 0.775 |
| Sri Lanka | M | 0 | 0.705 | 0.496 | 1.001 |
| Sri Lanka | F | 1 | 0.801 | 0.527 | 1.217 |
| Sri Lanka | M | 1 | 1.095 | 0.782 | 1.534 |
| Sri Lanka | F | 2 | 0.812 | 0.532 | 1.239 |
| Sri Lanka | M | 2 | 1.118 | 0.797 | 1.568 |
| Somalia | F | 0 | 1.596 | 1.152 | 2.209 |
| Somalia | M | 0 | 1.740 | 1.287 | 2.353 |
| Somalia | F | 1 | 1.896 | 1.386 | 2.593 |
| Somalia | M | 1 | 1.883 | 1.388 | 2.555 |
| Somalia | F | 2 | 1.705 | 1.214 | 2.395 |
| Somalia | M | 2 | 1.619 | 1.174 | 2.232 |
| Vietnam | F | 0 | 0.883 | 0.541 | 1.440 |
| Vietnam | M | 0 | 1.200 | 0.769 | 1.874 |
| Vietnam | F | 1 | 0.923 | 0.565 | 1.508 |
| Vietnam | M | 1 | 1.245 | 0.820 | 1.892 |
| Vietnam | F | 2 | 0.906 | 0.550 | 1.493 |
| Vietnam | M | 2 | 1.213 | 0.794 | 1.852 |
| Native Danes (reference risk) | F | 0 | 0.079 | 0.076 | 0.081 |
| Native Danes (reference risk) | M | 0 | 0.062 | 0.060 | 0.064 |

### Pharmacological treatment: Analysis of lipid-lowering drugs

| Pharmacological treatment: Analysis of lipid-lowering drugs | Sex | Model | Estimate | Lower 95% CI | Upper 95% CI |
| --- | --- | --- | --- | --- | --- |
| Middle East | F | 0 | 0.941 | 0.884 | 1.002 |
| Middle East | M | 0 | 1.096 | 1.036 | 1.158 |
| Middle East | F | 1 | 0.930 | 0.877 | 0.986 |
| Middle East | M | 1 | 1.029 | 0.978 | 1.083 |
| Middle East | F | 2 | 0.944 | 0.885 | 1.007 |
| Middle East | M | 2 | 0.977 | 0.923 | 1.035 |
| Europe | F | 0 | 1.145 | 1.085 | 1.207 |
| Europe | M | 0 | 1.100 | 1.030 | 1.176 |
| Europe | F | 1 | 1.101 | 1.049 | 1.156 |
| Europe | M | 1 | 1.075 | 1.012 | 1.142 |
| Europe | F | 2 | 1.106 | 1.051 | 1.163 |
| Europe | M | 2 | 1.080 | 1.013 | 1.152 |
| Turkey | F | 0 | 0.810 | 0.747 | 0.877 |
| Turkey | M | 0 | 0.995 | 0.912 | 1.085 |
| Turkey | F | 1 | 0.833 | 0.772 | 0.898 |
| Turkey | M | 1 | 0.942 | 0.869 | 1.021 |
| Turkey | F | 2 | 0.815 | 0.755 | 0.881 |
| Turkey | M | 2 | 0.870 | 0.801 | 0.944 |
| Former Yugoslavia | F | 0 | 0.736 | 0.665 | 0.816 |
| Former Yugoslavia | M | 0 | 0.890 | 0.805 | 0.985 |
| Former Yugoslavia | F | 1 | 0.832 | 0.757 | 0.914 |
| Former Yugoslavia | M | 1 | 0.866 | 0.790 | 0.949 |
| Former Yugoslavia | F | 2 | 0.840 | 0.763 | 0.923 |
| Former Yugoslavia | M | 2 | 0.832 | 0.758 | 0.914 |
| Pakistan | F | 0 | 0.897 | 0.808 | 0.996 |
| Pakistan | M | 0 | 0.930 | 0.836 | 1.034 |
| Pakistan | F | 1 | 0.955 | 0.865 | 1.054 |
| Pakistan | M | 1 | 0.946 | 0.859 | 1.041 |
| Pakistan | F | 2 | 0.917 | 0.829 | 1.014 |
| Pakistan | M | 2 | 0.864 | 0.783 | 0.954 |
| Sri Lanka | F | 0 | 0.959 | 0.841 | 1.092 |
| Sri Lanka | M | 0 | 0.835 | 0.714 | 0.978 |
| Sri Lanka | F | 1 | 0.926 | 0.818 | 1.050 |
| Sri Lanka | M | 1 | 0.788 | 0.684 | 0.908 |
| Sri Lanka | F | 2 | 0.976 | 0.862 | 1.105 |
| Sri Lanka | M | 2 | 0.814 | 0.706 | 0.938 |
| Somalia | F | 0 | 1.391 | 1.247 | 1.552 |
| Somalia | M | 0 | 1.892 | 1.710 | 2.094 |
| Somalia | F | 1 | 1.040 | 0.935 | 1.157 |
| Somalia | M | 1 | 1.285 | 1.159 | 1.424 |
| Somalia | F | 2 | 1.092 | 0.976 | 1.222 |
| Somalia | M | 2 | 1.230 | 1.105 | 1.369 |
| Vietnam | F | 0 | 0.797 | 0.641 | 0.992 |
| Vietnam | M | 0 | 0.886 | 0.700 | 1.121 |
| Vietnam | F | 1 | 0.817 | 0.668 | 1.000 |
| Vietnam | M | 1 | 0.749 | 0.599 | 0.936 |
| Vietnam | F | 2 | 0.859 | 0.700 | 1.053 |
| Vietnam | M | 2 | 0.755 | 0.605 | 0.942 |
| Native Danes (reference risk) | F | 0 | 0.415 | 0.411 | 0.420 |
| Native Danes (reference risk) | M | 0 | 0.300 | 0.296 | 0.303 |

### Pharmacological treatment: Analysis of ACEI/ARB

| Pharmacological treatment: Analysis of ACEI/ARB | Sex | Model | Estimate | Lower 95% CI | Upper 95% CI |
| --- | --- | --- | --- | --- | --- |
| Middle East | F | 0 | 1.094 | 0.994 | 1.204 |
| Middle East | M | 0 | 1.368 | 1.278 | 1.465 |
| Middle East | F | 1 | 1.097 | 0.998 | 1.205 |
| Middle East | M | 1 | 1.422 | 1.328 | 1.522 |
| Middle East | F | 2 | 1.020 | 0.917 | 1.135 |
| Middle East | M | 2 | 1.306 | 1.209 | 1.411 |
| Europe | F | 0 | 0.996 | 0.896 | 1.106 |
| Europe | M | 0 | 1.031 | 0.936 | 1.135 |
| Europe | F | 1 | 0.982 | 0.884 | 1.090 |
| Europe | M | 1 | 1.033 | 0.939 | 1.136 |
| Europe | F | 2 | 0.964 | 0.866 | 1.072 |
| Europe | M | 2 | 1.019 | 0.924 | 1.122 |
| Turkey | F | 0 | 0.981 | 0.873 | 1.102 |
| Turkey | M | 0 | 1.458 | 1.327 | 1.601 |
| Turkey | F | 1 | 1.005 | 0.897 | 1.127 |
| Turkey | M | 1 | 1.476 | 1.347 | 1.618 |
| Turkey | F | 2 | 0.939 | 0.835 | 1.057 |
| Turkey | M | 2 | 1.355 | 1.233 | 1.489 |
| Former Yugoslavia | F | 0 | 0.933 | 0.818 | 1.065 |
| Former Yugoslavia | M | 0 | 1.163 | 1.034 | 1.309 |
| Former Yugoslavia | F | 1 | 0.940 | 0.826 | 1.070 |
| Former Yugoslavia | M | 1 | 1.198 | 1.066 | 1.345 |
| Former Yugoslavia | F | 2 | 0.885 | 0.775 | 1.011 |
| Former Yugoslavia | M | 2 | 1.099 | 0.977 | 1.237 |
| Pakistan | F | 0 | 1.095 | 0.941 | 1.274 |
| Pakistan | M | 0 | 1.443 | 1.297 | 1.605 |
| Pakistan | F | 1 | 1.181 | 1.017 | 1.372 |
| Pakistan | M | 1 | 1.558 | 1.403 | 1.731 |
| Pakistan | F | 2 | 1.091 | 0.933 | 1.275 |
| Pakistan | M | 2 | 1.428 | 1.279 | 1.594 |
| Sri Lanka | F | 0 | 1.347 | 1.133 | 1.601 |
| Sri Lanka | M | 0 | 1.230 | 1.034 | 1.463 |
| Sri Lanka | F | 1 | 1.366 | 1.162 | 1.605 |
| Sri Lanka | M | 1 | 1.379 | 1.166 | 1.631 |
| Sri Lanka | F | 2 | 1.341 | 1.138 | 1.581 |
| Sri Lanka | M | 2 | 1.326 | 1.119 | 1.571 |
| Somalia | F | 0 | 1.024 | 0.696 | 1.507 |
| Somalia | M | 0 | 1.840 | 1.488 | 2.276 |
| Somalia | F | 1 | 0.976 | 0.675 | 1.411 |
| Somalia | M | 1 | 1.879 | 1.532 | 2.305 |
| Somalia | F | 2 | 0.896 | 0.615 | 1.305 |
| Somalia | M | 2 | 1.696 | 1.380 | 2.084 |
| Vietnam | F | 0 | 0.692 | 0.471 | 1.018 |
| Vietnam | M | 0 | 1.015 | 0.742 | 1.389 |
| Vietnam | F | 1 | 0.742 | 0.511 | 1.078 |
| Vietnam | M | 1 | 1.094 | 0.808 | 1.482 |
| Vietnam | F | 2 | 0.731 | 0.504 | 1.061 |
| Vietnam | M | 2 | 1.047 | 0.771 | 1.421 |
| Native Danes (reference risk) | F | 0 | 0.332 | 0.326 | 0.338 |
| Native Danes (reference risk) | M | 0 | 0.257 | 0.253 | 0.261 |

### Pharmacological treatment: Analysis of antiplatelet therapy

| Pharmacological treatment: Analysis of antiplatelet therapy | Sex | Model | Estimate | Lower 95% CI | Upper 95% CI |
| --- | --- | --- | --- | --- | --- |
| Middle East | F | 0 | 1.246 | 1.154 | 1.345 |
| Middle East | M | 0 | 0.929 | 0.862 | 1.001 |
| Middle East | F | 1 | 1.208 | 1.121 | 1.302 |
| Middle East | M | 1 | 0.957 | 0.890 | 1.029 |
| Middle East | F | 2 | 1.233 | 1.133 | 1.342 |
| Middle East | M | 2 | 0.918 | 0.846 | 0.995 |
| Europe | F | 0 | 1.086 | 0.994 | 1.186 |
| Europe | M | 0 | 0.987 | 0.908 | 1.072 |
| Europe | F | 1 | 1.101 | 1.010 | 1.200 |
| Europe | M | 1 | 1.015 | 0.936 | 1.102 |
| Europe | F | 2 | 1.100 | 1.008 | 1.200 |
| Europe | M | 2 | 1.007 | 0.926 | 1.094 |
| Turkey | F | 0 | 1.012 | 0.914 | 1.121 |
| Turkey | M | 0 | 0.878 | 0.785 | 0.984 |
| Turkey | F | 1 | 1.016 | 0.920 | 1.123 |
| Turkey | M | 1 | 0.899 | 0.804 | 1.004 |
| Turkey | F | 2 | 1.033 | 0.931 | 1.145 |
| Turkey | M | 2 | 0.864 | 0.771 | 0.967 |
| Former Yugoslavia | F | 0 | 1.158 | 1.047 | 1.280 |
| Former Yugoslavia | M | 0 | 0.986 | 0.883 | 1.102 |
| Former Yugoslavia | F | 1 | 1.176 | 1.068 | 1.296 |
| Former Yugoslavia | M | 1 | 0.998 | 0.896 | 1.111 |
| Former Yugoslavia | F | 2 | 1.199 | 1.086 | 1.325 |
| Former Yugoslavia | M | 2 | 0.968 | 0.867 | 1.080 |
| Pakistan | F | 0 | 1.026 | 0.889 | 1.184 |
| Pakistan | M | 0 | 0.708 | 0.611 | 0.820 |
| Pakistan | F | 1 | 1.117 | 0.977 | 1.278 |
| Pakistan | M | 1 | 0.759 | 0.657 | 0.876 |
| Pakistan | F | 2 | 1.105 | 0.961 | 1.270 |
| Pakistan | M | 2 | 0.713 | 0.616 | 0.825 |
| Sri Lanka | F | 0 | 1.224 | 1.036 | 1.445 |
| Sri Lanka | M | 0 | 0.764 | 0.623 | 0.935 |
| Sri Lanka | F | 1 | 1.157 | 0.987 | 1.356 |
| Sri Lanka | M | 1 | 0.765 | 0.631 | 0.926 |
| Sri Lanka | F | 2 | 1.227 | 1.046 | 1.439 |
| Sri Lanka | M | 2 | 0.783 | 0.646 | 0.949 |
| Somalia | F | 0 | 1.681 | 1.365 | 2.071 |
| Somalia | M | 0 | 1.436 | 1.161 | 1.776 |
| Somalia | F | 1 | 1.322 | 1.059 | 1.652 |
| Somalia | M | 1 | 1.366 | 1.119 | 1.669 |
| Somalia | F | 2 | 1.400 | 1.116 | 1.755 |
| Somalia | M | 2 | 1.305 | 1.062 | 1.603 |
| Vietnam | F | 0 | 1.208 | 0.961 | 1.518 |
| Vietnam | M | 0 | 1.011 | 0.777 | 1.315 |
| Vietnam | F | 1 | 1.086 | 0.884 | 1.335 |
| Vietnam | M | 1 | 0.853 | 0.669 | 1.087 |
| Vietnam | F | 2 | 1.150 | 0.935 | 1.415 |
| Vietnam | M | 2 | 0.862 | 0.675 | 1.100 |
| Native Danes (reference risk) | F | 0 | 0.381 | 0.375 | 0.387 |
| Native Danes (reference risk) | M | 0 | 0.330 | 0.325 | 0.334 |
