# Supplementary material for: Guideline-level monitoring, biomarker levels and pharmacological treatment in migrants and native Danes with type 2 diabetes: Population-wide analyses
Source: PLOS Glob Public Health. 2023 Oct 18;3(10):e0001277. doi: 10.1371/journal.pgph.0001277 (PMC10584163; doi:10.1371/journal.pgph.0001277)
Supplement: S4 File — (HTML) [file pgph.0001277.s004.html]

S4. Supplementary analyses of monitoring stratified by diabetes duration.


# S4. Supplementary analyses of monitoring stratified by diabetes duration.

- S4. Supplementary analyses of monitoring stratified by diabetes duration.
  - HbA1c monitoring
  - LDL-C monitoring
  - Screening for diabetic nephropathy
  - Screening for diabetic retinopathy
  - Screening for diabetic foot disease

# S4. Supplementary analyses of monitoring stratified by diabetes duration.

Note: All individuals in the Sri Lanka group with a diabetes duration shorter than 15 months received HbA1c monitoring.

### HbA1c monitoring

| HbA1c monitoring | Model | Estimate | Lower 95% CI | Upper 95% CI | Diabetes duration relative to routine monitoring window |
| --- | --- | --- | --- | --- | --- |
| Middle East | 0 | 1.162 | 1.075 | 1.255 | Longer or equal |
| Middle East | 1 | 0.952 | 0.881 | 1.029 | Longer or equal |
| Middle East | 2 | 0.892 | 0.817 | 0.974 | Longer or equal |
| Europe | 0 | 1.326 | 1.224 | 1.436 | Longer or equal |
| Europe | 1 | 1.326 | 1.226 | 1.435 | Longer or equal |
| Europe | 2 | 1.277 | 1.176 | 1.386 | Longer or equal |
| Turkey | 0 | 0.978 | 0.875 | 1.093 | Longer or equal |
| Turkey | 1 | 0.796 | 0.712 | 0.890 | Longer or equal |
| Turkey | 2 | 0.673 | 0.601 | 0.754 | Longer or equal |
| Former Yugoslavia | 0 | 0.963 | 0.849 | 1.093 | Longer or equal |
| Former Yugoslavia | 1 | 0.873 | 0.769 | 0.990 | Longer or equal |
| Former Yugoslavia | 2 | 0.841 | 0.740 | 0.955 | Longer or equal |
| Pakistan | 0 | 1.087 | 0.952 | 1.241 | Longer or equal |
| Pakistan | 1 | 0.943 | 0.826 | 1.077 | Longer or equal |
| Pakistan | 2 | 0.683 | 0.596 | 0.783 | Longer or equal |
| Sri Lanka | 0 | 0.643 | 0.513 | 0.806 | Longer or equal |
| Sri Lanka | 1 | 0.503 | 0.400 | 0.631 | Longer or equal |
| Sri Lanka | 2 | 0.558 | 0.444 | 0.700 | Longer or equal |
| Somalia | 0 | 1.622 | 1.353 | 1.945 | Longer or equal |
| Somalia | 1 | 1.076 | 0.895 | 1.294 | Longer or equal |
| Somalia | 2 | 1.032 | 0.852 | 1.250 | Longer or equal |
| Vietnam | 0 | 1.379 | 1.107 | 1.719 | Longer or equal |
| Vietnam | 1 | 1.234 | 0.992 | 1.535 | Longer or equal |
| Vietnam | 2 | 1.280 | 1.029 | 1.591 | Longer or equal |
| (Intercept) | 0 | 0.072 | 0.071 | 0.073 | Longer or equal |
| Middle East | 0 | 2.190 | 1.228 | 3.905 | Shorter |
| Middle East | 1 | 1.820 | 1.014 | 3.268 | Shorter |
| Middle East | 2 | 1.540 | 0.670 | 3.539 | Shorter |
| Europe | 0 | 1.837 | 0.977 | 3.454 | Shorter |
| Europe | 1 | 1.781 | 0.956 | 3.318 | Shorter |
| Europe | 2 | 1.091 | 0.564 | 2.110 | Shorter |
| Turkey | 0 | 1.223 | 0.457 | 3.273 | Shorter |
| Turkey | 1 | 1.007 | 0.377 | 2.694 | Shorter |
| Turkey | 2 | 0.861 | 0.310 | 2.393 | Shorter |
| Former Yugoslavia | 0 | 0.733 | 0.183 | 2.938 | Shorter |
| Former Yugoslavia | 1 | 0.674 | 0.169 | 2.688 | Shorter |
| Former Yugoslavia | 2 | 0.655 | 0.164 | 2.625 | Shorter |
| Pakistan | 0 | 2.485 | 0.807 | 7.654 | Shorter |
| Pakistan | 1 | 1.991 | 0.639 | 6.204 | Shorter |
| Pakistan | 2 | 0.956 | 0.254 | 3.605 | Shorter |
| Sri Lanka | 0 | 0.000 | 0.000 | 0.000 | Shorter |
| Sri Lanka | 1 | 0.000 | 0.000 | 0.000 | Shorter |
| Sri Lanka | 2 | 0.000 | 0.000 | 0.000 | Shorter |
| Somalia | 0 | 2.589 | 0.656 | 10.214 | Shorter |
| Somalia | 1 | 1.475 | 0.394 | 5.516 | Shorter |
| Somalia | 2 | 1.592 | 0.457 | 5.540 | Shorter |
| Vietnam | 0 | 3.126 | 0.796 | 12.278 | Shorter |
| Vietnam | 1 | 2.917 | 0.751 | 11.339 | Shorter |
| Vietnam | 2 | 4.008 | 0.923 | 17.405 | Shorter |
| (Intercept) | 0 | 0.012 | 0.010 | 0.014 | Shorter |

### LDL-C monitoring

| LDL-C monitoring | Model | Estimate | Lower 95% CI | Upper 95% CI | Diabetes duration relative to routine monitoring window |
| --- | --- | --- | --- | --- | --- |
| Middle East | 0 | 1.165 | 1.104 | 1.229 | Longer or equal |
| Middle East | 1 | 1.017 | 0.964 | 1.074 | Longer or equal |
| Middle East | 2 | 0.892 | 0.840 | 0.948 | Longer or equal |
| Europe | 0 | 1.177 | 1.109 | 1.250 | Longer or equal |
| Europe | 1 | 1.187 | 1.120 | 1.259 | Longer or equal |
| Europe | 2 | 1.127 | 1.060 | 1.198 | Longer or equal |
| Turkey | 0 | 1.173 | 1.093 | 1.259 | Longer or equal |
| Turkey | 1 | 1.027 | 0.957 | 1.102 | Longer or equal |
| Turkey | 2 | 0.828 | 0.771 | 0.890 | Longer or equal |
| Former Yugoslavia | 0 | 0.959 | 0.877 | 1.048 | Longer or equal |
| Former Yugoslavia | 1 | 0.910 | 0.832 | 0.995 | Longer or equal |
| Former Yugoslavia | 2 | 0.842 | 0.770 | 0.921 | Longer or equal |
| Pakistan | 0 | 1.192 | 1.092 | 1.302 | Longer or equal |
| Pakistan | 1 | 1.101 | 1.008 | 1.203 | Longer or equal |
| Pakistan | 2 | 0.768 | 0.702 | 0.841 | Longer or equal |
| Sri Lanka | 0 | 0.685 | 0.586 | 0.801 | Longer or equal |
| Sri Lanka | 1 | 0.572 | 0.489 | 0.668 | Longer or equal |
| Sri Lanka | 2 | 0.612 | 0.524 | 0.715 | Longer or equal |
| Somalia | 0 | 1.397 | 1.218 | 1.601 | Longer or equal |
| Somalia | 1 | 1.020 | 0.889 | 1.171 | Longer or equal |
| Somalia | 2 | 0.921 | 0.800 | 1.060 | Longer or equal |
| Vietnam | 0 | 0.939 | 0.776 | 1.137 | Longer or equal |
| Vietnam | 1 | 0.860 | 0.711 | 1.040 | Longer or equal |
| Vietnam | 2 | 0.880 | 0.729 | 1.063 | Longer or equal |
| (Intercept) | 0 | 0.135 | 0.133 | 0.136 | Longer or equal |
| Middle East | 0 | 1.183 | 0.878 | 1.593 | Shorter |
| Middle East | 1 | 1.113 | 0.824 | 1.503 | Shorter |
| Middle East | 2 | 1.005 | 0.710 | 1.422 | Shorter |
| Europe | 0 | 1.278 | 0.960 | 1.703 | Shorter |
| Europe | 1 | 1.247 | 0.938 | 1.657 | Shorter |
| Europe | 2 | 1.197 | 0.874 | 1.639 | Shorter |
| Turkey | 0 | 1.257 | 0.868 | 1.820 | Shorter |
| Turkey | 1 | 1.211 | 0.839 | 1.748 | Shorter |
| Turkey | 2 | 0.975 | 0.670 | 1.419 | Shorter |
| Former Yugoslavia | 0 | 0.696 | 0.400 | 1.210 | Shorter |
| Former Yugoslavia | 1 | 0.709 | 0.408 | 1.235 | Shorter |
| Former Yugoslavia | 2 | 0.697 | 0.399 | 1.217 | Shorter |
| Pakistan | 0 | 1.310 | 0.726 | 2.366 | Shorter |
| Pakistan | 1 | 1.229 | 0.674 | 2.241 | Shorter |
| Pakistan | 2 | 0.913 | 0.485 | 1.719 | Shorter |
| Sri Lanka | 0 | 1.268 | 0.680 | 2.365 | Shorter |
| Sri Lanka | 1 | 1.167 | 0.625 | 2.179 | Shorter |
| Sri Lanka | 2 | 1.142 | 0.607 | 2.147 | Shorter |
| Somalia | 0 | 1.638 | 0.854 | 3.140 | Shorter |
| Somalia | 1 | 1.256 | 0.661 | 2.388 | Shorter |
| Somalia | 2 | 1.174 | 0.613 | 2.248 | Shorter |
| Vietnam | 0 | 1.483 | 0.697 | 3.158 | Shorter |
| Vietnam | 1 | 1.457 | 0.686 | 3.094 | Shorter |
| Vietnam | 2 | 1.549 | 0.736 | 3.259 | Shorter |
| (Intercept) | 0 | 0.076 | 0.072 | 0.081 | Shorter |

### Screening for diabetic nephropathy

| Screening for diabetic nephropathy | Model | Estimate | Lower 95% CI | Upper 95% CI | Diabetes duration relative to routine monitoring window |
| --- | --- | --- | --- | --- | --- |
| Middle East | 0 | 1.116 | 1.089 | 1.143 | Longer or equal |
| Middle East | 1 | 1.109 | 1.083 | 1.136 | Longer or equal |
| Middle East | 2 | 1.022 | 0.995 | 1.050 | Longer or equal |
| Europe | 0 | 1.142 | 1.112 | 1.172 | Longer or equal |
| Europe | 1 | 1.124 | 1.096 | 1.153 | Longer or equal |
| Europe | 2 | 1.088 | 1.060 | 1.117 | Longer or equal |
| Turkey | 0 | 1.071 | 1.036 | 1.107 | Longer or equal |
| Turkey | 1 | 1.052 | 1.018 | 1.087 | Longer or equal |
| Turkey | 2 | 0.935 | 0.905 | 0.967 | Longer or equal |
| Former Yugoslavia | 0 | 1.029 | 0.990 | 1.070 | Longer or equal |
| Former Yugoslavia | 1 | 1.031 | 0.993 | 1.071 | Longer or equal |
| Former Yugoslavia | 2 | 0.963 | 0.928 | 1.001 | Longer or equal |
| Pakistan | 0 | 1.133 | 1.089 | 1.178 | Longer or equal |
| Pakistan | 1 | 1.139 | 1.096 | 1.185 | Longer or equal |
| Pakistan | 2 | 0.928 | 0.891 | 0.966 | Longer or equal |
| Sri Lanka | 0 | 0.777 | 0.725 | 0.834 | Longer or equal |
| Sri Lanka | 1 | 0.779 | 0.727 | 0.834 | Longer or equal |
| Sri Lanka | 2 | 0.871 | 0.814 | 0.932 | Longer or equal |
| Somalia | 0 | 1.306 | 1.232 | 1.384 | Longer or equal |
| Somalia | 1 | 1.218 | 1.148 | 1.291 | Longer or equal |
| Somalia | 2 | 1.172 | 1.102 | 1.246 | Longer or equal |
| Vietnam | 0 | 0.957 | 0.877 | 1.043 | Longer or equal |
| Vietnam | 1 | 0.973 | 0.894 | 1.058 | Longer or equal |
| Vietnam | 2 | 1.008 | 0.927 | 1.096 | Longer or equal |
| (Intercept) | 0 | 0.431 | 0.429 | 0.434 | Longer or equal |
| Middle East | 0 | 1.045 | 0.957 | 1.141 | Shorter |
| Middle East | 1 | 1.074 | 0.984 | 1.173 | Shorter |
| Middle East | 2 | 1.004 | 0.909 | 1.108 | Shorter |
| Europe | 0 | 1.069 | 0.981 | 1.165 | Shorter |
| Europe | 1 | 1.078 | 0.990 | 1.173 | Shorter |
| Europe | 2 | 1.046 | 0.954 | 1.146 | Shorter |
| Turkey | 0 | 1.042 | 0.930 | 1.166 | Shorter |
| Turkey | 1 | 1.041 | 0.930 | 1.165 | Shorter |
| Turkey | 2 | 0.942 | 0.840 | 1.057 | Shorter |
| Former Yugoslavia | 0 | 1.093 | 0.972 | 1.229 | Shorter |
| Former Yugoslavia | 1 | 1.098 | 0.978 | 1.233 | Shorter |
| Former Yugoslavia | 2 | 1.044 | 0.929 | 1.174 | Shorter |
| Pakistan | 0 | 1.176 | 1.001 | 1.381 | Shorter |
| Pakistan | 1 | 1.200 | 1.025 | 1.404 | Shorter |
| Pakistan | 2 | 1.026 | 0.871 | 1.208 | Shorter |
| Sri Lanka | 0 | 0.970 | 0.789 | 1.191 | Shorter |
| Sri Lanka | 1 | 0.986 | 0.804 | 1.209 | Shorter |
| Sri Lanka | 2 | 1.088 | 0.891 | 1.329 | Shorter |
| Somalia | 0 | 1.103 | 0.888 | 1.369 | Shorter |
| Somalia | 1 | 1.121 | 0.903 | 1.392 | Shorter |
| Somalia | 2 | 1.069 | 0.861 | 1.327 | Shorter |
| Vietnam | 0 | 0.777 | 0.557 | 1.083 | Shorter |
| Vietnam | 1 | 0.778 | 0.561 | 1.080 | Shorter |
| Vietnam | 2 | 0.804 | 0.578 | 1.120 | Shorter |
| (Intercept) | 0 | 0.510 | 0.502 | 0.519 | Shorter |

### Screening for diabetic retinopathy

| Screening for diabetic retinopathy | Model | Estimate | Lower 95% CI | Upper 95% CI | Diabetes duration relative to routine monitoring window |
| --- | --- | --- | --- | --- | --- |
| Middle East | 0 | 1.207 | 1.177 | 1.238 | Longer or equal |
| Middle East | 1 | 1.101 | 1.073 | 1.130 | Longer or equal |
| Middle East | 2 | 0.943 | 0.917 | 0.970 | Longer or equal |
| Europe | 0 | 1.188 | 1.155 | 1.223 | Longer or equal |
| Europe | 1 | 1.191 | 1.158 | 1.225 | Longer or equal |
| Europe | 2 | 1.152 | 1.119 | 1.186 | Longer or equal |
| Turkey | 0 | 1.170 | 1.130 | 1.210 | Longer or equal |
| Turkey | 1 | 1.072 | 1.036 | 1.110 | Longer or equal |
| Turkey | 2 | 0.876 | 0.846 | 0.907 | Longer or equal |
| Former Yugoslavia | 0 | 1.201 | 1.157 | 1.248 | Longer or equal |
| Former Yugoslavia | 1 | 1.158 | 1.115 | 1.202 | Longer or equal |
| Former Yugoslavia | 2 | 1.049 | 1.010 | 1.088 | Longer or equal |
| Pakistan | 0 | 1.425 | 1.376 | 1.476 | Longer or equal |
| Pakistan | 1 | 1.358 | 1.311 | 1.406 | Longer or equal |
| Pakistan | 2 | 1.013 | 0.978 | 1.051 | Longer or equal |
| Sri Lanka | 0 | 0.692 | 0.636 | 0.752 | Longer or equal |
| Sri Lanka | 1 | 0.626 | 0.576 | 0.680 | Longer or equal |
| Sri Lanka | 2 | 0.656 | 0.605 | 0.712 | Longer or equal |
| Somalia | 0 | 1.360 | 1.276 | 1.448 | Longer or equal |
| Somalia | 1 | 1.118 | 1.048 | 1.193 | Longer or equal |
| Somalia | 2 | 0.978 | 0.916 | 1.045 | Longer or equal |
| Vietnam | 0 | 0.976 | 0.887 | 1.074 | Longer or equal |
| Vietnam | 1 | 0.925 | 0.841 | 1.017 | Longer or equal |
| Vietnam | 2 | 0.925 | 0.842 | 1.017 | Longer or equal |
| (Intercept) | 0 | 0.400 | 0.398 | 0.402 | Longer or equal |
| Middle East | 0 | 1.038 | 0.992 | 1.085 | Shorter |
| Middle East | 1 | 1.048 | 1.002 | 1.096 | Shorter |
| Middle East | 2 | 0.948 | 0.901 | 0.996 | Shorter |
| Europe | 0 | 1.118 | 1.074 | 1.165 | Shorter |
| Europe | 1 | 1.125 | 1.081 | 1.171 | Shorter |
| Europe | 2 | 1.083 | 1.036 | 1.132 | Shorter |
| Turkey | 0 | 1.075 | 1.016 | 1.138 | Shorter |
| Turkey | 1 | 1.097 | 1.036 | 1.161 | Shorter |
| Turkey | 2 | 0.974 | 0.920 | 1.032 | Shorter |
| Former Yugoslavia | 0 | 1.214 | 1.154 | 1.276 | Shorter |
| Former Yugoslavia | 1 | 1.231 | 1.171 | 1.294 | Shorter |
| Former Yugoslavia | 2 | 1.151 | 1.095 | 1.211 | Shorter |
| Pakistan | 0 | 1.233 | 1.152 | 1.319 | Shorter |
| Pakistan | 1 | 1.248 | 1.166 | 1.336 | Shorter |
| Pakistan | 2 | 1.048 | 0.977 | 1.125 | Shorter |
| Sri Lanka | 0 | 0.894 | 0.791 | 1.009 | Shorter |
| Sri Lanka | 1 | 0.911 | 0.806 | 1.030 | Shorter |
| Sri Lanka | 2 | 0.926 | 0.820 | 1.045 | Shorter |
| Somalia | 0 | 1.042 | 0.922 | 1.177 | Shorter |
| Somalia | 1 | 1.043 | 0.923 | 1.179 | Shorter |
| Somalia | 2 | 0.938 | 0.827 | 1.065 | Shorter |
| Vietnam | 0 | 1.035 | 0.906 | 1.183 | Shorter |
| Vietnam | 1 | 1.059 | 0.926 | 1.210 | Shorter |
| Vietnam | 2 | 1.070 | 0.937 | 1.222 | Shorter |
| (Intercept) | 0 | 0.618 | 0.612 | 0.623 | Shorter |

### Screening for diabetic foot disease

| Screening for diabetic foot disease | Model | Estimate | Lower 95% CI | Upper 95% CI | Diabetes duration relative to routine monitoring window |
| --- | --- | --- | --- | --- | --- |
| Middle East | 0 | 1.459 | 1.442 | 1.476 | Longer or equal |
| Middle East | 1 | 1.346 | 1.330 | 1.362 | Longer or equal |
| Middle East | 2 | 1.263 | 1.245 | 1.281 | Longer or equal |
| Europe | 0 | 1.148 | 1.126 | 1.170 | Longer or equal |
| Europe | 1 | 1.134 | 1.113 | 1.156 | Longer or equal |
| Europe | 2 | 1.119 | 1.097 | 1.141 | Longer or equal |
| Turkey | 0 | 1.444 | 1.422 | 1.467 | Longer or equal |
| Turkey | 1 | 1.346 | 1.324 | 1.368 | Longer or equal |
| Turkey | 2 | 1.262 | 1.241 | 1.283 | Longer or equal |
| Former Yugoslavia | 0 | 1.428 | 1.403 | 1.454 | Longer or equal |
| Former Yugoslavia | 1 | 1.372 | 1.347 | 1.397 | Longer or equal |
| Former Yugoslavia | 2 | 1.314 | 1.289 | 1.339 | Longer or equal |
| Pakistan | 0 | 1.439 | 1.411 | 1.467 | Longer or equal |
| Pakistan | 1 | 1.394 | 1.366 | 1.422 | Longer or equal |
| Pakistan | 2 | 1.267 | 1.240 | 1.294 | Longer or equal |
| Sri Lanka | 0 | 1.166 | 1.124 | 1.210 | Longer or equal |
| Sri Lanka | 1 | 1.088 | 1.049 | 1.128 | Longer or equal |
| Sri Lanka | 2 | 1.063 | 1.025 | 1.103 | Longer or equal |
| Somalia | 0 | 1.562 | 1.522 | 1.603 | Longer or equal |
| Somalia | 1 | 1.342 | 1.305 | 1.380 | Longer or equal |
| Somalia | 2 | 1.230 | 1.195 | 1.267 | Longer or equal |
| Vietnam | 0 | 1.545 | 1.500 | 1.592 | Longer or equal |
| Vietnam | 1 | 1.467 | 1.422 | 1.514 | Longer or equal |
| Vietnam | 2 | 1.403 | 1.360 | 1.448 | Longer or equal |
| (Intercept) | 0 | 0.556 | 0.553 | 0.558 | Longer or equal |
| Middle East | 0 | 1.138 | 1.102 | 1.175 | Shorter |
| Middle East | 1 | 1.133 | 1.096 | 1.171 | Shorter |
| Middle East | 2 | 1.095 | 1.050 | 1.141 | Shorter |
| Europe | 0 | 1.055 | 1.011 | 1.101 | Shorter |
| Europe | 1 | 1.055 | 1.011 | 1.101 | Shorter |
| Europe | 2 | 1.031 | 0.985 | 1.079 | Shorter |
| Turkey | 0 | 1.101 | 1.050 | 1.154 | Shorter |
| Turkey | 1 | 1.105 | 1.053 | 1.159 | Shorter |
| Turkey | 2 | 1.083 | 1.030 | 1.138 | Shorter |
| Former Yugoslavia | 0 | 1.151 | 1.103 | 1.201 | Shorter |
| Former Yugoslavia | 1 | 1.152 | 1.104 | 1.202 | Shorter |
| Former Yugoslavia | 2 | 1.125 | 1.076 | 1.176 | Shorter |
| Pakistan | 0 | 1.104 | 1.023 | 1.192 | Shorter |
| Pakistan | 1 | 1.106 | 1.025 | 1.193 | Shorter |
| Pakistan | 2 | 1.074 | 0.992 | 1.162 | Shorter |
| Sri Lanka | 0 | 1.105 | 1.022 | 1.196 | Shorter |
| Sri Lanka | 1 | 1.110 | 1.027 | 1.201 | Shorter |
| Sri Lanka | 2 | 1.100 | 1.017 | 1.190 | Shorter |
| Somalia | 0 | 1.210 | 1.145 | 1.278 | Shorter |
| Somalia | 1 | 1.199 | 1.133 | 1.268 | Shorter |
| Somalia | 2 | 1.144 | 1.077 | 1.216 | Shorter |
| Vietnam | 0 | 1.150 | 1.053 | 1.254 | Shorter |
| Vietnam | 1 | 1.162 | 1.064 | 1.268 | Shorter |
| Vietnam | 2 | 1.130 | 1.034 | 1.234 | Shorter |
| (Intercept) | 0 | 0.788 | 0.781 | 0.795 | Shorter |
