# Supplementary material for: Guideline-level monitoring, biomarker levels and pharmacological treatment in migrants and native Danes with type 2 diabetes: Population-wide analyses
Source: PLOS Glob Public Health. 2023 Oct 18;3(10):e0001277. doi: 10.1371/journal.pgph.0001277 (PMC10584163; doi:10.1371/journal.pgph.0001277)
Supplement: S5 File — (HTML) [file pgph.0001277.s005.html]

S5: Regression coefficients of relative risk from all analyses.


# S5: Regression coefficients of relative risk from all analyses.

- S5: Regression coefficients from analyses.
  - Prevalence: Analysis of prevalent type 2 diabetes
  - Monitoring: Analysis of HbA1c monitoring
  - Monitoring: Analysis of LDL-C monitoring
  - Monitoring: Analysis of screening for diabetic nephropathy
  - Monitoring: Analysis of screening for diabetic retinopathy
  - Monitoring: Analysis of screening for diabetic foot disease
  - Biomarker levels: Analysis of HbA1c levels
  - Biomarker levels: Analysis of LDL-C levels
  - Pharmacological treatment: Analysis of glucose-lowering drugs
  - Pharmacological treatment: Analysis of lipid-lowering drugs
  - Pharmacological treatment: Analysis of ACEI/ARB
  - Pharmacological treatment: Analysis of antiplatelet therapy

# S5: Regression coefficients from analyses.

### Prevalence: Analysis of prevalent type 2 diabetes

| Prevalence: Analysis of prevalent type 2 diabetes | Model | Estimate | Lower 95% CI | Upper 95% CI | P-value |
| --- | --- | --- | --- | --- | --- |
| Native Danes (reference risk) | 0 | 0.066 | 0.066 | 0.066 | 0 |
| originMiddle East | 0 | 1.665 | 1.631 | 1.700 | 0 |
| originEurope | 0 | 0.625 | 0.610 | 0.640 | 0 |
| originTurkey | 0 | 2.310 | 2.249 | 2.373 | 0 |
| originFormer Yugoslavia | 0 | 1.681 | 1.630 | 1.734 | 0 |
| originPakistan | 0 | 3.628 | 3.512 | 3.749 | 0 |
| originSri Lanka | 0 | 3.976 | 3.817 | 4.143 | 0 |
| originSomalia | 0 | 1.655 | 1.560 | 1.755 | 0 |
| originVietnam | 0 | 1.421 | 1.331 | 1.518 | 0 |

| Prevalence: Analysis of prevalent type 2 diabetes | Model | Estimate | Lower 95% CI | Upper 95% CI | P-value |
| --- | --- | --- | --- | --- | --- |
| (Intercept) | 1 | 0.043 | 0.042 | 0.043 | 0 |
| originMiddle East | 1 | 2.759 | 2.705 | 2.813 | 0 |
| originEurope | 1 | 0.941 | 0.920 | 0.963 | 0 |
| originTurkey | 1 | 3.543 | 3.455 | 3.633 | 0 |
| originFormer Yugoslavia | 1 | 2.372 | 2.305 | 2.442 | 0 |
| originPakistan | 1 | 5.138 | 4.992 | 5.288 | 0 |
| originSri Lanka | 1 | 5.616 | 5.407 | 5.833 | 0 |
| originSomalia | 1 | 3.725 | 3.520 | 3.941 | 0 |
| originVietnam | 1 | 2.100 | 1.975 | 2.232 | 0 |
| sexM | 1 | 1.389 | 1.379 | 1.399 | 0 |
| Ns(Age)1 | 1 | 7.492 | 7.372 | 7.614 | 0 |
| Ns(Age)2 | 1 | 34.619 | 33.483 | 35.793 | 0 |
| Ns(Age)3 | 1 | 7.183 | 7.085 | 7.282 | 0 |

| Prevalence: Analysis of prevalent type 2 diabetes | Model | Estimate | Lower 95% CI | Upper 95% CI | P-value |
| --- | --- | --- | --- | --- | --- |
| (Intercept) | 2 | 0.027 | 0.026 | 0.029 | 0.000 |
| originMiddle East | 2 | 1.818 | 1.778 | 1.858 | 0.000 |
| originEurope | 2 | 0.965 | 0.942 | 0.988 | 0.004 |
| originTurkey | 2 | 2.367 | 2.307 | 2.429 | 0.000 |
| originFormer Yugoslavia | 2 | 1.632 | 1.584 | 1.681 | 0.000 |
| originPakistan | 2 | 3.812 | 3.700 | 3.928 | 0.000 |
| originSri Lanka | 2 | 4.041 | 3.889 | 4.198 | 0.000 |
| originSomalia | 2 | 2.206 | 2.083 | 2.337 | 0.000 |
| originVietnam | 2 | 1.584 | 1.489 | 1.685 | 0.000 |
| sexM | 2 | 1.464 | 1.453 | 1.474 | 0.000 |
| Ns(Age)1 | 2 | 7.056 | 6.930 | 7.185 | 0.000 |
| Ns(Age)2 | 2 | 33.157 | 32.029 | 34.325 | 0.000 |
| Ns(Age)3 | 2 | 5.762 | 5.660 | 5.866 | 0.000 |
| employment\_statusRetired | 2 | 1.331 | 1.313 | 1.350 | 0.000 |
| employment\_statusUnemployed | 2 | 1.934 | 1.909 | 1.959 | 0.000 |
| Ns(household\_income)1 | 2 | 0.721 | 0.710 | 0.732 | 0.000 |
| Ns(household\_income)2 | 2 | 0.585 | 0.578 | 0.592 | 0.000 |
| Ns(household\_income)3 | 2 | 0.580 | 0.573 | 0.587 | 0.000 |
| duration\_of\_residence[10,15) | 2 | 1.251 | 1.160 | 1.349 | 0.000 |
| duration\_of\_residence[15,20) | 2 | 1.371 | 1.293 | 1.454 | 0.000 |
| duration\_of\_residence[20,Inf) | 2 | 1.504 | 1.429 | 1.583 | 0.000 |
| regionCentral Denmark Region | 2 | 0.972 | 0.962 | 0.982 | 0.000 |
| regionNorth Denmark Region | 2 | 0.974 | 0.962 | 0.987 | 0.000 |
| regionSouth Denmark Region | 2 | 0.955 | 0.945 | 0.965 | 0.000 |
| regionZealand Region | 2 | 1.052 | 1.040 | 1.064 | 0.000 |

### Monitoring: Analysis of HbA1c monitoring

| Monitoring: Analysis of HbA1c monitoring | Model | Estimate | Lower 95% CI | Upper 95% CI | P-value |
| --- | --- | --- | --- | --- | --- |
| Native Danes (reference risk) | 0 | 0.068 | 0.067 | 0.069 | 0.000 |
| originMiddle East | 0 | 1.175 | 1.088 | 1.268 | 0.000 |
| originEurope | 0 | 1.320 | 1.220 | 1.430 | 0.000 |
| originTurkey | 0 | 0.980 | 0.878 | 1.095 | 0.725 |
| originFormer Yugoslavia | 0 | 0.957 | 0.844 | 1.086 | 0.494 |
| originPakistan | 0 | 1.118 | 0.980 | 1.275 | 0.097 |
| originSri Lanka | 0 | 0.639 | 0.509 | 0.801 | 0.000 |
| originSomalia | 0 | 1.624 | 1.356 | 1.945 | 0.000 |
| originVietnam | 0 | 1.391 | 1.119 | 1.729 | 0.003 |

| Monitoring: Analysis of HbA1c monitoring | Model | Estimate | Lower 95% CI | Upper 95% CI | P-value |
| --- | --- | --- | --- | --- | --- |
| (Intercept) | 1 | 0.070 | 0.067 | 0.072 | 0.000 |
| originMiddle East | 1 | 0.966 | 0.895 | 1.044 | 0.382 |
| originEurope | 1 | 1.333 | 1.233 | 1.441 | 0.000 |
| originTurkey | 1 | 0.797 | 0.714 | 0.891 | 0.000 |
| originFormer Yugoslavia | 1 | 0.868 | 0.765 | 0.984 | 0.027 |
| originPakistan | 1 | 0.958 | 0.839 | 1.094 | 0.526 |
| originSri Lanka | 1 | 0.496 | 0.395 | 0.623 | 0.000 |
| originSomalia | 1 | 1.083 | 0.902 | 1.300 | 0.392 |
| originVietnam | 1 | 1.257 | 1.013 | 1.559 | 0.038 |
| sexM | 1 | 0.954 | 0.927 | 0.982 | 0.001 |
| Ns(age)1 | 1 | 0.445 | 0.422 | 0.470 | 0.000 |
| Ns(age)2 | 1 | 0.412 | 0.396 | 0.429 | 0.000 |
| Ns(age)3 | 1 | 0.622 | 0.597 | 0.649 | 0.000 |
| Ns(diabetes\_duration)1 | 1 | 1.016 | 0.965 | 1.069 | 0.543 |
| Ns(diabetes\_duration)2 | 1 | 1.671 | 1.591 | 1.756 | 0.000 |
| Ns(diabetes\_duration)3 | 1 | 0.896 | 0.860 | 0.932 | 0.000 |
| macrovasc\_compTRUE | 1 | 0.828 | 0.799 | 0.858 | 0.000 |
| dkdTRUE | 1 | 0.263 | 0.231 | 0.298 | 0.000 |

| Monitoring: Analysis of HbA1c monitoring | Model | Estimate | Lower 95% CI | Upper 95% CI | P-value |
| --- | --- | --- | --- | --- | --- |
| (Intercept) | 2 | 0.101 | 0.085 | 0.120 | 0.000 |
| originMiddle East | 2 | 0.901 | 0.826 | 0.984 | 0.020 |
| originEurope | 2 | 1.271 | 1.171 | 1.379 | 0.000 |
| originTurkey | 2 | 0.674 | 0.602 | 0.754 | 0.000 |
| originFormer Yugoslavia | 2 | 0.838 | 0.738 | 0.952 | 0.006 |
| originPakistan | 2 | 0.694 | 0.605 | 0.795 | 0.000 |
| originSri Lanka | 2 | 0.551 | 0.439 | 0.691 | 0.000 |
| originSomalia | 2 | 1.039 | 0.860 | 1.256 | 0.691 |
| originVietnam | 2 | 1.307 | 1.053 | 1.621 | 0.015 |
| sexM | 2 | 0.930 | 0.903 | 0.957 | 0.000 |
| Ns(age)1 | 2 | 0.480 | 0.441 | 0.524 | 0.000 |
| Ns(age)2 | 2 | 0.449 | 0.415 | 0.486 | 0.000 |
| Ns(age)3 | 2 | 0.657 | 0.610 | 0.709 | 0.000 |
| Ns(diabetes\_duration)1 | 2 | 1.025 | 0.974 | 1.079 | 0.335 |
| Ns(diabetes\_duration)2 | 2 | 1.667 | 1.586 | 1.752 | 0.000 |
| Ns(diabetes\_duration)3 | 2 | 0.903 | 0.868 | 0.940 | 0.000 |
| macrovasc\_compTRUE | 2 | 0.845 | 0.815 | 0.875 | 0.000 |
| dkdTRUE | 2 | 0.267 | 0.235 | 0.303 | 0.000 |
| employment\_statusRetired | 2 | 0.770 | 0.720 | 0.824 | 0.000 |
| employment\_statusUnemployed | 2 | 0.669 | 0.639 | 0.701 | 0.000 |
| Ns(household\_income)1 | 2 | 0.813 | 0.767 | 0.861 | 0.000 |
| Ns(household\_income)2 | 2 | 0.728 | 0.695 | 0.762 | 0.000 |
| Ns(household\_income)3 | 2 | 0.895 | 0.855 | 0.937 | 0.000 |
| duration\_of\_residence[10,15) | 2 | 0.840 | 0.651 | 1.085 | 0.182 |
| duration\_of\_residence[15,20) | 2 | 0.949 | 0.784 | 1.148 | 0.591 |
| duration\_of\_residence[20,Inf) | 2 | 1.049 | 0.897 | 1.226 | 0.549 |
| regionCentral Denmark Region | 2 | 0.699 | 0.672 | 0.728 | 0.000 |
| regionNorth Denmark Region | 2 | 0.609 | 0.576 | 0.643 | 0.000 |
| regionSouth Denmark Region | 2 | 0.619 | 0.593 | 0.645 | 0.000 |
| regionZealand Region | 2 | 0.849 | 0.815 | 0.885 | 0.000 |

### Monitoring: Analysis of LDL-C monitoring

| Monitoring: Analysis of LDL-C monitoring | Model | Estimate | Lower 95% CI | Upper 95% CI | P-value |
| --- | --- | --- | --- | --- | --- |
| Native Danes (reference risk) | 0 | 0.131 | 0.130 | 0.133 | 0.000 |
| originMiddle East | 0 | 1.167 | 1.107 | 1.230 | 0.000 |
| originEurope | 0 | 1.176 | 1.110 | 1.247 | 0.000 |
| originTurkey | 0 | 1.176 | 1.097 | 1.261 | 0.000 |
| originFormer Yugoslavia | 0 | 0.947 | 0.867 | 1.035 | 0.228 |
| originPakistan | 0 | 1.207 | 1.106 | 1.317 | 0.000 |
| originSri Lanka | 0 | 0.705 | 0.606 | 0.821 | 0.000 |
| originSomalia | 0 | 1.402 | 1.226 | 1.603 | 0.000 |
| originVietnam | 0 | 0.958 | 0.796 | 1.153 | 0.647 |

| Monitoring: Analysis of LDL-C monitoring | Model | Estimate | Lower 95% CI | Upper 95% CI | P-value |
| --- | --- | --- | --- | --- | --- |
| (Intercept) | 1 | 0.111 | 0.109 | 0.114 | 0.000 |
| originMiddle East | 1 | 1.024 | 0.971 | 1.080 | 0.387 |
| originEurope | 1 | 1.189 | 1.123 | 1.260 | 0.000 |
| originTurkey | 1 | 1.032 | 0.963 | 1.107 | 0.370 |
| originFormer Yugoslavia | 1 | 0.900 | 0.824 | 0.984 | 0.020 |
| originPakistan | 1 | 1.111 | 1.018 | 1.213 | 0.018 |
| originSri Lanka | 1 | 0.589 | 0.507 | 0.685 | 0.000 |
| originSomalia | 1 | 1.031 | 0.901 | 1.179 | 0.661 |
| originVietnam | 1 | 0.882 | 0.733 | 1.060 | 0.181 |
| sexM | 1 | 0.974 | 0.954 | 0.993 | 0.009 |
| Ns(age)1 | 1 | 0.493 | 0.475 | 0.511 | 0.000 |
| Ns(age)2 | 1 | 0.493 | 0.480 | 0.507 | 0.000 |
| Ns(age)3 | 1 | 0.750 | 0.729 | 0.773 | 0.000 |
| Ns(diabetes\_duration)1 | 1 | 1.028 | 0.993 | 1.065 | 0.123 |
| Ns(diabetes\_duration)2 | 1 | 1.289 | 1.247 | 1.334 | 0.000 |
| Ns(diabetes\_duration)3 | 1 | 0.984 | 0.957 | 1.011 | 0.245 |
| macrovasc\_compTRUE | 1 | 0.862 | 0.842 | 0.883 | 0.000 |
| dkdTRUE | 1 | 0.741 | 0.703 | 0.781 | 0.000 |

| Monitoring: Analysis of LDL-C monitoring | Model | Estimate | Lower 95% CI | Upper 95% CI | P-value |
| --- | --- | --- | --- | --- | --- |
| (Intercept) | 2 | 0.169 | 0.149 | 0.191 | 0.000 |
| originMiddle East | 2 | 0.898 | 0.846 | 0.953 | 0.000 |
| originEurope | 2 | 1.127 | 1.061 | 1.197 | 0.000 |
| originTurkey | 2 | 0.832 | 0.775 | 0.893 | 0.000 |
| originFormer Yugoslavia | 2 | 0.836 | 0.765 | 0.914 | 0.000 |
| originPakistan | 2 | 0.779 | 0.712 | 0.852 | 0.000 |
| originSri Lanka | 2 | 0.629 | 0.541 | 0.731 | 0.000 |
| originSomalia | 2 | 0.932 | 0.812 | 1.068 | 0.311 |
| originVietnam | 2 | 0.904 | 0.753 | 1.086 | 0.280 |
| sexM | 2 | 0.970 | 0.950 | 0.989 | 0.003 |
| Ns(age)1 | 2 | 0.511 | 0.481 | 0.543 | 0.000 |
| Ns(age)2 | 2 | 0.518 | 0.490 | 0.548 | 0.000 |
| Ns(age)3 | 2 | 0.765 | 0.725 | 0.806 | 0.000 |
| Ns(diabetes\_duration)1 | 2 | 1.041 | 1.005 | 1.079 | 0.024 |
| Ns(diabetes\_duration)2 | 2 | 1.266 | 1.224 | 1.309 | 0.000 |
| Ns(diabetes\_duration)3 | 2 | 0.996 | 0.969 | 1.024 | 0.764 |
| macrovasc\_compTRUE | 2 | 0.864 | 0.844 | 0.885 | 0.000 |
| dkdTRUE | 2 | 0.743 | 0.705 | 0.783 | 0.000 |
| employment\_statusRetired | 2 | 0.841 | 0.802 | 0.882 | 0.000 |
| employment\_statusUnemployed | 2 | 0.811 | 0.786 | 0.837 | 0.000 |
| Ns(household\_income)1 | 2 | 0.855 | 0.821 | 0.889 | 0.000 |
| Ns(household\_income)2 | 2 | 0.780 | 0.756 | 0.805 | 0.000 |
| Ns(household\_income)3 | 2 | 0.873 | 0.846 | 0.900 | 0.000 |
| duration\_of\_residence[10,15) | 2 | 0.938 | 0.786 | 1.120 | 0.481 |
| duration\_of\_residence[15,20) | 2 | 0.883 | 0.769 | 1.014 | 0.077 |
| duration\_of\_residence[20,Inf) | 2 | 1.003 | 0.896 | 1.123 | 0.958 |
| regionCentral Denmark Region | 2 | 0.742 | 0.723 | 0.762 | 0.000 |
| regionNorth Denmark Region | 2 | 0.670 | 0.646 | 0.694 | 0.000 |
| regionSouth Denmark Region | 2 | 0.481 | 0.466 | 0.497 | 0.000 |
| regionZealand Region | 2 | 0.752 | 0.730 | 0.774 | 0.000 |

### Monitoring: Analysis of screening for diabetic nephropathy

| Monitoring: Analysis of screening for diabetic nephropathy | Model | Estimate | Lower 95% CI | Upper 95% CI | P-value |
| --- | --- | --- | --- | --- | --- |
| Native Danes (reference risk) | 0 | 0.436 | 0.434 | 0.438 | 0.000 |
| originMiddle East | 0 | 1.110 | 1.085 | 1.137 | 0.000 |
| originEurope | 0 | 1.138 | 1.110 | 1.167 | 0.000 |
| originTurkey | 0 | 1.069 | 1.036 | 1.103 | 0.000 |
| originFormer Yugoslavia | 0 | 1.035 | 0.998 | 1.074 | 0.067 |
| originPakistan | 0 | 1.130 | 1.087 | 1.174 | 0.000 |
| originSri Lanka | 0 | 0.789 | 0.738 | 0.844 | 0.000 |
| originSomalia | 0 | 1.291 | 1.221 | 1.366 | 0.000 |
| originVietnam | 0 | 0.944 | 0.868 | 1.026 | 0.176 |

| Monitoring: Analysis of screening for diabetic nephropathy | Model | Estimate | Lower 95% CI | Upper 95% CI | P-value |
| --- | --- | --- | --- | --- | --- |
| (Intercept) | 1 | 0.428 | 0.424 | 0.433 | 0.000 |
| originMiddle East | 1 | 1.107 | 1.081 | 1.133 | 0.000 |
| originEurope | 1 | 1.120 | 1.093 | 1.148 | 0.000 |
| originTurkey | 1 | 1.052 | 1.019 | 1.085 | 0.002 |
| originFormer Yugoslavia | 1 | 1.036 | 0.999 | 1.074 | 0.056 |
| originPakistan | 1 | 1.143 | 1.101 | 1.187 | 0.000 |
| originSri Lanka | 1 | 0.793 | 0.742 | 0.846 | 0.000 |
| originSomalia | 1 | 1.211 | 1.144 | 1.281 | 0.000 |
| originVietnam | 1 | 0.957 | 0.881 | 1.039 | 0.293 |
| sexM | 1 | 0.872 | 0.865 | 0.880 | 0.000 |
| Ns(age)1 | 1 | 0.848 | 0.835 | 0.861 | 0.000 |
| Ns(age)2 | 1 | 0.876 | 0.866 | 0.886 | 0.000 |
| Ns(age)3 | 1 | 1.004 | 0.991 | 1.016 | 0.559 |
| Ns(diabetes\_duration)1 | 1 | 0.939 | 0.924 | 0.954 | 0.000 |
| Ns(diabetes\_duration)2 | 1 | 0.821 | 0.810 | 0.832 | 0.000 |
| Ns(diabetes\_duration)3 | 1 | 0.919 | 0.908 | 0.931 | 0.000 |
| macrovasc\_compTRUE | 1 | 1.032 | 1.022 | 1.042 | 0.000 |
| dkdTRUE | 1 | 0.223 | 0.212 | 0.234 | 0.000 |

| Monitoring: Analysis of screening for diabetic nephropathy | Model | Estimate | Lower 95% CI | Upper 95% CI | P-value |
| --- | --- | --- | --- | --- | --- |
| (Intercept) | 2 | 0.502 | 0.473 | 0.532 | 0.000 |
| originMiddle East | 2 | 1.023 | 0.996 | 1.050 | 0.093 |
| originEurope | 2 | 1.084 | 1.057 | 1.112 | 0.000 |
| originTurkey | 2 | 0.936 | 0.907 | 0.967 | 0.000 |
| originFormer Yugoslavia | 2 | 0.969 | 0.935 | 1.005 | 0.092 |
| originPakistan | 2 | 0.935 | 0.899 | 0.972 | 0.001 |
| originSri Lanka | 2 | 0.886 | 0.831 | 0.945 | 0.000 |
| originSomalia | 2 | 1.165 | 1.099 | 1.236 | 0.000 |
| originVietnam | 2 | 0.993 | 0.915 | 1.077 | 0.860 |
| sexM | 2 | 0.877 | 0.870 | 0.885 | 0.000 |
| Ns(age)1 | 2 | 0.865 | 0.844 | 0.886 | 0.000 |
| Ns(age)2 | 2 | 0.893 | 0.872 | 0.913 | 0.000 |
| Ns(age)3 | 2 | 1.020 | 0.999 | 1.043 | 0.067 |
| Ns(diabetes\_duration)1 | 2 | 0.936 | 0.921 | 0.951 | 0.000 |
| Ns(diabetes\_duration)2 | 2 | 0.811 | 0.800 | 0.822 | 0.000 |
| Ns(diabetes\_duration)3 | 2 | 0.913 | 0.902 | 0.924 | 0.000 |
| macrovasc\_compTRUE | 2 | 1.032 | 1.022 | 1.042 | 0.000 |
| dkdTRUE | 2 | 0.222 | 0.211 | 0.233 | 0.000 |
| employment\_statusRetired | 2 | 0.983 | 0.963 | 1.003 | 0.088 |
| employment\_statusUnemployed | 2 | 1.044 | 1.029 | 1.059 | 0.000 |
| Ns(household\_income)1 | 2 | 0.936 | 0.921 | 0.952 | 0.000 |
| Ns(household\_income)2 | 2 | 0.938 | 0.925 | 0.951 | 0.000 |
| Ns(household\_income)3 | 2 | 0.956 | 0.943 | 0.968 | 0.000 |
| duration\_of\_residence[10,15) | 2 | 1.043 | 0.962 | 1.131 | 0.304 |
| duration\_of\_residence[15,20) | 2 | 1.032 | 0.969 | 1.100 | 0.322 |
| duration\_of\_residence[20,Inf) | 2 | 1.042 | 0.988 | 1.100 | 0.132 |
| regionCentral Denmark Region | 2 | 0.575 | 0.567 | 0.583 | 0.000 |
| regionNorth Denmark Region | 2 | 0.962 | 0.950 | 0.975 | 0.000 |
| regionSouth Denmark Region | 2 | 0.738 | 0.730 | 0.748 | 0.000 |
| regionZealand Region | 2 | 0.886 | 0.876 | 0.897 | 0.000 |

### Monitoring: Analysis of screening for diabetic retinopathy

| Monitoring: Analysis of screening for diabetic retinopathy | Model | Estimate | Lower 95% CI | Upper 95% CI | P-value |
| --- | --- | --- | --- | --- | --- |
| Native Danes (reference risk) | 0 | 0.432 | 0.430 | 0.434 | 0.00 |
| originMiddle East | 0 | 1.171 | 1.145 | 1.197 | 0.00 |
| originEurope | 0 | 1.185 | 1.157 | 1.215 | 0.00 |
| originTurkey | 0 | 1.147 | 1.113 | 1.182 | 0.00 |
| originFormer Yugoslavia | 0 | 1.206 | 1.168 | 1.245 | 0.00 |
| originPakistan | 0 | 1.364 | 1.322 | 1.408 | 0.00 |
| originSri Lanka | 0 | 0.723 | 0.673 | 0.776 | 0.00 |
| originSomalia | 0 | 1.294 | 1.223 | 1.369 | 0.00 |
| originVietnam | 0 | 0.994 | 0.917 | 1.078 | 0.89 |

| Monitoring: Analysis of screening for diabetic retinopathy | Model | Estimate | Lower 95% CI | Upper 95% CI | P-value |
| --- | --- | --- | --- | --- | --- |
| (Intercept) | 1 | 0.306 | 0.303 | 0.309 | 0.000 |
| originMiddle East | 1 | 1.092 | 1.067 | 1.116 | 0.000 |
| originEurope | 1 | 1.170 | 1.143 | 1.198 | 0.000 |
| originTurkey | 1 | 1.080 | 1.049 | 1.113 | 0.000 |
| originFormer Yugoslavia | 1 | 1.169 | 1.133 | 1.207 | 0.000 |
| originPakistan | 1 | 1.350 | 1.308 | 1.393 | 0.000 |
| originSri Lanka | 1 | 0.678 | 0.632 | 0.728 | 0.000 |
| originSomalia | 1 | 1.114 | 1.052 | 1.180 | 0.000 |
| originVietnam | 1 | 0.951 | 0.878 | 1.029 | 0.210 |
| sexM | 1 | 1.022 | 1.013 | 1.031 | 0.000 |
| Ns(age)1 | 1 | 0.670 | 0.659 | 0.680 | 0.000 |
| Ns(age)2 | 1 | 0.688 | 0.680 | 0.696 | 0.000 |
| Ns(age)3 | 1 | 0.872 | 0.861 | 0.883 | 0.000 |
| Ns(diabetes\_duration)1 | 1 | 0.802 | 0.789 | 0.816 | 0.000 |
| Ns(diabetes\_duration)2 | 1 | 0.581 | 0.573 | 0.588 | 0.000 |
| Ns(diabetes\_duration)3 | 1 | 0.849 | 0.838 | 0.860 | 0.000 |
| macrovasc\_compTRUE | 1 | 1.131 | 1.120 | 1.142 | 0.000 |
| dkdTRUE | 1 | 1.005 | 0.985 | 1.025 | 0.642 |

| Monitoring: Analysis of screening for diabetic retinopathy | Model | Estimate | Lower 95% CI | Upper 95% CI | P-value |
| --- | --- | --- | --- | --- | --- |
| (Intercept) | 2 | 0.363 | 0.345 | 0.382 | 0.000 |
| originMiddle East | 2 | 0.951 | 0.928 | 0.975 | 0.000 |
| originEurope | 2 | 1.134 | 1.106 | 1.162 | 0.000 |
| originTurkey | 2 | 0.900 | 0.873 | 0.927 | 0.000 |
| originFormer Yugoslavia | 2 | 1.068 | 1.035 | 1.102 | 0.000 |
| originPakistan | 2 | 1.036 | 1.002 | 1.070 | 0.036 |
| originSri Lanka | 2 | 0.707 | 0.659 | 0.757 | 0.000 |
| originSomalia | 2 | 0.983 | 0.927 | 1.042 | 0.567 |
| originVietnam | 2 | 0.956 | 0.884 | 1.035 | 0.266 |
| sexM | 2 | 1.029 | 1.020 | 1.038 | 0.000 |
| Ns(age)1 | 2 | 0.738 | 0.719 | 0.757 | 0.000 |
| Ns(age)2 | 2 | 0.764 | 0.746 | 0.782 | 0.000 |
| Ns(age)3 | 2 | 0.944 | 0.922 | 0.965 | 0.000 |
| Ns(diabetes\_duration)1 | 2 | 0.805 | 0.792 | 0.818 | 0.000 |
| Ns(diabetes\_duration)2 | 2 | 0.570 | 0.562 | 0.577 | 0.000 |
| Ns(diabetes\_duration)3 | 2 | 0.851 | 0.840 | 0.862 | 0.000 |
| macrovasc\_compTRUE | 2 | 1.124 | 1.113 | 1.135 | 0.000 |
| dkdTRUE | 2 | 0.997 | 0.978 | 1.017 | 0.802 |
| employment\_statusRetired | 2 | 0.885 | 0.867 | 0.904 | 0.000 |
| employment\_statusUnemployed | 2 | 1.010 | 0.997 | 1.023 | 0.148 |
| Ns(household\_income)1 | 2 | 0.873 | 0.858 | 0.888 | 0.000 |
| Ns(household\_income)2 | 2 | 0.826 | 0.816 | 0.837 | 0.000 |
| Ns(household\_income)3 | 2 | 0.911 | 0.899 | 0.923 | 0.000 |
| duration\_of\_residence[10,15) | 2 | 1.099 | 1.024 | 1.179 | 0.009 |
| duration\_of\_residence[15,20) | 2 | 1.052 | 0.995 | 1.112 | 0.076 |
| duration\_of\_residence[20,Inf) | 2 | 1.110 | 1.060 | 1.162 | 0.000 |
| regionCentral Denmark Region | 2 | 0.749 | 0.740 | 0.758 | 0.000 |
| regionNorth Denmark Region | 2 | 0.777 | 0.765 | 0.789 | 0.000 |
| regionSouth Denmark Region | 2 | 0.615 | 0.607 | 0.623 | 0.000 |
| regionZealand Region | 2 | 0.850 | 0.840 | 0.861 | 0.000 |

### Monitoring: Analysis of screening for diabetic foot disease

| Monitoring: Analysis of screening for diabetic foot disease | Model | Estimate | Lower 95% CI | Upper 95% CI | P-value |
| --- | --- | --- | --- | --- | --- |
| Native Danes (reference risk) | 0 | 0.570 | 0.568 | 0.572 | 0 |
| originMiddle East | 0 | 1.432 | 1.416 | 1.448 | 0 |
| originEurope | 0 | 1.144 | 1.123 | 1.165 | 0 |
| originTurkey | 0 | 1.416 | 1.395 | 1.437 | 0 |
| originFormer Yugoslavia | 0 | 1.406 | 1.382 | 1.430 | 0 |
| originPakistan | 0 | 1.408 | 1.381 | 1.435 | 0 |
| originSri Lanka | 0 | 1.160 | 1.120 | 1.201 | 0 |
| originSomalia | 0 | 1.534 | 1.497 | 1.572 | 0 |
| originVietnam | 0 | 1.513 | 1.470 | 1.556 | 0 |

| Monitoring: Analysis of screening for diabetic foot disease | Model | Estimate | Lower 95% CI | Upper 95% CI | P-value |
| --- | --- | --- | --- | --- | --- |
| (Intercept) | 1 | 0.505 | 0.501 | 0.509 | 0.000 |
| originMiddle East | 1 | 1.330 | 1.315 | 1.345 | 0.000 |
| originEurope | 1 | 1.125 | 1.106 | 1.145 | 0.000 |
| originTurkey | 1 | 1.328 | 1.307 | 1.348 | 0.000 |
| originFormer Yugoslavia | 1 | 1.351 | 1.328 | 1.375 | 0.000 |
| originPakistan | 1 | 1.381 | 1.354 | 1.408 | 0.000 |
| originSri Lanka | 1 | 1.091 | 1.055 | 1.129 | 0.000 |
| originSomalia | 1 | 1.331 | 1.296 | 1.366 | 0.000 |
| originVietnam | 1 | 1.439 | 1.396 | 1.483 | 0.000 |
| sexM | 1 | 1.077 | 1.070 | 1.084 | 0.000 |
| Ns(age)1 | 1 | 0.796 | 0.787 | 0.805 | 0.000 |
| Ns(age)2 | 1 | 0.769 | 0.763 | 0.775 | 0.000 |
| Ns(age)3 | 1 | 0.858 | 0.851 | 0.866 | 0.000 |
| Ns(diabetes\_duration)1 | 1 | 0.923 | 0.911 | 0.934 | 0.000 |
| Ns(diabetes\_duration)2 | 1 | 0.694 | 0.688 | 0.701 | 0.000 |
| Ns(diabetes\_duration)3 | 1 | 0.840 | 0.832 | 0.848 | 0.000 |
| macrovasc\_compTRUE | 1 | 0.994 | 0.986 | 1.001 | 0.092 |
| dkdTRUE | 1 | 0.914 | 0.899 | 0.930 | 0.000 |

| Monitoring: Analysis of screening for diabetic foot disease | Model | Estimate | Lower 95% CI | Upper 95% CI | P-value |
| --- | --- | --- | --- | --- | --- |
| (Intercept) | 2 | 0.524 | 0.509 | 0.539 | 0.000 |
| originMiddle East | 2 | 1.253 | 1.236 | 1.270 | 0.000 |
| originEurope | 2 | 1.111 | 1.090 | 1.131 | 0.000 |
| originTurkey | 2 | 1.250 | 1.230 | 1.270 | 0.000 |
| originFormer Yugoslavia | 2 | 1.298 | 1.275 | 1.321 | 0.000 |
| originPakistan | 2 | 1.264 | 1.238 | 1.291 | 0.000 |
| originSri Lanka | 2 | 1.067 | 1.031 | 1.104 | 0.000 |
| originSomalia | 2 | 1.225 | 1.192 | 1.260 | 0.000 |
| originVietnam | 2 | 1.378 | 1.338 | 1.420 | 0.000 |
| sexM | 2 | 1.075 | 1.068 | 1.082 | 0.000 |
| Ns(age)1 | 2 | 0.892 | 0.875 | 0.908 | 0.000 |
| Ns(age)2 | 2 | 0.868 | 0.854 | 0.883 | 0.000 |
| Ns(age)3 | 2 | 0.940 | 0.925 | 0.956 | 0.000 |
| Ns(diabetes\_duration)1 | 2 | 0.923 | 0.912 | 0.934 | 0.000 |
| Ns(diabetes\_duration)2 | 2 | 0.693 | 0.687 | 0.700 | 0.000 |
| Ns(diabetes\_duration)3 | 2 | 0.841 | 0.833 | 0.849 | 0.000 |
| macrovasc\_compTRUE | 2 | 0.994 | 0.987 | 1.002 | 0.131 |
| dkdTRUE | 2 | 0.915 | 0.900 | 0.930 | 0.000 |
| employment\_statusRetired | 2 | 0.859 | 0.847 | 0.872 | 0.000 |
| employment\_statusUnemployed | 2 | 0.947 | 0.938 | 0.956 | 0.000 |
| Ns(household\_income)1 | 2 | 0.923 | 0.912 | 0.935 | 0.000 |
| Ns(household\_income)2 | 2 | 0.859 | 0.851 | 0.867 | 0.000 |
| Ns(household\_income)3 | 2 | 0.950 | 0.941 | 0.960 | 0.000 |
| duration\_of\_residence[10,15) | 2 | 1.087 | 1.049 | 1.125 | 0.000 |
| duration\_of\_residence[15,20) | 2 | 1.080 | 1.052 | 1.110 | 0.000 |
| duration\_of\_residence[20,Inf) | 2 | 1.090 | 1.065 | 1.115 | 0.000 |
| regionCentral Denmark Region | 2 | 0.977 | 0.968 | 0.985 | 0.000 |
| regionNorth Denmark Region | 2 | 0.971 | 0.960 | 0.982 | 0.000 |
| regionSouth Denmark Region | 2 | 0.949 | 0.941 | 0.958 | 0.000 |
| regionZealand Region | 2 | 0.932 | 0.923 | 0.942 | 0.000 |

### Biomarker levels: Analysis of HbA1c levels

| Biomarker levels: Analysis of HbA1c levels | Model | Estimate | Lower 95% CI | Upper 95% CI | P-value |
| --- | --- | --- | --- | --- | --- |
| Native Danes (reference risk) | 0 | 0.372 | 0.370 | 0.374 | 0.000 |
| originMiddle East | 0 | 1.269 | 1.239 | 1.300 | 0.000 |
| originEurope | 0 | 1.026 | 0.993 | 1.059 | 0.123 |
| originTurkey | 0 | 1.442 | 1.402 | 1.483 | 0.000 |
| originFormer Yugoslavia | 0 | 1.326 | 1.281 | 1.372 | 0.000 |
| originPakistan | 0 | 1.464 | 1.413 | 1.516 | 0.000 |
| originSri Lanka | 0 | 1.381 | 1.317 | 1.449 | 0.000 |
| originSomalia | 0 | 1.333 | 1.249 | 1.422 | 0.000 |
| originVietnam | 0 | 0.979 | 0.890 | 1.076 | 0.656 |

| Biomarker levels: Analysis of HbA1c levels | Model | Estimate | Lower 95% CI | Upper 95% CI | P-value |
| --- | --- | --- | --- | --- | --- |
| (Intercept) | 1 | 0.288 | 0.285 | 0.292 | 0.000 |
| originMiddle East | 1 | 1.109 | 1.083 | 1.135 | 0.000 |
| originEurope | 1 | 1.096 | 1.063 | 1.130 | 0.000 |
| originTurkey | 1 | 1.289 | 1.256 | 1.323 | 0.000 |
| originFormer Yugoslavia | 1 | 1.243 | 1.203 | 1.284 | 0.000 |
| originPakistan | 1 | 1.232 | 1.192 | 1.273 | 0.000 |
| originSri Lanka | 1 | 1.083 | 1.036 | 1.132 | 0.000 |
| originSomalia | 1 | 1.131 | 1.064 | 1.202 | 0.000 |
| originVietnam | 1 | 0.945 | 0.864 | 1.034 | 0.219 |
| sexM | 1 | 1.161 | 1.150 | 1.172 | 0.000 |
| Ns(age)1 | 1 | 0.692 | 0.681 | 0.704 | 0.000 |
| Ns(age)2 | 1 | 0.602 | 0.594 | 0.609 | 0.000 |
| Ns(age)3 | 1 | 0.679 | 0.670 | 0.688 | 0.000 |
| Ns(diabetes\_duration)1 | 1 | 1.776 | 1.747 | 1.805 | 0.000 |
| Ns(diabetes\_duration)2 | 1 | 2.654 | 2.598 | 2.710 | 0.000 |
| Ns(diabetes\_duration)3 | 1 | 2.013 | 1.985 | 2.041 | 0.000 |
| macrovasc\_compTRUE | 1 | 1.013 | 1.002 | 1.023 | 0.018 |
| dkdTRUE | 1 | 1.143 | 1.125 | 1.162 | 0.000 |

| Biomarker levels: Analysis of HbA1c levels | Model | Estimate | Lower 95% CI | Upper 95% CI | P-value |
| --- | --- | --- | --- | --- | --- |
| (Intercept) | 2 | 0.421 | 0.395 | 0.447 | 0.000 |
| originMiddle East | 2 | 1.057 | 1.030 | 1.085 | 0.000 |
| originEurope | 2 | 1.046 | 1.014 | 1.080 | 0.005 |
| originTurkey | 2 | 1.263 | 1.229 | 1.297 | 0.000 |
| originFormer Yugoslavia | 2 | 1.203 | 1.164 | 1.244 | 0.000 |
| originPakistan | 2 | 1.178 | 1.139 | 1.220 | 0.000 |
| originSri Lanka | 2 | 1.051 | 1.005 | 1.098 | 0.030 |
| originSomalia | 2 | 1.056 | 0.991 | 1.124 | 0.094 |
| originVietnam | 2 | 0.886 | 0.810 | 0.969 | 0.008 |
| sexM | 2 | 1.156 | 1.145 | 1.168 | 0.000 |
| Ns(age)1 | 2 | 0.740 | 0.721 | 0.760 | 0.000 |
| Ns(age)2 | 2 | 0.655 | 0.639 | 0.671 | 0.000 |
| Ns(age)3 | 2 | 0.712 | 0.696 | 0.728 | 0.000 |
| Ns(diabetes\_duration)1 | 2 | 1.779 | 1.750 | 1.808 | 0.000 |
| Ns(diabetes\_duration)2 | 2 | 2.688 | 2.632 | 2.746 | 0.000 |
| Ns(diabetes\_duration)3 | 2 | 2.015 | 1.987 | 2.043 | 0.000 |
| macrovasc\_compTRUE | 2 | 1.017 | 1.007 | 1.028 | 0.001 |
| dkdTRUE | 2 | 1.144 | 1.126 | 1.163 | 0.000 |
| employment\_statusRetired | 2 | 0.866 | 0.848 | 0.884 | 0.000 |
| employment\_statusUnemployed | 2 | 0.894 | 0.881 | 0.908 | 0.000 |
| Ns(household\_income)1 | 2 | 0.954 | 0.936 | 0.972 | 0.000 |
| Ns(household\_income)2 | 2 | 0.875 | 0.862 | 0.888 | 0.000 |
| Ns(household\_income)3 | 2 | 0.934 | 0.920 | 0.948 | 0.000 |
| duration\_of\_residence[10,15) | 2 | 0.777 | 0.712 | 0.847 | 0.000 |
| duration\_of\_residence[15,20) | 2 | 0.768 | 0.719 | 0.820 | 0.000 |
| duration\_of\_residence[20,Inf) | 2 | 0.749 | 0.707 | 0.793 | 0.000 |
| regionCentral Denmark Region | 2 | 0.974 | 0.961 | 0.988 | 0.000 |
| regionNorth Denmark Region | 2 | 1.117 | 1.099 | 1.135 | 0.000 |
| regionSouth Denmark Region | 2 | 1.120 | 1.105 | 1.135 | 0.000 |
| regionZealand Region | 2 | 0.982 | 0.967 | 0.997 | 0.016 |

### Biomarker levels: Analysis of LDL-C levels

| Biomarker levels: Analysis of LDL-C levels | Model | Estimate | Lower 95% CI | Upper 95% CI | P-value |
| --- | --- | --- | --- | --- | --- |
| Native Danes (reference risk) | 0 | 0.283 | 0.281 | 0.285 | 0.000 |
| originMiddle East | 0 | 1.172 | 1.134 | 1.210 | 0.000 |
| originEurope | 0 | 1.175 | 1.134 | 1.218 | 0.000 |
| originTurkey | 0 | 1.148 | 1.100 | 1.199 | 0.000 |
| originFormer Yugoslavia | 0 | 1.082 | 1.028 | 1.138 | 0.002 |
| originPakistan | 0 | 1.137 | 1.076 | 1.202 | 0.000 |
| originSri Lanka | 0 | 1.009 | 0.934 | 1.090 | 0.819 |
| originSomalia | 0 | 1.781 | 1.670 | 1.898 | 0.000 |
| originVietnam | 0 | 0.919 | 0.815 | 1.036 | 0.165 |

| Biomarker levels: Analysis of LDL-C levels | Model | Estimate | Lower 95% CI | Upper 95% CI | P-value |
| --- | --- | --- | --- | --- | --- |
| (Intercept) | 1 | 0.289 | 0.285 | 0.293 | 0.000 |
| originMiddle East | 1 | 1.092 | 1.057 | 1.127 | 0.000 |
| originEurope | 1 | 1.146 | 1.107 | 1.187 | 0.000 |
| originTurkey | 1 | 1.051 | 1.007 | 1.096 | 0.022 |
| originFormer Yugoslavia | 1 | 1.038 | 0.987 | 1.091 | 0.143 |
| originPakistan | 1 | 1.122 | 1.063 | 1.184 | 0.000 |
| originSri Lanka | 1 | 0.924 | 0.857 | 0.996 | 0.039 |
| originSomalia | 1 | 1.440 | 1.351 | 1.536 | 0.000 |
| originVietnam | 1 | 0.836 | 0.743 | 0.940 | 0.003 |
| sexM | 1 | 0.796 | 0.786 | 0.805 | 0.000 |
| Ns(age)1 | 1 | 0.712 | 0.696 | 0.727 | 0.000 |
| Ns(age)2 | 1 | 0.723 | 0.712 | 0.735 | 0.000 |
| Ns(age)3 | 1 | 0.860 | 0.845 | 0.875 | 0.000 |
| Ns(diabetes\_duration)1 | 1 | 0.782 | 0.763 | 0.800 | 0.000 |
| Ns(diabetes\_duration)2 | 1 | 0.587 | 0.576 | 0.598 | 0.000 |
| Ns(diabetes\_duration)3 | 1 | 0.768 | 0.754 | 0.782 | 0.000 |
| macrovasc\_compTRUE | 1 | 0.718 | 0.707 | 0.730 | 0.000 |
| dkdTRUE | 1 | 0.973 | 0.944 | 1.003 | 0.074 |

| Biomarker levels: Analysis of LDL-C levels | Model | Estimate | Lower 95% CI | Upper 95% CI | P-value |
| --- | --- | --- | --- | --- | --- |
| (Intercept) | 2 | 0.268 | 0.248 | 0.290 | 0.000 |
| originMiddle East | 2 | 1.092 | 1.053 | 1.133 | 0.000 |
| originEurope | 2 | 1.151 | 1.110 | 1.194 | 0.000 |
| originTurkey | 2 | 1.019 | 0.976 | 1.064 | 0.401 |
| originFormer Yugoslavia | 2 | 1.022 | 0.972 | 1.075 | 0.400 |
| originPakistan | 2 | 1.065 | 1.007 | 1.126 | 0.027 |
| originSri Lanka | 2 | 0.941 | 0.872 | 1.014 | 0.112 |
| originSomalia | 2 | 1.444 | 1.350 | 1.544 | 0.000 |
| originVietnam | 2 | 0.838 | 0.745 | 0.943 | 0.003 |
| sexM | 2 | 0.791 | 0.781 | 0.801 | 0.000 |
| Ns(age)1 | 2 | 0.764 | 0.737 | 0.792 | 0.000 |
| Ns(age)2 | 2 | 0.776 | 0.751 | 0.802 | 0.000 |
| Ns(age)3 | 2 | 0.916 | 0.888 | 0.945 | 0.000 |
| Ns(diabetes\_duration)1 | 2 | 0.779 | 0.761 | 0.798 | 0.000 |
| Ns(diabetes\_duration)2 | 2 | 0.587 | 0.576 | 0.598 | 0.000 |
| Ns(diabetes\_duration)3 | 2 | 0.766 | 0.752 | 0.780 | 0.000 |
| macrovasc\_compTRUE | 2 | 0.720 | 0.708 | 0.731 | 0.000 |
| dkdTRUE | 2 | 0.976 | 0.947 | 1.006 | 0.117 |
| employment\_statusRetired | 2 | 0.921 | 0.895 | 0.947 | 0.000 |
| employment\_statusUnemployed | 2 | 0.962 | 0.944 | 0.981 | 0.000 |
| Ns(household\_income)1 | 2 | 0.983 | 0.959 | 1.007 | 0.162 |
| Ns(household\_income)2 | 2 | 0.950 | 0.932 | 0.969 | 0.000 |
| Ns(household\_income)3 | 2 | 1.012 | 0.993 | 1.031 | 0.228 |
| duration\_of\_residence[10,15) | 2 | 1.142 | 1.029 | 1.268 | 0.013 |
| duration\_of\_residence[15,20) | 2 | 1.100 | 1.012 | 1.195 | 0.025 |
| duration\_of\_residence[20,Inf) | 2 | 1.177 | 1.097 | 1.264 | 0.000 |
| regionCentral Denmark Region | 2 | 0.911 | 0.895 | 0.927 | 0.000 |
| regionNorth Denmark Region | 2 | 0.858 | 0.839 | 0.878 | 0.000 |
| regionSouth Denmark Region | 2 | 0.985 | 0.968 | 1.002 | 0.081 |
| regionZealand Region | 2 | 0.958 | 0.940 | 0.976 | 0.000 |

### Pharmacological treatment: Analysis of glucose-lowering drugs

| Pharmacological treatment: Analysis of glucose-lowering drugs | Model | Estimate | Lower 95% CI | Upper 95% CI | P-value |
| --- | --- | --- | --- | --- | --- |
| Native Danes (reference risk) | 0 | 0.069 | 0.068 | 0.070 | 0.000 |
| originMiddle East | 0 | 0.922 | 0.827 | 1.028 | 0.146 |
| originEurope | 0 | 1.447 | 1.307 | 1.602 | 0.000 |
| originTurkey | 0 | 0.739 | 0.634 | 0.862 | 0.000 |
| originFormer Yugoslavia | 0 | 0.836 | 0.708 | 0.987 | 0.034 |
| originPakistan | 0 | 1.142 | 0.977 | 1.335 | 0.095 |
| originSri Lanka | 0 | 0.607 | 0.461 | 0.800 | 0.000 |
| originSomalia | 0 | 1.674 | 1.342 | 2.089 | 0.000 |
| originVietnam | 0 | 1.046 | 0.753 | 1.455 | 0.787 |

| Pharmacological treatment: Analysis of glucose-lowering drugs | Model | Estimate | Lower 95% CI | Upper 95% CI | P-value |
| --- | --- | --- | --- | --- | --- |
| (Intercept) | 1 | 0.032 | 0.030 | 0.033 | 0.000 |
| originMiddle East | 1 | 1.144 | 1.028 | 1.272 | 0.013 |
| originEurope | 1 | 1.282 | 1.165 | 1.412 | 0.000 |
| originTurkey | 1 | 0.933 | 0.803 | 1.084 | 0.364 |
| originFormer Yugoslavia | 1 | 0.946 | 0.808 | 1.109 | 0.495 |
| originPakistan | 1 | 1.660 | 1.431 | 1.925 | 0.000 |
| originSri Lanka | 1 | 0.950 | 0.730 | 1.234 | 0.699 |
| originSomalia | 1 | 1.876 | 1.508 | 2.335 | 0.000 |
| originVietnam | 1 | 1.072 | 0.778 | 1.477 | 0.672 |
| sexM | 1 | 0.853 | 0.822 | 0.885 | 0.000 |
| Ns(age)1 | 1 | 1.296 | 1.214 | 1.382 | 0.000 |
| Ns(age)2 | 1 | 1.573 | 1.496 | 1.654 | 0.000 |
| Ns(age)3 | 1 | 2.029 | 1.922 | 2.143 | 0.000 |
| Ns(diabetes\_duration)1 | 1 | 0.315 | 0.287 | 0.346 | 0.000 |
| Ns(diabetes\_duration)2 | 1 | 0.085 | 0.079 | 0.090 | 0.000 |
| Ns(diabetes\_duration)3 | 1 | 0.253 | 0.236 | 0.271 | 0.000 |
| macrovasc\_compTRUE | 1 | 1.093 | 1.050 | 1.139 | 0.000 |
| dkdTRUE | 1 | 0.818 | 0.738 | 0.908 | 0.000 |

| Pharmacological treatment: Analysis of glucose-lowering drugs | Model | Estimate | Lower 95% CI | Upper 95% CI | P-value |
| --- | --- | --- | --- | --- | --- |
| (Intercept) | 2 | 0.033 | 0.026 | 0.040 | 0.000 |
| originMiddle East | 2 | 1.021 | 0.904 | 1.152 | 0.739 |
| originEurope | 2 | 1.230 | 1.113 | 1.359 | 0.000 |
| originTurkey | 2 | 0.765 | 0.656 | 0.892 | 0.001 |
| originFormer Yugoslavia | 2 | 0.846 | 0.720 | 0.994 | 0.042 |
| originPakistan | 2 | 1.244 | 1.067 | 1.451 | 0.005 |
| originSri Lanka | 2 | 0.971 | 0.745 | 1.264 | 0.825 |
| originSomalia | 2 | 1.660 | 1.315 | 2.097 | 0.000 |
| originVietnam | 2 | 1.051 | 0.760 | 1.454 | 0.764 |
| sexM | 2 | 0.863 | 0.831 | 0.896 | 0.000 |
| Ns(age)1 | 2 | 1.182 | 1.063 | 1.315 | 0.002 |
| Ns(age)2 | 2 | 1.432 | 1.293 | 1.587 | 0.000 |
| Ns(age)3 | 2 | 1.852 | 1.683 | 2.038 | 0.000 |
| Ns(diabetes\_duration)1 | 2 | 0.315 | 0.287 | 0.345 | 0.000 |
| Ns(diabetes\_duration)2 | 2 | 0.084 | 0.079 | 0.090 | 0.000 |
| Ns(diabetes\_duration)3 | 2 | 0.252 | 0.235 | 0.270 | 0.000 |
| macrovasc\_compTRUE | 2 | 1.084 | 1.041 | 1.129 | 0.000 |
| dkdTRUE | 2 | 0.816 | 0.735 | 0.906 | 0.000 |
| employment\_statusRetired | 2 | 1.069 | 0.976 | 1.171 | 0.149 |
| employment\_statusUnemployed | 2 | 0.978 | 0.914 | 1.046 | 0.520 |
| Ns(household\_income)1 | 2 | 0.970 | 0.901 | 1.044 | 0.418 |
| Ns(household\_income)2 | 2 | 0.852 | 0.803 | 0.904 | 0.000 |
| Ns(household\_income)3 | 2 | 0.982 | 0.928 | 1.040 | 0.533 |
| duration\_of\_residence[10,15) | 2 | 1.074 | 0.777 | 1.485 | 0.665 |
| duration\_of\_residence[15,20) | 2 | 1.007 | 0.790 | 1.285 | 0.954 |
| duration\_of\_residence[20,Inf) | 2 | 1.163 | 0.957 | 1.413 | 0.129 |
| regionCentral Denmark Region | 2 | 0.785 | 0.745 | 0.826 | 0.000 |
| regionNorth Denmark Region | 2 | 0.589 | 0.548 | 0.634 | 0.000 |
| regionSouth Denmark Region | 2 | 0.693 | 0.659 | 0.728 | 0.000 |
| regionZealand Region | 2 | 0.719 | 0.678 | 0.763 | 0.000 |

### Pharmacological treatment: Analysis of lipid-lowering drugs

| Pharmacological treatment: Analysis of lipid-lowering drugs | Model | Estimate | Lower 95% CI | Upper 95% CI | P-value |
| --- | --- | --- | --- | --- | --- |
| Native Danes (reference risk) | 0 | 0.349 | 0.346 | 0.351 | 0.000 |
| originMiddle East | 0 | 1.005 | 0.963 | 1.048 | 0.826 |
| originEurope | 0 | 1.136 | 1.089 | 1.186 | 0.000 |
| originTurkey | 0 | 0.912 | 0.860 | 0.967 | 0.002 |
| originFormer Yugoslavia | 0 | 0.817 | 0.760 | 0.878 | 0.000 |
| originPakistan | 0 | 0.909 | 0.843 | 0.980 | 0.013 |
| originSri Lanka | 0 | 0.893 | 0.806 | 0.990 | 0.032 |
| originSomalia | 0 | 1.643 | 1.525 | 1.770 | 0.000 |
| originVietnam | 0 | 0.851 | 0.725 | 0.999 | 0.049 |

| Pharmacological treatment: Analysis of lipid-lowering drugs | Model | Estimate | Lower 95% CI | Upper 95% CI | P-value |
| --- | --- | --- | --- | --- | --- |
| (Intercept) | 1 | 0.492 | 0.484 | 0.500 | 0.000 |
| originMiddle East | 1 | 0.983 | 0.946 | 1.022 | 0.382 |
| originEurope | 1 | 1.087 | 1.046 | 1.129 | 0.000 |
| originTurkey | 1 | 0.886 | 0.838 | 0.936 | 0.000 |
| originFormer Yugoslavia | 1 | 0.855 | 0.801 | 0.914 | 0.000 |
| originPakistan | 1 | 0.953 | 0.889 | 1.021 | 0.170 |
| originSri Lanka | 1 | 0.854 | 0.777 | 0.938 | 0.001 |
| originSomalia | 1 | 1.160 | 1.076 | 1.250 | 0.000 |
| originVietnam | 1 | 0.791 | 0.681 | 0.919 | 0.002 |
| sexM | 1 | 0.918 | 0.905 | 0.931 | 0.000 |
| Ns(age)1 | 1 | 0.855 | 0.834 | 0.876 | 0.000 |
| Ns(age)2 | 1 | 0.894 | 0.878 | 0.911 | 0.000 |
| Ns(age)3 | 1 | 1.183 | 1.158 | 1.207 | 0.000 |
| Ns(diabetes\_duration)1 | 1 | 0.925 | 0.901 | 0.949 | 0.000 |
| Ns(diabetes\_duration)2 | 1 | 0.886 | 0.867 | 0.904 | 0.000 |
| Ns(diabetes\_duration)3 | 1 | 0.917 | 0.899 | 0.935 | 0.000 |
| macrovasc\_compTRUE | 1 | 0.322 | 0.316 | 0.328 | 0.000 |
| dkdTRUE | 1 | 0.555 | 0.536 | 0.573 | 0.000 |

| Pharmacological treatment: Analysis of lipid-lowering drugs | Model | Estimate | Lower 95% CI | Upper 95% CI | P-value |
| --- | --- | --- | --- | --- | --- |
| (Intercept) | 2 | 0.451 | 0.408 | 0.500 | 0.000 |
| originMiddle East | 2 | 0.967 | 0.926 | 1.010 | 0.128 |
| originEurope | 2 | 1.094 | 1.051 | 1.139 | 0.000 |
| originTurkey | 2 | 0.841 | 0.795 | 0.890 | 0.000 |
| originFormer Yugoslavia | 2 | 0.843 | 0.788 | 0.901 | 0.000 |
| originPakistan | 2 | 0.892 | 0.831 | 0.957 | 0.002 |
| originSri Lanka | 2 | 0.890 | 0.810 | 0.978 | 0.016 |
| originSomalia | 2 | 1.169 | 1.081 | 1.264 | 0.000 |
| originVietnam | 2 | 0.816 | 0.702 | 0.947 | 0.008 |
| sexM | 2 | 0.922 | 0.909 | 0.935 | 0.000 |
| Ns(age)1 | 2 | 0.848 | 0.814 | 0.883 | 0.000 |
| Ns(age)2 | 2 | 0.884 | 0.851 | 0.919 | 0.000 |
| Ns(age)3 | 2 | 1.176 | 1.134 | 1.219 | 0.000 |
| Ns(diabetes\_duration)1 | 2 | 0.925 | 0.901 | 0.949 | 0.000 |
| Ns(diabetes\_duration)2 | 2 | 0.879 | 0.861 | 0.898 | 0.000 |
| Ns(diabetes\_duration)3 | 2 | 0.915 | 0.897 | 0.934 | 0.000 |
| macrovasc\_compTRUE | 2 | 0.321 | 0.316 | 0.327 | 0.000 |
| dkdTRUE | 2 | 0.551 | 0.533 | 0.570 | 0.000 |
| employment\_statusRetired | 2 | 1.014 | 0.980 | 1.049 | 0.426 |
| employment\_statusUnemployed | 2 | 1.023 | 1.000 | 1.047 | 0.050 |
| Ns(household\_income)1 | 2 | 0.976 | 0.950 | 1.003 | 0.077 |
| Ns(household\_income)2 | 2 | 0.966 | 0.945 | 0.988 | 0.002 |
| Ns(household\_income)3 | 2 | 0.988 | 0.968 | 1.009 | 0.266 |
| duration\_of\_residence[10,15) | 2 | 1.096 | 0.953 | 1.260 | 0.201 |
| duration\_of\_residence[15,20) | 2 | 1.070 | 0.959 | 1.194 | 0.228 |
| duration\_of\_residence[20,Inf) | 2 | 1.138 | 1.035 | 1.252 | 0.008 |
| regionCentral Denmark Region | 2 | 0.863 | 0.846 | 0.880 | 0.000 |
| regionNorth Denmark Region | 2 | 0.902 | 0.879 | 0.925 | 0.000 |
| regionSouth Denmark Region | 2 | 0.860 | 0.843 | 0.877 | 0.000 |
| regionZealand Region | 2 | 1.054 | 1.033 | 1.074 | 0.000 |

### Pharmacological treatment: Analysis of ACEI/ARB

| Pharmacological treatment: Analysis of ACEI/ARB | Model | Estimate | Lower 95% CI | Upper 95% CI | P-value |
| --- | --- | --- | --- | --- | --- |
| Native Danes (reference risk) | 0 | 0.284 | 0.281 | 0.288 | 0.000 |
| originMiddle East | 0 | 1.251 | 1.184 | 1.322 | 0.000 |
| originEurope | 0 | 1.020 | 0.950 | 1.096 | 0.577 |
| originTurkey | 0 | 1.242 | 1.154 | 1.336 | 0.000 |
| originFormer Yugoslavia | 0 | 1.069 | 0.979 | 1.167 | 0.138 |
| originPakistan | 0 | 1.297 | 1.189 | 1.415 | 0.000 |
| originSri Lanka | 0 | 1.282 | 1.132 | 1.451 | 0.000 |
| originSomalia | 0 | 1.504 | 1.245 | 1.816 | 0.000 |
| originVietnam | 0 | 0.870 | 0.683 | 1.110 | 0.263 |

| Pharmacological treatment: Analysis of ACEI/ARB | Model | Estimate | Lower 95% CI | Upper 95% CI | P-value |
| --- | --- | --- | --- | --- | --- |
| (Intercept) | 1 | 0.240 | 0.222 | 0.259 | 0.000 |
| originMiddle East | 1 | 1.294 | 1.225 | 1.368 | 0.000 |
| originEurope | 1 | 1.008 | 0.939 | 1.082 | 0.823 |
| originTurkey | 1 | 1.250 | 1.164 | 1.343 | 0.000 |
| originFormer Yugoslavia | 1 | 1.079 | 0.990 | 1.176 | 0.084 |
| originPakistan | 1 | 1.415 | 1.298 | 1.542 | 0.000 |
| originSri Lanka | 1 | 1.377 | 1.225 | 1.549 | 0.000 |
| originSomalia | 1 | 1.492 | 1.242 | 1.793 | 0.000 |
| originVietnam | 1 | 0.914 | 0.720 | 1.160 | 0.461 |
| sexM | 1 | 0.833 | 0.814 | 0.851 | 0.000 |
| Ns(age)1 | 1 | 0.833 | 0.802 | 0.865 | 0.000 |
| Ns(age)2 | 1 | 0.768 | 0.743 | 0.794 | 0.000 |
| Ns(age)3 | 1 | 1.083 | 1.049 | 1.119 | 0.000 |
| Ns(diabetes\_duration)1 | 1 | 0.882 | 0.848 | 0.918 | 0.000 |
| Ns(diabetes\_duration)2 | 1 | 0.722 | 0.697 | 0.747 | 0.000 |
| Ns(diabetes\_duration)3 | 1 | 0.845 | 0.819 | 0.872 | 0.000 |
| macrovasc\_compTRUE | 1 | 1.167 | 1.084 | 1.255 | 0.000 |
| dkdTRUE | 1 | 0.685 | 0.648 | 0.725 | 0.000 |

| Pharmacological treatment: Analysis of ACEI/ARB | Model | Estimate | Lower 95% CI | Upper 95% CI | P-value |
| --- | --- | --- | --- | --- | --- |
| (Intercept) | 2 | 0.225 | 0.183 | 0.278 | 0.000 |
| originMiddle East | 2 | 1.198 | 1.125 | 1.276 | 0.000 |
| originEurope | 2 | 0.995 | 0.925 | 1.069 | 0.881 |
| originTurkey | 2 | 1.159 | 1.076 | 1.248 | 0.000 |
| originFormer Yugoslavia | 2 | 1.004 | 0.919 | 1.096 | 0.935 |
| originPakistan | 2 | 1.311 | 1.198 | 1.434 | 0.000 |
| originSri Lanka | 2 | 1.337 | 1.187 | 1.506 | 0.000 |
| originSomalia | 2 | 1.363 | 1.131 | 1.642 | 0.001 |
| originVietnam | 2 | 0.890 | 0.701 | 1.129 | 0.336 |
| sexM | 2 | 0.849 | 0.830 | 0.868 | 0.000 |
| Ns(age)1 | 2 | 0.862 | 0.810 | 0.917 | 0.000 |
| Ns(age)2 | 2 | 0.800 | 0.752 | 0.852 | 0.000 |
| Ns(age)3 | 2 | 1.112 | 1.052 | 1.175 | 0.000 |
| Ns(diabetes\_duration)1 | 2 | 0.874 | 0.840 | 0.909 | 0.000 |
| Ns(diabetes\_duration)2 | 2 | 0.714 | 0.689 | 0.739 | 0.000 |
| Ns(diabetes\_duration)3 | 2 | 0.837 | 0.811 | 0.863 | 0.000 |
| macrovasc\_compTRUE | 2 | 1.152 | 1.071 | 1.239 | 0.000 |
| dkdTRUE | 2 | 0.681 | 0.644 | 0.720 | 0.000 |
| employment\_statusRetired | 2 | 1.050 | 0.994 | 1.109 | 0.080 |
| employment\_statusUnemployed | 2 | 1.197 | 1.147 | 1.249 | 0.000 |
| Ns(household\_income)1 | 2 | 0.963 | 0.921 | 1.006 | 0.094 |
| Ns(household\_income)2 | 2 | 0.958 | 0.924 | 0.992 | 0.017 |
| Ns(household\_income)3 | 2 | 0.933 | 0.902 | 0.964 | 0.000 |
| duration\_of\_residence[10,15) | 2 | 1.248 | 0.985 | 1.582 | 0.067 |
| duration\_of\_residence[15,20) | 2 | 1.082 | 0.884 | 1.326 | 0.443 |
| duration\_of\_residence[20,Inf) | 2 | 1.109 | 0.924 | 1.332 | 0.266 |
| regionCentral Denmark Region | 2 | 0.913 | 0.885 | 0.941 | 0.000 |
| regionNorth Denmark Region | 2 | 0.832 | 0.797 | 0.868 | 0.000 |
| regionSouth Denmark Region | 2 | 0.934 | 0.906 | 0.964 | 0.000 |
| regionZealand Region | 2 | 0.948 | 0.917 | 0.980 | 0.002 |

### Pharmacological treatment: Analysis of antiplatelet therapy

| Pharmacological treatment: Analysis of antiplatelet therapy | Model | Estimate | Lower 95% CI | Upper 95% CI | P-value |
| --- | --- | --- | --- | --- | --- |
| Native Danes (reference risk) | 0 | 0.348 | 0.345 | 0.352 | 0.000 |
| originMiddle East | 0 | 1.034 | 0.979 | 1.092 | 0.233 |
| originEurope | 0 | 1.031 | 0.970 | 1.095 | 0.329 |
| originTurkey | 0 | 0.955 | 0.885 | 1.030 | 0.233 |
| originFormer Yugoslavia | 0 | 1.077 | 0.999 | 1.161 | 0.054 |
| originPakistan | 0 | 0.822 | 0.740 | 0.913 | 0.000 |
| originSri Lanka | 0 | 0.949 | 0.830 | 1.084 | 0.440 |
| originSomalia | 0 | 1.525 | 1.309 | 1.778 | 0.000 |
| originVietnam | 0 | 1.117 | 0.938 | 1.331 | 0.215 |

| Pharmacological treatment: Analysis of antiplatelet therapy | Model | Estimate | Lower 95% CI | Upper 95% CI | P-value |
| --- | --- | --- | --- | --- | --- |
| (Intercept) | 1 | 0.631 | 0.601 | 0.663 | 0.000 |
| originMiddle East | 1 | 1.046 | 0.993 | 1.103 | 0.093 |
| originEurope | 1 | 1.050 | 0.989 | 1.114 | 0.108 |
| originTurkey | 1 | 0.953 | 0.884 | 1.027 | 0.206 |
| originFormer Yugoslavia | 1 | 1.080 | 1.004 | 1.161 | 0.039 |
| originPakistan | 1 | 0.892 | 0.807 | 0.986 | 0.025 |
| originSri Lanka | 1 | 0.927 | 0.818 | 1.050 | 0.234 |
| originSomalia | 1 | 1.342 | 1.154 | 1.560 | 0.000 |
| originVietnam | 1 | 0.955 | 0.815 | 1.119 | 0.569 |
| sexM | 1 | 0.863 | 0.847 | 0.880 | 0.000 |
| Ns(age)1 | 1 | 0.997 | 0.965 | 1.030 | 0.843 |
| Ns(age)2 | 1 | 0.875 | 0.852 | 0.898 | 0.000 |
| Ns(age)3 | 1 | 1.150 | 1.119 | 1.182 | 0.000 |
| Ns(diabetes\_duration)1 | 1 | 0.951 | 0.920 | 0.983 | 0.003 |
| Ns(diabetes\_duration)2 | 1 | 0.859 | 0.832 | 0.886 | 0.000 |
| Ns(diabetes\_duration)3 | 1 | 0.900 | 0.876 | 0.924 | 0.000 |
| macrovasc\_compTRUE | 1 | 0.514 | 0.493 | 0.537 | 0.000 |
| dkdTRUE | 1 | 1.080 | 1.036 | 1.125 | 0.000 |

| Pharmacological treatment: Analysis of antiplatelet therapy | Model | Estimate | Lower 95% CI | Upper 95% CI | P-value |
| --- | --- | --- | --- | --- | --- |
| (Intercept) | 2 | 0.540 | 0.449 | 0.649 | 0.000 |
| originMiddle East | 2 | 1.023 | 0.964 | 1.085 | 0.455 |
| originEurope | 2 | 1.044 | 0.982 | 1.109 | 0.166 |
| originTurkey | 2 | 0.931 | 0.863 | 1.005 | 0.067 |
| originFormer Yugoslavia | 2 | 1.064 | 0.988 | 1.146 | 0.100 |
| originPakistan | 2 | 0.850 | 0.767 | 0.942 | 0.002 |
| originSri Lanka | 2 | 0.958 | 0.845 | 1.085 | 0.499 |
| originSomalia | 2 | 1.320 | 1.131 | 1.542 | 0.000 |
| originVietnam | 2 | 0.978 | 0.834 | 1.147 | 0.781 |
| sexM | 2 | 0.862 | 0.846 | 0.879 | 0.000 |
| Ns(age)1 | 2 | 1.022 | 0.970 | 1.076 | 0.424 |
| Ns(age)2 | 2 | 0.891 | 0.847 | 0.937 | 0.000 |
| Ns(age)3 | 2 | 1.181 | 1.128 | 1.237 | 0.000 |
| Ns(diabetes\_duration)1 | 2 | 0.949 | 0.918 | 0.981 | 0.002 |
| Ns(diabetes\_duration)2 | 2 | 0.855 | 0.828 | 0.882 | 0.000 |
| Ns(diabetes\_duration)3 | 2 | 0.898 | 0.874 | 0.922 | 0.000 |
| macrovasc\_compTRUE | 2 | 0.515 | 0.493 | 0.537 | 0.000 |
| dkdTRUE | 2 | 1.081 | 1.037 | 1.126 | 0.000 |
| employment\_statusRetired | 2 | 1.038 | 0.992 | 1.085 | 0.108 |
| employment\_statusUnemployed | 2 | 1.082 | 1.045 | 1.122 | 0.000 |
| Ns(household\_income)1 | 2 | 1.023 | 0.985 | 1.062 | 0.236 |
| Ns(household\_income)2 | 2 | 1.040 | 1.009 | 1.073 | 0.012 |
| Ns(household\_income)3 | 2 | 1.063 | 1.033 | 1.093 | 0.000 |
| duration\_of\_residence[10,15) | 2 | 1.180 | 0.938 | 1.483 | 0.157 |
| duration\_of\_residence[15,20) | 2 | 1.267 | 1.052 | 1.526 | 0.013 |
| duration\_of\_residence[20,Inf) | 2 | 1.186 | 1.001 | 1.406 | 0.049 |
| regionCentral Denmark Region | 2 | 0.878 | 0.855 | 0.902 | 0.000 |
| regionNorth Denmark Region | 2 | 0.919 | 0.888 | 0.950 | 0.000 |
| regionSouth Denmark Region | 2 | 0.897 | 0.873 | 0.922 | 0.000 |
| regionZealand Region | 2 | 0.956 | 0.929 | 0.983 | 0.001 |
