# Supplementary material for: Guideline-level monitoring, biomarker levels and pharmacological treatment in migrants and native Danes with type 2 diabetes: Population-wide analyses
Source: PLOS Glob Public Health. 2023 Oct 18;3(10):e0001277. doi: 10.1371/journal.pgph.0001277 (PMC10584163; doi:10.1371/journal.pgph.0001277)
Supplement: S7 File — (HTML) [file pgph.0001277.s007.html]

S7. Regression coefficients of absolute risk difference.


# S7. Regression coefficients of absolute risk difference.

- S7. Regression coefficients of absolute risk difference.
  - Prevalence: Analysis of prevalent type 2 diabetes
  - Monitoring: Analysis of HbA1c monitoring
  - Monitoring: Analysis of LDL-C monitoring
  - Monitoring: Analysis of screening for diabetic nephropathy
  - Monitoring: Analysis of screening for diabetic retinopathy
  - Monitoring: Analysis of screening for diabetic foot disease
  - Biomarker levels: Analysis of HbA1c levels
  - Biomarker levels: Analysis of LDL-C levels
  - Pharmacological treatment: Analysis of glucose-lowering drugs
  - Pharmacological treatment: Analysis of lipid-lowering drugs
  - Pharmacological treatment: Analysis of ACEI/ARB
  - Pharmacological treatment: Analysis of antiplatelet therapy

# S7. Regression coefficients of absolute risk difference.

Note: Risk differences and 95% confidence intervals computed using a gaussian distribution and identity link with robust (*sandwich*) variance estimates.

### Prevalence: Analysis of prevalent type 2 diabetes

| Prevalence: Analysis of prevalent type 2 diabetes | Model | Estimate | Lower 95% CI | Upper 95% CI | P-value |
| --- | --- | --- | --- | --- | --- |
| (Intercept) | 0 | 0.066 | 0.066 | 0.066 | 0 |
| originMiddle East | 0 | 0.044 | 0.042 | 0.046 | 0 |
| originEurope | 0 | -0.025 | -0.026 | -0.024 | 0 |
| originTurkey | 0 | 0.087 | 0.083 | 0.091 | 0 |
| originFormer Yugoslavia | 0 | 0.045 | 0.042 | 0.048 | 0 |
| originPakistan | 0 | 0.174 | 0.166 | 0.182 | 0 |
| originSri Lanka | 0 | 0.197 | 0.186 | 0.208 | 0 |
| originSomalia | 0 | 0.043 | 0.037 | 0.050 | 0 |
| originVietnam | 0 | 0.028 | 0.022 | 0.034 | 0 |

| Prevalence: Analysis of prevalent type 2 diabetes | Model | Estimate | Lower 95% CI | Upper 95% CI | P-value |
| --- | --- | --- | --- | --- | --- |
| (Intercept) | 1 | 0.042 | 0.041 | 0.042 | 0.000 |
| originMiddle East | 1 | 0.071 | 0.069 | 0.074 | 0.000 |
| originEurope | 1 | -0.001 | -0.002 | 0.000 | 0.006 |
| originTurkey | 1 | 0.111 | 0.107 | 0.115 | 0.000 |
| originFormer Yugoslavia | 1 | 0.065 | 0.062 | 0.069 | 0.000 |
| originPakistan | 1 | 0.194 | 0.187 | 0.202 | 0.000 |
| originSri Lanka | 1 | 0.217 | 0.207 | 0.227 | 0.000 |
| originSomalia | 1 | 0.082 | 0.076 | 0.088 | 0.000 |
| originVietnam | 1 | 0.051 | 0.045 | 0.057 | 0.000 |
| sexM | 1 | 0.023 | 0.022 | 0.023 | 0.000 |
| Ns(Age)1 | 1 | 0.084 | 0.083 | 0.085 | 0.000 |
| Ns(Age)2 | 1 | 0.116 | 0.115 | 0.117 | 0.000 |
| Ns(Age)3 | 1 | 0.116 | 0.115 | 0.116 | 0.000 |

| Prevalence: Analysis of prevalent type 2 diabetes | Model | Estimate | Lower 95% CI | Upper 95% CI | P-value |
| --- | --- | --- | --- | --- | --- |
| (Intercept) | 2 | 0.006 | 0.003 | 0.008 | 0 |
| originMiddle East | 2 | 0.062 | 0.059 | 0.064 | 0 |
| originEurope | 2 | 0.018 | 0.017 | 0.020 | 0 |
| originTurkey | 2 | 0.093 | 0.089 | 0.097 | 0 |
| originFormer Yugoslavia | 2 | 0.052 | 0.048 | 0.055 | 0 |
| originPakistan | 2 | 0.188 | 0.181 | 0.195 | 0 |
| originSri Lanka | 2 | 0.206 | 0.196 | 0.216 | 0 |
| originSomalia | 2 | 0.064 | 0.058 | 0.070 | 0 |
| originVietnam | 2 | 0.044 | 0.038 | 0.050 | 0 |
| sexM | 2 | 0.025 | 0.025 | 0.026 | 0 |
| Ns(Age)1 | 2 | 0.082 | 0.081 | 0.083 | 0 |
| Ns(Age)2 | 2 | 0.114 | 0.113 | 0.115 | 0 |
| Ns(Age)3 | 2 | 0.097 | 0.096 | 0.098 | 0 |
| employment\_statusRetired | 2 | 0.023 | 0.021 | 0.024 | 0 |
| employment\_statusUnemployed | 2 | 0.039 | 0.038 | 0.040 | 0 |
| Ns(household\_income)1 | 2 | -0.025 | -0.026 | -0.024 | 0 |
| Ns(household\_income)2 | 2 | -0.034 | -0.035 | -0.033 | 0 |
| Ns(household\_income)3 | 2 | -0.036 | -0.037 | -0.036 | 0 |
| duration\_of\_residence[10,15) | 2 | 0.008 | 0.006 | 0.010 | 0 |
| duration\_of\_residence[15,20) | 2 | 0.008 | 0.006 | 0.011 | 0 |
| duration\_of\_residence[20,Inf) | 2 | 0.041 | 0.039 | 0.043 | 0 |
| regionCentral Denmark Region | 2 | -0.002 | -0.003 | -0.002 | 0 |
| regionNorth Denmark Region | 2 | -0.003 | -0.003 | -0.002 | 0 |
| regionSouth Denmark Region | 2 | -0.004 | -0.005 | -0.003 | 0 |
| regionZealand Region | 2 | 0.003 | 0.002 | 0.004 | 0 |

### Monitoring: Analysis of HbA1c monitoring

| Monitoring: Analysis of HbA1c monitoring | Model | Estimate | Lower 95% CI | Upper 95% CI | P-value |
| --- | --- | --- | --- | --- | --- |
| (Intercept) | 0 | 0.068 | 0.067 | 0.069 | 0.000 |
| originMiddle East | 0 | 0.012 | 0.006 | 0.018 | 0.000 |
| originEurope | 0 | 0.022 | 0.015 | 0.029 | 0.000 |
| originTurkey | 0 | -0.001 | -0.009 | 0.006 | 0.723 |
| originFormer Yugoslavia | 0 | -0.003 | -0.011 | 0.005 | 0.484 |
| originPakistan | 0 | 0.008 | -0.002 | 0.018 | 0.116 |
| originSri Lanka | 0 | -0.025 | -0.035 | -0.015 | 0.000 |
| originSomalia | 0 | 0.043 | 0.023 | 0.062 | 0.000 |
| originVietnam | 0 | 0.027 | 0.006 | 0.047 | 0.011 |

| Monitoring: Analysis of HbA1c monitoring | Model | Estimate | Lower 95% CI | Upper 95% CI | P-value |
| --- | --- | --- | --- | --- | --- |
| (Intercept) | 1 | 0.073 | 0.070 | 0.075 | 0.000 |
| originMiddle East | 1 | -0.003 | -0.010 | 0.003 | 0.273 |
| originEurope | 1 | 0.022 | 0.015 | 0.029 | 0.000 |
| originTurkey | 1 | -0.017 | -0.025 | -0.010 | 0.000 |
| originFormer Yugoslavia | 1 | -0.010 | -0.019 | -0.002 | 0.014 |
| originPakistan | 1 | -0.003 | -0.013 | 0.007 | 0.554 |
| originSri Lanka | 1 | -0.045 | -0.055 | -0.035 | 0.000 |
| originSomalia | 1 | 0.008 | -0.012 | 0.029 | 0.415 |
| originVietnam | 1 | 0.019 | -0.002 | 0.039 | 0.071 |
| sexM | 1 | -0.003 | -0.005 | -0.001 | 0.002 |
| Ns(age)1 | 1 | -0.049 | -0.053 | -0.046 | 0.000 |
| Ns(age)2 | 1 | -0.067 | -0.070 | -0.064 | 0.000 |
| Ns(age)3 | 1 | -0.027 | -0.030 | -0.024 | 0.000 |
| Ns(diabetes\_duration)1 | 1 | -0.001 | -0.004 | 0.003 | 0.766 |
| Ns(diabetes\_duration)2 | 1 | 0.035 | 0.032 | 0.038 | 0.000 |
| Ns(diabetes\_duration)3 | 1 | -0.009 | -0.012 | -0.006 | 0.000 |
| macrovasc\_compTRUE | 1 | -0.011 | -0.013 | -0.009 | 0.000 |
| dkdTRUE | 1 | -0.049 | -0.051 | -0.046 | 0.000 |

| Monitoring: Analysis of HbA1c monitoring | Model | Estimate | Lower 95% CI | Upper 95% CI | P-value |
| --- | --- | --- | --- | --- | --- |
| (Intercept) | 2 | 0.106 | 0.088 | 0.124 | 0.000 |
| originMiddle East | 2 | -0.008 | -0.015 | -0.001 | 0.026 |
| originEurope | 2 | 0.019 | 0.012 | 0.026 | 0.000 |
| originTurkey | 2 | -0.030 | -0.038 | -0.022 | 0.000 |
| originFormer Yugoslavia | 2 | -0.013 | -0.021 | -0.005 | 0.003 |
| originPakistan | 2 | -0.028 | -0.039 | -0.018 | 0.000 |
| originSri Lanka | 2 | -0.037 | -0.048 | -0.027 | 0.000 |
| originSomalia | 2 | 0.006 | -0.014 | 0.027 | 0.549 |
| originVietnam | 2 | 0.021 | 0.001 | 0.042 | 0.043 |
| sexM | 2 | -0.005 | -0.007 | -0.003 | 0.000 |
| Ns(age)1 | 2 | -0.049 | -0.054 | -0.044 | 0.000 |
| Ns(age)2 | 2 | -0.066 | -0.071 | -0.061 | 0.000 |
| Ns(age)3 | 2 | -0.028 | -0.033 | -0.024 | 0.000 |
| Ns(diabetes\_duration)1 | 2 | 0.000 | -0.004 | 0.004 | 0.982 |
| Ns(diabetes\_duration)2 | 2 | 0.034 | 0.031 | 0.037 | 0.000 |
| Ns(diabetes\_duration)3 | 2 | -0.008 | -0.011 | -0.005 | 0.000 |
| macrovasc\_compTRUE | 2 | -0.010 | -0.012 | -0.008 | 0.000 |
| dkdTRUE | 2 | -0.048 | -0.051 | -0.046 | 0.000 |
| employment\_statusRetired | 2 | -0.018 | -0.023 | -0.014 | 0.000 |
| employment\_statusUnemployed | 2 | -0.036 | -0.040 | -0.032 | 0.000 |
| Ns(household\_income)1 | 2 | -0.014 | -0.018 | -0.010 | 0.000 |
| Ns(household\_income)2 | 2 | -0.024 | -0.027 | -0.020 | 0.000 |
| Ns(household\_income)3 | 2 | -0.008 | -0.011 | -0.005 | 0.000 |
| duration\_of\_residence[10,15) | 2 | -0.018 | -0.042 | 0.006 | 0.134 |
| duration\_of\_residence[15,20) | 2 | -0.008 | -0.027 | 0.011 | 0.410 |
| duration\_of\_residence[20,Inf) | 2 | 0.000 | -0.017 | 0.017 | 0.985 |
| regionCentral Denmark Region | 2 | -0.027 | -0.030 | -0.024 | 0.000 |
| regionNorth Denmark Region | 2 | -0.036 | -0.039 | -0.032 | 0.000 |
| regionSouth Denmark Region | 2 | -0.034 | -0.037 | -0.031 | 0.000 |
| regionZealand Region | 2 | -0.014 | -0.017 | -0.011 | 0.000 |

### Monitoring: Analysis of LDL-C monitoring

| Monitoring: Analysis of LDL-C monitoring | Model | Estimate | Lower 95% CI | Upper 95% CI | P-value |
| --- | --- | --- | --- | --- | --- |
| (Intercept) | 0 | 0.131 | 0.130 | 0.133 | 0.000 |
| originMiddle East | 0 | 0.022 | 0.014 | 0.030 | 0.000 |
| originEurope | 0 | 0.023 | 0.014 | 0.032 | 0.000 |
| originTurkey | 0 | 0.023 | 0.012 | 0.034 | 0.000 |
| originFormer Yugoslavia | 0 | -0.007 | -0.018 | 0.004 | 0.216 |
| originPakistan | 0 | 0.027 | 0.013 | 0.041 | 0.000 |
| originSri Lanka | 0 | -0.039 | -0.053 | -0.025 | 0.000 |
| originSomalia | 0 | 0.053 | 0.028 | 0.077 | 0.000 |
| originVietnam | 0 | -0.006 | -0.029 | 0.018 | 0.640 |

| Monitoring: Analysis of LDL-C monitoring | Model | Estimate | Lower 95% CI | Upper 95% CI | P-value |
| --- | --- | --- | --- | --- | --- |
| (Intercept) | 1 | 0.112 | 0.109 | 0.115 | 0.000 |
| originMiddle East | 1 | 0.003 | -0.006 | 0.011 | 0.541 |
| originEurope | 1 | 0.024 | 0.016 | 0.033 | 0.000 |
| originTurkey | 1 | 0.004 | -0.007 | 0.014 | 0.492 |
| originFormer Yugoslavia | 1 | -0.014 | -0.025 | -0.003 | 0.010 |
| originPakistan | 1 | 0.016 | 0.002 | 0.030 | 0.024 |
| originSri Lanka | 1 | -0.065 | -0.080 | -0.051 | 0.000 |
| originSomalia | 1 | 0.005 | -0.020 | 0.029 | 0.721 |
| originVietnam | 1 | -0.017 | -0.041 | 0.006 | 0.141 |
| sexM | 1 | -0.004 | -0.006 | -0.001 | 0.007 |
| Ns(age)1 | 1 | -0.086 | -0.091 | -0.082 | 0.000 |
| Ns(age)2 | 1 | -0.100 | -0.103 | -0.096 | 0.000 |
| Ns(age)3 | 1 | -0.029 | -0.032 | -0.025 | 0.000 |
| Ns(diabetes\_duration)1 | 1 | 0.003 | -0.002 | 0.008 | 0.194 |
| Ns(diabetes\_duration)2 | 1 | 0.034 | 0.029 | 0.038 | 0.000 |
| Ns(diabetes\_duration)3 | 1 | -0.003 | -0.006 | 0.001 | 0.171 |
| macrovasc\_compTRUE | 1 | -0.018 | -0.021 | -0.015 | 0.000 |
| dkdTRUE | 1 | -0.033 | -0.039 | -0.028 | 0.000 |

| Monitoring: Analysis of LDL-C monitoring | Model | Estimate | Lower 95% CI | Upper 95% CI | P-value |
| --- | --- | --- | --- | --- | --- |
| (Intercept) | 2 | 0.177 | 0.154 | 0.199 | 0.000 |
| originMiddle East | 2 | -0.016 | -0.025 | -0.006 | 0.001 |
| originEurope | 2 | 0.017 | 0.008 | 0.026 | 0.000 |
| originTurkey | 2 | -0.027 | -0.038 | -0.016 | 0.000 |
| originFormer Yugoslavia | 2 | -0.025 | -0.036 | -0.014 | 0.000 |
| originPakistan | 2 | -0.037 | -0.051 | -0.023 | 0.000 |
| originSri Lanka | 2 | -0.057 | -0.071 | -0.043 | 0.000 |
| originSomalia | 2 | -0.009 | -0.034 | 0.016 | 0.481 |
| originVietnam | 2 | -0.015 | -0.038 | 0.008 | 0.196 |
| sexM | 2 | -0.004 | -0.007 | -0.001 | 0.005 |
| Ns(age)1 | 2 | -0.089 | -0.096 | -0.083 | 0.000 |
| Ns(age)2 | 2 | -0.101 | -0.108 | -0.094 | 0.000 |
| Ns(age)3 | 2 | -0.034 | -0.040 | -0.028 | 0.000 |
| Ns(diabetes\_duration)1 | 2 | 0.005 | 0.000 | 0.009 | 0.055 |
| Ns(diabetes\_duration)2 | 2 | 0.031 | 0.027 | 0.035 | 0.000 |
| Ns(diabetes\_duration)3 | 2 | -0.001 | -0.005 | 0.003 | 0.544 |
| macrovasc\_compTRUE | 2 | -0.017 | -0.020 | -0.015 | 0.000 |
| dkdTRUE | 2 | -0.033 | -0.038 | -0.028 | 0.000 |
| employment\_statusRetired | 2 | -0.019 | -0.024 | -0.013 | 0.000 |
| employment\_statusUnemployed | 2 | -0.034 | -0.039 | -0.029 | 0.000 |
| Ns(household\_income)1 | 2 | -0.020 | -0.025 | -0.015 | 0.000 |
| Ns(household\_income)2 | 2 | -0.035 | -0.039 | -0.030 | 0.000 |
| Ns(household\_income)3 | 2 | -0.018 | -0.022 | -0.014 | 0.000 |
| duration\_of\_residence[10,15) | 2 | -0.015 | -0.046 | 0.017 | 0.362 |
| duration\_of\_residence[15,20) | 2 | -0.023 | -0.048 | 0.001 | 0.059 |
| duration\_of\_residence[20,Inf) | 2 | -0.005 | -0.026 | 0.017 | 0.668 |
| regionCentral Denmark Region | 2 | -0.046 | -0.050 | -0.042 | 0.000 |
| regionNorth Denmark Region | 2 | -0.059 | -0.064 | -0.054 | 0.000 |
| regionSouth Denmark Region | 2 | -0.092 | -0.096 | -0.089 | 0.000 |
| regionZealand Region | 2 | -0.044 | -0.048 | -0.040 | 0.000 |

### Monitoring: Analysis of screening for diabetic nephropathy

| Monitoring: Analysis of screening for diabetic nephropathy | Model | Estimate | Lower 95% CI | Upper 95% CI | P-value |
| --- | --- | --- | --- | --- | --- |
| (Intercept) | 0 | 0.436 | 0.434 | 0.438 | 0.000 |
| originMiddle East | 0 | 0.048 | 0.037 | 0.059 | 0.000 |
| originEurope | 0 | 0.060 | 0.048 | 0.073 | 0.000 |
| originTurkey | 0 | 0.030 | 0.015 | 0.045 | 0.000 |
| originFormer Yugoslavia | 0 | 0.015 | -0.001 | 0.032 | 0.071 |
| originPakistan | 0 | 0.057 | 0.038 | 0.076 | 0.000 |
| originSri Lanka | 0 | -0.092 | -0.115 | -0.069 | 0.000 |
| originSomalia | 0 | 0.127 | 0.096 | 0.159 | 0.000 |
| originVietnam | 0 | -0.025 | -0.059 | 0.010 | 0.163 |

| Monitoring: Analysis of screening for diabetic nephropathy | Model | Estimate | Lower 95% CI | Upper 95% CI | P-value |
| --- | --- | --- | --- | --- | --- |
| (Intercept) | 1 | 0.429 | 0.424 | 0.433 | 0.000 |
| originMiddle East | 1 | 0.046 | 0.035 | 0.057 | 0.000 |
| originEurope | 1 | 0.053 | 0.041 | 0.065 | 0.000 |
| originTurkey | 1 | 0.022 | 0.008 | 0.037 | 0.003 |
| originFormer Yugoslavia | 1 | 0.015 | -0.001 | 0.032 | 0.067 |
| originPakistan | 1 | 0.062 | 0.044 | 0.081 | 0.000 |
| originSri Lanka | 1 | -0.090 | -0.113 | -0.067 | 0.000 |
| originSomalia | 1 | 0.097 | 0.066 | 0.129 | 0.000 |
| originVietnam | 1 | -0.020 | -0.054 | 0.014 | 0.251 |
| sexM | 1 | -0.061 | -0.065 | -0.057 | 0.000 |
| Ns(age)1 | 1 | -0.073 | -0.080 | -0.067 | 0.000 |
| Ns(age)2 | 1 | -0.059 | -0.065 | -0.054 | 0.000 |
| Ns(age)3 | 1 | 0.005 | 0.000 | 0.011 | 0.058 |
| Ns(diabetes\_duration)1 | 1 | -0.029 | -0.036 | -0.022 | 0.000 |
| Ns(diabetes\_duration)2 | 1 | -0.092 | -0.098 | -0.085 | 0.000 |
| Ns(diabetes\_duration)3 | 1 | -0.036 | -0.041 | -0.031 | 0.000 |
| macrovasc\_compTRUE | 1 | 0.014 | 0.010 | 0.018 | 0.000 |
| dkdTRUE | 1 | -0.340 | -0.346 | -0.335 | 0.000 |

| Monitoring: Analysis of screening for diabetic nephropathy | Model | Estimate | Lower 95% CI | Upper 95% CI | P-value |
| --- | --- | --- | --- | --- | --- |
| (Intercept) | 2 | 0.504 | 0.475 | 0.532 | 0.000 |
| originMiddle East | 2 | 0.011 | -0.002 | 0.023 | 0.086 |
| originEurope | 2 | 0.039 | 0.027 | 0.052 | 0.000 |
| originTurkey | 2 | -0.030 | -0.044 | -0.015 | 0.000 |
| originFormer Yugoslavia | 2 | -0.014 | -0.031 | 0.002 | 0.082 |
| originPakistan | 2 | -0.033 | -0.052 | -0.014 | 0.001 |
| originSri Lanka | 2 | -0.045 | -0.067 | -0.023 | 0.000 |
| originSomalia | 2 | 0.080 | 0.048 | 0.113 | 0.000 |
| originVietnam | 2 | -0.004 | -0.038 | 0.029 | 0.795 |
| sexM | 2 | -0.058 | -0.062 | -0.054 | 0.000 |
| Ns(age)1 | 2 | -0.068 | -0.078 | -0.058 | 0.000 |
| Ns(age)2 | 2 | -0.055 | -0.064 | -0.045 | 0.000 |
| Ns(age)3 | 2 | 0.009 | 0.000 | 0.018 | 0.043 |
| Ns(diabetes\_duration)1 | 2 | -0.031 | -0.037 | -0.024 | 0.000 |
| Ns(diabetes\_duration)2 | 2 | -0.097 | -0.103 | -0.091 | 0.000 |
| Ns(diabetes\_duration)3 | 2 | -0.039 | -0.044 | -0.034 | 0.000 |
| macrovasc\_compTRUE | 2 | 0.014 | 0.010 | 0.019 | 0.000 |
| dkdTRUE | 2 | -0.343 | -0.348 | -0.337 | 0.000 |
| employment\_statusRetired | 2 | -0.004 | -0.012 | 0.004 | 0.358 |
| employment\_statusUnemployed | 2 | 0.020 | 0.013 | 0.026 | 0.000 |
| Ns(household\_income)1 | 2 | -0.028 | -0.035 | -0.020 | 0.000 |
| Ns(household\_income)2 | 2 | -0.028 | -0.034 | -0.022 | 0.000 |
| Ns(household\_income)3 | 2 | -0.019 | -0.025 | -0.013 | 0.000 |
| duration\_of\_residence[10,15) | 2 | 0.020 | -0.020 | 0.060 | 0.330 |
| duration\_of\_residence[15,20) | 2 | 0.015 | -0.016 | 0.047 | 0.341 |
| duration\_of\_residence[20,Inf) | 2 | 0.019 | -0.008 | 0.046 | 0.168 |
| regionCentral Denmark Region | 2 | -0.228 | -0.233 | -0.222 | 0.000 |
| regionNorth Denmark Region | 2 | -0.020 | -0.027 | -0.013 | 0.000 |
| regionSouth Denmark Region | 2 | -0.140 | -0.146 | -0.135 | 0.000 |
| regionZealand Region | 2 | -0.061 | -0.067 | -0.055 | 0.000 |

### Monitoring: Analysis of screening for diabetic retinopathy

| Monitoring: Analysis of screening for diabetic retinopathy | Model | Estimate | Lower 95% CI | Upper 95% CI | P-value |
| --- | --- | --- | --- | --- | --- |
| (Intercept) | 0 | 0.432 | 0.430 | 0.434 | 0.000 |
| originMiddle East | 0 | 0.074 | 0.063 | 0.085 | 0.000 |
| originEurope | 0 | 0.080 | 0.068 | 0.092 | 0.000 |
| originTurkey | 0 | 0.064 | 0.049 | 0.078 | 0.000 |
| originFormer Yugoslavia | 0 | 0.089 | 0.072 | 0.106 | 0.000 |
| originPakistan | 0 | 0.157 | 0.139 | 0.176 | 0.000 |
| originSri Lanka | 0 | -0.120 | -0.142 | -0.097 | 0.000 |
| originSomalia | 0 | 0.127 | 0.096 | 0.159 | 0.000 |
| originVietnam | 0 | -0.002 | -0.037 | 0.032 | 0.889 |

| Monitoring: Analysis of screening for diabetic retinopathy | Model | Estimate | Lower 95% CI | Upper 95% CI | P-value |
| --- | --- | --- | --- | --- | --- |
| (Intercept) | 1 | 0.282 | 0.278 | 0.286 | 0.000 |
| originMiddle East | 1 | 0.041 | 0.030 | 0.053 | 0.000 |
| originEurope | 1 | 0.075 | 0.063 | 0.087 | 0.000 |
| originTurkey | 1 | 0.036 | 0.021 | 0.050 | 0.000 |
| originFormer Yugoslavia | 1 | 0.075 | 0.059 | 0.092 | 0.000 |
| originPakistan | 1 | 0.152 | 0.134 | 0.170 | 0.000 |
| originSri Lanka | 1 | -0.150 | -0.172 | -0.128 | 0.000 |
| originSomalia | 1 | 0.053 | 0.021 | 0.085 | 0.001 |
| originVietnam | 1 | -0.023 | -0.057 | 0.011 | 0.185 |
| sexM | 1 | 0.010 | 0.006 | 0.013 | 0.000 |
| Ns(age)1 | 1 | -0.167 | -0.173 | -0.160 | 0.000 |
| Ns(age)2 | 1 | -0.170 | -0.175 | -0.164 | 0.000 |
| Ns(age)3 | 1 | -0.048 | -0.053 | -0.042 | 0.000 |
| Ns(diabetes\_duration)1 | 1 | -0.095 | -0.102 | -0.088 | 0.000 |
| Ns(diabetes\_duration)2 | 1 | -0.265 | -0.271 | -0.258 | 0.000 |
| Ns(diabetes\_duration)3 | 1 | -0.068 | -0.073 | -0.062 | 0.000 |
| macrovasc\_compTRUE | 1 | 0.053 | 0.049 | 0.057 | 0.000 |
| dkdTRUE | 1 | 0.002 | -0.007 | 0.010 | 0.708 |

| Monitoring: Analysis of screening for diabetic retinopathy | Model | Estimate | Lower 95% CI | Upper 95% CI | P-value |
| --- | --- | --- | --- | --- | --- |
| (Intercept) | 2 | 0.361 | 0.333 | 0.389 | 0.000 |
| originMiddle East | 2 | -0.025 | -0.037 | -0.012 | 0.000 |
| originEurope | 2 | 0.060 | 0.047 | 0.072 | 0.000 |
| originTurkey | 2 | -0.050 | -0.065 | -0.036 | 0.000 |
| originFormer Yugoslavia | 2 | 0.032 | 0.016 | 0.048 | 0.000 |
| originPakistan | 2 | 0.024 | 0.005 | 0.043 | 0.012 |
| originSri Lanka | 2 | -0.136 | -0.158 | -0.114 | 0.000 |
| originSomalia | 2 | -0.007 | -0.040 | 0.025 | 0.655 |
| originVietnam | 2 | -0.025 | -0.059 | 0.009 | 0.145 |
| sexM | 2 | 0.013 | 0.009 | 0.017 | 0.000 |
| Ns(age)1 | 2 | -0.137 | -0.147 | -0.127 | 0.000 |
| Ns(age)2 | 2 | -0.137 | -0.146 | -0.127 | 0.000 |
| Ns(age)3 | 2 | -0.026 | -0.035 | -0.017 | 0.000 |
| Ns(diabetes\_duration)1 | 2 | -0.094 | -0.101 | -0.087 | 0.000 |
| Ns(diabetes\_duration)2 | 2 | -0.273 | -0.279 | -0.267 | 0.000 |
| Ns(diabetes\_duration)3 | 2 | -0.067 | -0.072 | -0.062 | 0.000 |
| macrovasc\_compTRUE | 2 | 0.050 | 0.046 | 0.055 | 0.000 |
| dkdTRUE | 2 | -0.002 | -0.010 | 0.006 | 0.630 |
| employment\_statusRetired | 2 | -0.042 | -0.050 | -0.034 | 0.000 |
| employment\_statusUnemployed | 2 | 0.006 | 0.000 | 0.013 | 0.057 |
| Ns(household\_income)1 | 2 | -0.058 | -0.065 | -0.051 | 0.000 |
| Ns(household\_income)2 | 2 | -0.089 | -0.095 | -0.083 | 0.000 |
| Ns(household\_income)3 | 2 | -0.040 | -0.045 | -0.034 | 0.000 |
| duration\_of\_residence[10,15) | 2 | 0.045 | 0.005 | 0.085 | 0.026 |
| duration\_of\_residence[15,20) | 2 | 0.020 | -0.011 | 0.051 | 0.201 |
| duration\_of\_residence[20,Inf) | 2 | 0.044 | 0.018 | 0.070 | 0.001 |
| regionCentral Denmark Region | 2 | -0.136 | -0.141 | -0.130 | 0.000 |
| regionNorth Denmark Region | 2 | -0.121 | -0.128 | -0.114 | 0.000 |
| regionSouth Denmark Region | 2 | -0.211 | -0.216 | -0.206 | 0.000 |
| regionZealand Region | 2 | -0.081 | -0.086 | -0.075 | 0.000 |

### Monitoring: Analysis of screening for diabetic foot disease

| Monitoring: Analysis of screening for diabetic foot disease | Model | Estimate | Lower 95% CI | Upper 95% CI | P-value |
| --- | --- | --- | --- | --- | --- |
| (Intercept) | 0 | 0.570 | 0.568 | 0.572 | 0 |
| originMiddle East | 0 | 0.246 | 0.237 | 0.255 | 0 |
| originEurope | 0 | 0.082 | 0.070 | 0.094 | 0 |
| originTurkey | 0 | 0.237 | 0.225 | 0.249 | 0 |
| originFormer Yugoslavia | 0 | 0.231 | 0.218 | 0.245 | 0 |
| originPakistan | 0 | 0.232 | 0.217 | 0.247 | 0 |
| originSri Lanka | 0 | 0.091 | 0.068 | 0.114 | 0 |
| originSomalia | 0 | 0.304 | 0.283 | 0.325 | 0 |
| originVietnam | 0 | 0.292 | 0.268 | 0.316 | 0 |

| Monitoring: Analysis of screening for diabetic foot disease | Model | Estimate | Lower 95% CI | Upper 95% CI | P-value |
| --- | --- | --- | --- | --- | --- |
| (Intercept) | 1 | 0.495 | 0.490 | 0.499 | 0.000 |
| originMiddle East | 1 | 0.201 | 0.192 | 0.210 | 0.000 |
| originEurope | 1 | 0.073 | 0.062 | 0.084 | 0.000 |
| originTurkey | 1 | 0.198 | 0.186 | 0.210 | 0.000 |
| originFormer Yugoslavia | 1 | 0.208 | 0.195 | 0.222 | 0.000 |
| originPakistan | 1 | 0.221 | 0.205 | 0.236 | 0.000 |
| originSri Lanka | 1 | 0.052 | 0.029 | 0.074 | 0.000 |
| originSomalia | 1 | 0.213 | 0.191 | 0.235 | 0.000 |
| originVietnam | 1 | 0.263 | 0.238 | 0.287 | 0.000 |
| sexM | 1 | 0.044 | 0.041 | 0.048 | 0.000 |
| Ns(age)1 | 1 | -0.128 | -0.135 | -0.122 | 0.000 |
| Ns(age)2 | 1 | -0.165 | -0.170 | -0.160 | 0.000 |
| Ns(age)3 | 1 | -0.084 | -0.089 | -0.078 | 0.000 |
| Ns(diabetes\_duration)1 | 1 | -0.060 | -0.067 | -0.054 | 0.000 |
| Ns(diabetes\_duration)2 | 1 | -0.236 | -0.242 | -0.230 | 0.000 |
| Ns(diabetes\_duration)3 | 1 | -0.097 | -0.102 | -0.092 | 0.000 |
| macrovasc\_compTRUE | 1 | -0.004 | -0.009 | 0.000 | 0.043 |
| dkdTRUE | 1 | -0.048 | -0.056 | -0.040 | 0.000 |

| Monitoring: Analysis of screening for diabetic foot disease | Model | Estimate | Lower 95% CI | Upper 95% CI | P-value |
| --- | --- | --- | --- | --- | --- |
| (Intercept) | 2 | 0.538 | 0.517 | 0.559 | 0.000 |
| originMiddle East | 2 | 0.158 | 0.147 | 0.168 | 0.000 |
| originEurope | 2 | 0.061 | 0.049 | 0.073 | 0.000 |
| originTurkey | 2 | 0.158 | 0.146 | 0.170 | 0.000 |
| originFormer Yugoslavia | 2 | 0.180 | 0.166 | 0.193 | 0.000 |
| originPakistan | 2 | 0.163 | 0.147 | 0.178 | 0.000 |
| originSri Lanka | 2 | 0.035 | 0.013 | 0.058 | 0.002 |
| originSomalia | 2 | 0.155 | 0.132 | 0.178 | 0.000 |
| originVietnam | 2 | 0.233 | 0.208 | 0.257 | 0.000 |
| sexM | 2 | 0.044 | 0.040 | 0.047 | 0.000 |
| Ns(age)1 | 2 | -0.068 | -0.078 | -0.057 | 0.000 |
| Ns(age)2 | 2 | -0.097 | -0.107 | -0.088 | 0.000 |
| Ns(age)3 | 2 | -0.036 | -0.045 | -0.027 | 0.000 |
| Ns(diabetes\_duration)1 | 2 | -0.060 | -0.067 | -0.053 | 0.000 |
| Ns(diabetes\_duration)2 | 2 | -0.237 | -0.243 | -0.231 | 0.000 |
| Ns(diabetes\_duration)3 | 2 | -0.096 | -0.102 | -0.091 | 0.000 |
| macrovasc\_compTRUE | 2 | -0.004 | -0.008 | 0.000 | 0.064 |
| dkdTRUE | 2 | -0.048 | -0.056 | -0.040 | 0.000 |
| employment\_statusRetired | 2 | -0.087 | -0.095 | -0.079 | 0.000 |
| employment\_statusUnemployed | 2 | -0.035 | -0.041 | -0.028 | 0.000 |
| Ns(household\_income)1 | 2 | -0.046 | -0.053 | -0.039 | 0.000 |
| Ns(household\_income)2 | 2 | -0.094 | -0.100 | -0.088 | 0.000 |
| Ns(household\_income)3 | 2 | -0.030 | -0.036 | -0.024 | 0.000 |
| duration\_of\_residence[10,15) | 2 | 0.048 | 0.019 | 0.077 | 0.001 |
| duration\_of\_residence[15,20) | 2 | 0.038 | 0.016 | 0.061 | 0.001 |
| duration\_of\_residence[20,Inf) | 2 | 0.029 | 0.010 | 0.048 | 0.003 |
| regionCentral Denmark Region | 2 | -0.015 | -0.021 | -0.010 | 0.000 |
| regionNorth Denmark Region | 2 | -0.019 | -0.026 | -0.013 | 0.000 |
| regionSouth Denmark Region | 2 | -0.032 | -0.037 | -0.027 | 0.000 |
| regionZealand Region | 2 | -0.042 | -0.048 | -0.036 | 0.000 |

### Biomarker levels: Analysis of HbA1c levels

| Biomarker levels: Analysis of HbA1c levels | Model | Estimate | Lower 95% CI | Upper 95% CI | P-value |
| --- | --- | --- | --- | --- | --- |
| (Intercept) | 0 | 0.372 | 0.370 | 0.374 | 0.000 |
| originMiddle East | 0 | 0.100 | 0.089 | 0.111 | 0.000 |
| originEurope | 0 | 0.009 | -0.003 | 0.022 | 0.127 |
| originTurkey | 0 | 0.164 | 0.149 | 0.179 | 0.000 |
| originFormer Yugoslavia | 0 | 0.121 | 0.104 | 0.138 | 0.000 |
| originPakistan | 0 | 0.172 | 0.153 | 0.192 | 0.000 |
| originSri Lanka | 0 | 0.142 | 0.118 | 0.166 | 0.000 |
| originSomalia | 0 | 0.124 | 0.092 | 0.156 | 0.000 |
| originVietnam | 0 | -0.008 | -0.042 | 0.027 | 0.653 |

| Biomarker levels: Analysis of HbA1c levels | Model | Estimate | Lower 95% CI | Upper 95% CI | P-value |
| --- | --- | --- | --- | --- | --- |
| (Intercept) | 1 | 0.288 | 0.284 | 0.292 | 0.000 |
| originMiddle East | 1 | 0.045 | 0.034 | 0.055 | 0.000 |
| originEurope | 1 | 0.030 | 0.019 | 0.042 | 0.000 |
| originTurkey | 1 | 0.116 | 0.102 | 0.130 | 0.000 |
| originFormer Yugoslavia | 1 | 0.093 | 0.077 | 0.109 | 0.000 |
| originPakistan | 1 | 0.101 | 0.084 | 0.119 | 0.000 |
| originSri Lanka | 1 | 0.045 | 0.022 | 0.067 | 0.000 |
| originSomalia | 1 | 0.058 | 0.028 | 0.088 | 0.000 |
| originVietnam | 1 | -0.026 | -0.058 | 0.007 | 0.128 |
| sexM | 1 | 0.055 | 0.052 | 0.059 | 0.000 |
| Ns(age)1 | 1 | -0.141 | -0.148 | -0.135 | 0.000 |
| Ns(age)2 | 1 | -0.191 | -0.196 | -0.186 | 0.000 |
| Ns(age)3 | 1 | -0.147 | -0.152 | -0.142 | 0.000 |
| Ns(diabetes\_duration)1 | 1 | 0.188 | 0.181 | 0.194 | 0.000 |
| Ns(diabetes\_duration)2 | 1 | 0.322 | 0.316 | 0.328 | 0.000 |
| Ns(diabetes\_duration)3 | 1 | 0.268 | 0.262 | 0.273 | 0.000 |
| macrovasc\_compTRUE | 1 | 0.006 | 0.002 | 0.010 | 0.003 |
| dkdTRUE | 1 | 0.066 | 0.058 | 0.074 | 0.000 |

| Biomarker levels: Analysis of HbA1c levels | Model | Estimate | Lower 95% CI | Upper 95% CI | P-value |
| --- | --- | --- | --- | --- | --- |
| (Intercept) | 2 | 0.416 | 0.387 | 0.445 | 0.000 |
| originMiddle East | 2 | 0.027 | 0.015 | 0.039 | 0.000 |
| originEurope | 2 | 0.016 | 0.004 | 0.028 | 0.007 |
| originTurkey | 2 | 0.108 | 0.094 | 0.122 | 0.000 |
| originFormer Yugoslavia | 2 | 0.081 | 0.065 | 0.097 | 0.000 |
| originPakistan | 2 | 0.085 | 0.067 | 0.103 | 0.000 |
| originSri Lanka | 2 | 0.034 | 0.011 | 0.056 | 0.004 |
| originSomalia | 2 | 0.032 | 0.001 | 0.063 | 0.042 |
| originVietnam | 2 | -0.050 | -0.083 | -0.018 | 0.003 |
| sexM | 2 | 0.054 | 0.051 | 0.058 | 0.000 |
| Ns(age)1 | 2 | -0.114 | -0.124 | -0.105 | 0.000 |
| Ns(age)2 | 2 | -0.157 | -0.167 | -0.148 | 0.000 |
| Ns(age)3 | 2 | -0.127 | -0.136 | -0.119 | 0.000 |
| Ns(diabetes\_duration)1 | 2 | 0.188 | 0.181 | 0.194 | 0.000 |
| Ns(diabetes\_duration)2 | 2 | 0.326 | 0.320 | 0.331 | 0.000 |
| Ns(diabetes\_duration)3 | 2 | 0.267 | 0.262 | 0.272 | 0.000 |
| macrovasc\_compTRUE | 2 | 0.007 | 0.003 | 0.011 | 0.000 |
| dkdTRUE | 2 | 0.067 | 0.059 | 0.075 | 0.000 |
| employment\_statusRetired | 2 | -0.055 | -0.063 | -0.047 | 0.000 |
| employment\_statusUnemployed | 2 | -0.041 | -0.047 | -0.034 | 0.000 |
| Ns(household\_income)1 | 2 | -0.015 | -0.022 | -0.008 | 0.000 |
| Ns(household\_income)2 | 2 | -0.049 | -0.055 | -0.043 | 0.000 |
| Ns(household\_income)3 | 2 | -0.024 | -0.029 | -0.019 | 0.000 |
| duration\_of\_residence[10,15) | 2 | -0.088 | -0.128 | -0.048 | 0.000 |
| duration\_of\_residence[15,20) | 2 | -0.089 | -0.121 | -0.058 | 0.000 |
| duration\_of\_residence[20,Inf) | 2 | -0.094 | -0.122 | -0.067 | 0.000 |
| regionCentral Denmark Region | 2 | -0.009 | -0.014 | -0.004 | 0.001 |
| regionNorth Denmark Region | 2 | 0.043 | 0.037 | 0.050 | 0.000 |
| regionSouth Denmark Region | 2 | 0.044 | 0.039 | 0.049 | 0.000 |
| regionZealand Region | 2 | -0.007 | -0.012 | -0.001 | 0.016 |

### Biomarker levels: Analysis of LDL-C levels

| Biomarker levels: Analysis of LDL-C levels | Model | Estimate | Lower 95% CI | Upper 95% CI | P-value |
| --- | --- | --- | --- | --- | --- |
| (Intercept) | 0 | 0.283 | 0.281 | 0.285 | 0.000 |
| originMiddle East | 0 | 0.049 | 0.038 | 0.059 | 0.000 |
| originEurope | 0 | 0.050 | 0.038 | 0.062 | 0.000 |
| originTurkey | 0 | 0.042 | 0.028 | 0.056 | 0.000 |
| originFormer Yugoslavia | 0 | 0.023 | 0.008 | 0.039 | 0.003 |
| originPakistan | 0 | 0.039 | 0.021 | 0.057 | 0.000 |
| originSri Lanka | 0 | 0.003 | -0.019 | 0.025 | 0.820 |
| originSomalia | 0 | 0.221 | 0.189 | 0.253 | 0.000 |
| originVietnam | 0 | -0.023 | -0.054 | 0.008 | 0.148 |

| Biomarker levels: Analysis of LDL-C levels | Model | Estimate | Lower 95% CI | Upper 95% CI | P-value |
| --- | --- | --- | --- | --- | --- |
| (Intercept) | 1 | 0.290 | 0.286 | 0.295 | 0.000 |
| originMiddle East | 1 | 0.027 | 0.017 | 0.038 | 0.000 |
| originEurope | 1 | 0.043 | 0.032 | 0.055 | 0.000 |
| originTurkey | 1 | 0.015 | 0.001 | 0.028 | 0.036 |
| originFormer Yugoslavia | 1 | 0.010 | -0.005 | 0.026 | 0.177 |
| originPakistan | 1 | 0.036 | 0.019 | 0.053 | 0.000 |
| originSri Lanka | 1 | -0.024 | -0.046 | -0.002 | 0.029 |
| originSomalia | 1 | 0.153 | 0.121 | 0.185 | 0.000 |
| originVietnam | 1 | -0.052 | -0.082 | -0.021 | 0.001 |
| sexM | 1 | -0.066 | -0.070 | -0.063 | 0.000 |
| Ns(age)1 | 1 | -0.090 | -0.096 | -0.084 | 0.000 |
| Ns(age)2 | 1 | -0.102 | -0.107 | -0.097 | 0.000 |
| Ns(age)3 | 1 | -0.037 | -0.042 | -0.032 | 0.000 |
| Ns(diabetes\_duration)1 | 1 | -0.072 | -0.078 | -0.066 | 0.000 |
| Ns(diabetes\_duration)2 | 1 | -0.169 | -0.175 | -0.163 | 0.000 |
| Ns(diabetes\_duration)3 | 1 | -0.070 | -0.075 | -0.065 | 0.000 |
| macrovasc\_compTRUE | 1 | -0.083 | -0.086 | -0.079 | 0.000 |
| dkdTRUE | 1 | -0.004 | -0.012 | 0.003 | 0.245 |

| Biomarker levels: Analysis of LDL-C levels | Model | Estimate | Lower 95% CI | Upper 95% CI | P-value |
| --- | --- | --- | --- | --- | --- |
| (Intercept) | 2 | 0.265 | 0.237 | 0.293 | 0.000 |
| originMiddle East | 2 | 0.027 | 0.015 | 0.039 | 0.000 |
| originEurope | 2 | 0.043 | 0.031 | 0.055 | 0.000 |
| originTurkey | 2 | 0.005 | -0.009 | 0.019 | 0.453 |
| originFormer Yugoslavia | 2 | 0.006 | -0.010 | 0.021 | 0.473 |
| originPakistan | 2 | 0.020 | 0.002 | 0.038 | 0.029 |
| originSri Lanka | 2 | -0.019 | -0.041 | 0.003 | 0.085 |
| originSomalia | 2 | 0.154 | 0.122 | 0.187 | 0.000 |
| originVietnam | 2 | -0.051 | -0.082 | -0.021 | 0.001 |
| sexM | 2 | -0.068 | -0.072 | -0.064 | 0.000 |
| Ns(age)1 | 2 | -0.075 | -0.084 | -0.065 | 0.000 |
| Ns(age)2 | 2 | -0.086 | -0.095 | -0.077 | 0.000 |
| Ns(age)3 | 2 | -0.024 | -0.032 | -0.016 | 0.000 |
| Ns(diabetes\_duration)1 | 2 | -0.073 | -0.079 | -0.067 | 0.000 |
| Ns(diabetes\_duration)2 | 2 | -0.169 | -0.175 | -0.163 | 0.000 |
| Ns(diabetes\_duration)3 | 2 | -0.071 | -0.076 | -0.066 | 0.000 |
| macrovasc\_compTRUE | 2 | -0.082 | -0.085 | -0.078 | 0.000 |
| dkdTRUE | 2 | -0.003 | -0.011 | 0.004 | 0.364 |
| employment\_statusRetired | 2 | -0.020 | -0.028 | -0.012 | 0.000 |
| employment\_statusUnemployed | 2 | -0.013 | -0.019 | -0.006 | 0.000 |
| Ns(household\_income)1 | 2 | -0.005 | -0.012 | 0.002 | 0.153 |
| Ns(household\_income)2 | 2 | -0.015 | -0.021 | -0.009 | 0.000 |
| Ns(household\_income)3 | 2 | 0.004 | -0.002 | 0.009 | 0.179 |
| duration\_of\_residence[10,15) | 2 | 0.046 | 0.006 | 0.086 | 0.024 |
| duration\_of\_residence[15,20) | 2 | 0.029 | -0.002 | 0.060 | 0.071 |
| duration\_of\_residence[20,Inf) | 2 | 0.049 | 0.022 | 0.076 | 0.000 |
| regionCentral Denmark Region | 2 | -0.027 | -0.032 | -0.022 | 0.000 |
| regionNorth Denmark Region | 2 | -0.043 | -0.049 | -0.037 | 0.000 |
| regionSouth Denmark Region | 2 | -0.005 | -0.010 | 0.000 | 0.073 |
| regionZealand Region | 2 | -0.013 | -0.018 | -0.007 | 0.000 |

### Pharmacological treatment: Analysis of glucose-lowering drugs

| Pharmacological treatment: Analysis of glucose-lowering drugs | Model | Estimate | Lower 95% CI | Upper 95% CI | P-value |
| --- | --- | --- | --- | --- | --- |
| (Intercept) | 0 | 0.069 | 0.068 | 0.070 | 0.000 |
| originMiddle East | 0 | -0.005 | -0.012 | 0.002 | 0.131 |
| originEurope | 0 | 0.031 | 0.021 | 0.041 | 0.000 |
| originTurkey | 0 | -0.018 | -0.026 | -0.010 | 0.000 |
| originFormer Yugoslavia | 0 | -0.011 | -0.021 | -0.002 | 0.021 |
| originPakistan | 0 | 0.010 | -0.002 | 0.022 | 0.117 |
| originSri Lanka | 0 | -0.027 | -0.039 | -0.016 | 0.000 |
| originSomalia | 0 | 0.047 | 0.021 | 0.072 | 0.000 |
| originVietnam | 0 | 0.003 | -0.021 | 0.027 | 0.792 |

| Pharmacological treatment: Analysis of glucose-lowering drugs | Model | Estimate | Lower 95% CI | Upper 95% CI | P-value |
| --- | --- | --- | --- | --- | --- |
| (Intercept) | 1 | 0.022 | 0.020 | 0.025 | 0.000 |
| originMiddle East | 1 | 0.008 | 0.001 | 0.015 | 0.017 |
| originEurope | 1 | 0.020 | 0.010 | 0.029 | 0.000 |
| originTurkey | 1 | -0.003 | -0.011 | 0.004 | 0.379 |
| originFormer Yugoslavia | 1 | -0.004 | -0.014 | 0.005 | 0.351 |
| originPakistan | 1 | 0.035 | 0.023 | 0.047 | 0.000 |
| originSri Lanka | 1 | 0.003 | -0.008 | 0.014 | 0.610 |
| originSomalia | 1 | 0.056 | 0.031 | 0.081 | 0.000 |
| originVietnam | 1 | 0.005 | -0.018 | 0.027 | 0.687 |
| sexM | 1 | -0.012 | -0.014 | -0.009 | 0.000 |
| Ns(age)1 | 1 | 0.007 | 0.002 | 0.011 | 0.004 |
| Ns(age)2 | 1 | 0.036 | 0.032 | 0.040 | 0.000 |
| Ns(age)3 | 1 | 0.052 | 0.049 | 0.056 | 0.000 |
| Ns(diabetes\_duration)1 | 1 | -0.079 | -0.083 | -0.075 | 0.000 |
| Ns(diabetes\_duration)2 | 1 | -0.234 | -0.241 | -0.227 | 0.000 |
| Ns(diabetes\_duration)3 | 1 | -0.071 | -0.074 | -0.067 | 0.000 |
| macrovasc\_compTRUE | 1 | 0.008 | 0.005 | 0.010 | 0.000 |
| dkdTRUE | 1 | -0.008 | -0.012 | -0.004 | 0.000 |

| Pharmacological treatment: Analysis of glucose-lowering drugs | Model | Estimate | Lower 95% CI | Upper 95% CI | P-value |
| --- | --- | --- | --- | --- | --- |
| (Intercept) | 2 | 0.014 | -0.008 | 0.037 | 0.204 |
| originMiddle East | 2 | 0.001 | -0.007 | 0.008 | 0.878 |
| originEurope | 2 | 0.018 | 0.008 | 0.028 | 0.000 |
| originTurkey | 2 | -0.017 | -0.025 | -0.009 | 0.000 |
| originFormer Yugoslavia | 2 | -0.011 | -0.021 | -0.002 | 0.018 |
| originPakistan | 2 | 0.014 | 0.002 | 0.026 | 0.020 |
| originSri Lanka | 2 | 0.004 | -0.007 | 0.016 | 0.455 |
| originSomalia | 2 | 0.048 | 0.022 | 0.073 | 0.000 |
| originVietnam | 2 | 0.004 | -0.019 | 0.027 | 0.720 |
| sexM | 2 | -0.011 | -0.014 | -0.008 | 0.000 |
| Ns(age)1 | 2 | -0.003 | -0.010 | 0.004 | 0.362 |
| Ns(age)2 | 2 | 0.026 | 0.019 | 0.032 | 0.000 |
| Ns(age)3 | 2 | 0.043 | 0.037 | 0.049 | 0.000 |
| Ns(diabetes\_duration)1 | 2 | -0.079 | -0.082 | -0.075 | 0.000 |
| Ns(diabetes\_duration)2 | 2 | -0.235 | -0.241 | -0.228 | 0.000 |
| Ns(diabetes\_duration)3 | 2 | -0.071 | -0.074 | -0.067 | 0.000 |
| macrovasc\_compTRUE | 2 | 0.007 | 0.004 | 0.010 | 0.000 |
| dkdTRUE | 2 | -0.008 | -0.013 | -0.004 | 0.000 |
| employment\_statusRetired | 2 | 0.009 | 0.004 | 0.014 | 0.001 |
| employment\_statusUnemployed | 2 | 0.001 | -0.003 | 0.006 | 0.537 |
| Ns(household\_income)1 | 2 | -0.001 | -0.006 | 0.004 | 0.813 |
| Ns(household\_income)2 | 2 | -0.009 | -0.014 | -0.005 | 0.000 |
| Ns(household\_income)3 | 2 | 0.000 | -0.004 | 0.004 | 0.978 |
| duration\_of\_residence[10,15) | 2 | 0.011 | -0.019 | 0.041 | 0.472 |
| duration\_of\_residence[15,20) | 2 | 0.008 | -0.016 | 0.031 | 0.511 |
| duration\_of\_residence[20,Inf) | 2 | 0.019 | -0.002 | 0.041 | 0.081 |
| regionCentral Denmark Region | 2 | -0.019 | -0.023 | -0.015 | 0.000 |
| regionNorth Denmark Region | 2 | -0.036 | -0.041 | -0.032 | 0.000 |
| regionSouth Denmark Region | 2 | -0.027 | -0.031 | -0.023 | 0.000 |
| regionZealand Region | 2 | -0.025 | -0.029 | -0.021 | 0.000 |

### Pharmacological treatment: Analysis of lipid-lowering drugs

| Pharmacological treatment: Analysis of lipid-lowering drugs | Model | Estimate | Lower 95% CI | Upper 95% CI | P-value |
| --- | --- | --- | --- | --- | --- |
| (Intercept) | 0 | 0.349 | 0.346 | 0.352 | 0.000 |
| originMiddle East | 0 | 0.003 | -0.012 | 0.017 | 0.736 |
| originEurope | 0 | 0.048 | 0.031 | 0.064 | 0.000 |
| originTurkey | 0 | -0.032 | -0.051 | -0.013 | 0.001 |
| originFormer Yugoslavia | 0 | -0.065 | -0.085 | -0.044 | 0.000 |
| originPakistan | 0 | -0.033 | -0.057 | -0.009 | 0.007 |
| originSri Lanka | 0 | -0.036 | -0.068 | -0.004 | 0.028 |
| originSomalia | 0 | 0.223 | 0.181 | 0.265 | 0.000 |
| originVietnam | 0 | -0.053 | -0.101 | -0.006 | 0.028 |

| Pharmacological treatment: Analysis of lipid-lowering drugs | Model | Estimate | Lower 95% CI | Upper 95% CI | P-value |
| --- | --- | --- | --- | --- | --- |
| (Intercept) | 1 | 0.513 | 0.507 | 0.520 | 0.000 |
| originMiddle East | 1 | -0.004 | -0.017 | 0.010 | 0.609 |
| originEurope | 1 | 0.034 | 0.019 | 0.049 | 0.000 |
| originTurkey | 1 | -0.041 | -0.058 | -0.023 | 0.000 |
| originFormer Yugoslavia | 1 | -0.050 | -0.069 | -0.031 | 0.000 |
| originPakistan | 1 | -0.012 | -0.034 | 0.010 | 0.268 |
| originSri Lanka | 1 | -0.051 | -0.080 | -0.021 | 0.001 |
| originSomalia | 1 | 0.083 | 0.042 | 0.125 | 0.000 |
| originVietnam | 1 | -0.084 | -0.129 | -0.038 | 0.000 |
| sexM | 1 | -0.033 | -0.037 | -0.028 | 0.000 |
| Ns(age)1 | 1 | -0.063 | -0.071 | -0.055 | 0.000 |
| Ns(age)2 | 1 | -0.047 | -0.055 | -0.040 | 0.000 |
| Ns(age)3 | 1 | 0.061 | 0.054 | 0.068 | 0.000 |
| Ns(diabetes\_duration)1 | 1 | -0.024 | -0.033 | -0.016 | 0.000 |
| Ns(diabetes\_duration)2 | 1 | -0.042 | -0.050 | -0.034 | 0.000 |
| Ns(diabetes\_duration)3 | 1 | -0.025 | -0.032 | -0.018 | 0.000 |
| macrovasc\_compTRUE | 1 | -0.369 | -0.374 | -0.364 | 0.000 |
| dkdTRUE | 1 | -0.179 | -0.187 | -0.171 | 0.000 |

| Pharmacological treatment: Analysis of lipid-lowering drugs | Model | Estimate | Lower 95% CI | Upper 95% CI | P-value |
| --- | --- | --- | --- | --- | --- |
| (Intercept) | 2 | 0.470 | 0.430 | 0.511 | 0.000 |
| originMiddle East | 2 | -0.008 | -0.024 | 0.007 | 0.281 |
| originEurope | 2 | 0.036 | 0.021 | 0.052 | 0.000 |
| originTurkey | 2 | -0.059 | -0.077 | -0.041 | 0.000 |
| originFormer Yugoslavia | 2 | -0.055 | -0.075 | -0.036 | 0.000 |
| originPakistan | 2 | -0.035 | -0.058 | -0.013 | 0.002 |
| originSri Lanka | 2 | -0.037 | -0.067 | -0.007 | 0.016 |
| originSomalia | 2 | 0.085 | 0.043 | 0.128 | 0.000 |
| originVietnam | 2 | -0.073 | -0.118 | -0.028 | 0.001 |
| sexM | 2 | -0.031 | -0.036 | -0.026 | 0.000 |
| Ns(age)1 | 2 | -0.077 | -0.090 | -0.065 | 0.000 |
| Ns(age)2 | 2 | -0.063 | -0.076 | -0.050 | 0.000 |
| Ns(age)3 | 2 | 0.048 | 0.038 | 0.059 | 0.000 |
| Ns(diabetes\_duration)1 | 2 | -0.025 | -0.033 | -0.017 | 0.000 |
| Ns(diabetes\_duration)2 | 2 | -0.044 | -0.052 | -0.037 | 0.000 |
| Ns(diabetes\_duration)3 | 2 | -0.026 | -0.033 | -0.019 | 0.000 |
| macrovasc\_compTRUE | 2 | -0.370 | -0.375 | -0.365 | 0.000 |
| dkdTRUE | 2 | -0.181 | -0.189 | -0.173 | 0.000 |
| employment\_statusRetired | 2 | 0.017 | 0.006 | 0.027 | 0.002 |
| employment\_statusUnemployed | 2 | 0.009 | 0.000 | 0.017 | 0.052 |
| Ns(household\_income)1 | 2 | -0.010 | -0.019 | 0.000 | 0.040 |
| Ns(household\_income)2 | 2 | -0.013 | -0.021 | -0.005 | 0.001 |
| Ns(household\_income)3 | 2 | -0.005 | -0.012 | 0.002 | 0.156 |
| duration\_of\_residence[10,15) | 2 | 0.033 | -0.023 | 0.090 | 0.242 |
| duration\_of\_residence[15,20) | 2 | 0.027 | -0.018 | 0.071 | 0.238 |
| duration\_of\_residence[20,Inf) | 2 | 0.050 | 0.011 | 0.089 | 0.011 |
| regionCentral Denmark Region | 2 | -0.051 | -0.058 | -0.044 | 0.000 |
| regionNorth Denmark Region | 2 | -0.038 | -0.047 | -0.029 | 0.000 |
| regionSouth Denmark Region | 2 | -0.050 | -0.057 | -0.044 | 0.000 |
| regionZealand Region | 2 | 0.019 | 0.012 | 0.026 | 0.000 |

### Pharmacological treatment: Analysis of ACEI/ARB

| Pharmacological treatment: Analysis of ACEI/ARB | Model | Estimate | Lower 95% CI | Upper 95% CI | P-value |
| --- | --- | --- | --- | --- | --- |
| (Intercept) | 0 | 0.284 | 0.281 | 0.288 | 0.000 |
| originMiddle East | 0 | 0.071 | 0.052 | 0.091 | 0.000 |
| originEurope | 0 | 0.006 | -0.015 | 0.026 | 0.581 |
| originTurkey | 0 | 0.069 | 0.043 | 0.094 | 0.000 |
| originFormer Yugoslavia | 0 | 0.020 | -0.007 | 0.046 | 0.151 |
| originPakistan | 0 | 0.084 | 0.052 | 0.116 | 0.000 |
| originSri Lanka | 0 | 0.080 | 0.035 | 0.125 | 0.001 |
| originSomalia | 0 | 0.143 | 0.063 | 0.224 | 0.000 |
| originVietnam | 0 | -0.037 | -0.097 | 0.023 | 0.230 |

| Pharmacological treatment: Analysis of ACEI/ARB | Model | Estimate | Lower 95% CI | Upper 95% CI | P-value |
| --- | --- | --- | --- | --- | --- |
| (Intercept) | 1 | 0.246 | 0.230 | 0.262 | 0.000 |
| originMiddle East | 1 | 0.081 | 0.061 | 0.100 | 0.000 |
| originEurope | 1 | 0.002 | -0.018 | 0.023 | 0.839 |
| originTurkey | 1 | 0.070 | 0.045 | 0.095 | 0.000 |
| originFormer Yugoslavia | 1 | 0.022 | -0.004 | 0.048 | 0.100 |
| originPakistan | 1 | 0.109 | 0.078 | 0.141 | 0.000 |
| originSri Lanka | 1 | 0.100 | 0.057 | 0.143 | 0.000 |
| originSomalia | 1 | 0.140 | 0.061 | 0.218 | 0.000 |
| originVietnam | 1 | -0.025 | -0.084 | 0.034 | 0.410 |
| sexM | 1 | -0.054 | -0.061 | -0.047 | 0.000 |
| Ns(age)1 | 1 | -0.058 | -0.069 | -0.048 | 0.000 |
| Ns(age)2 | 1 | -0.081 | -0.093 | -0.070 | 0.000 |
| Ns(age)3 | 1 | 0.026 | 0.017 | 0.034 | 0.000 |
| Ns(diabetes\_duration)1 | 1 | -0.039 | -0.050 | -0.028 | 0.000 |
| Ns(diabetes\_duration)2 | 1 | -0.103 | -0.115 | -0.092 | 0.000 |
| Ns(diabetes\_duration)3 | 1 | -0.048 | -0.057 | -0.039 | 0.000 |
| macrovasc\_compTRUE | 1 | 0.036 | 0.022 | 0.049 | 0.000 |
| dkdTRUE | 1 | -0.090 | -0.102 | -0.079 | 0.000 |

| Pharmacological treatment: Analysis of ACEI/ARB | Model | Estimate | Lower 95% CI | Upper 95% CI | P-value |
| --- | --- | --- | --- | --- | --- |
| (Intercept) | 2 | 0.226 | 0.162 | 0.291 | 0.000 |
| originMiddle East | 2 | 0.057 | 0.035 | 0.078 | 0.000 |
| originEurope | 2 | -0.002 | -0.023 | 0.019 | 0.825 |
| originTurkey | 2 | 0.048 | 0.022 | 0.074 | 0.000 |
| originFormer Yugoslavia | 2 | 0.001 | -0.026 | 0.028 | 0.943 |
| originPakistan | 2 | 0.086 | 0.054 | 0.119 | 0.000 |
| originSri Lanka | 2 | 0.092 | 0.049 | 0.135 | 0.000 |
| originSomalia | 2 | 0.112 | 0.033 | 0.192 | 0.005 |
| originVietnam | 2 | -0.032 | -0.091 | 0.027 | 0.284 |
| sexM | 2 | -0.049 | -0.056 | -0.042 | 0.000 |
| Ns(age)1 | 2 | -0.053 | -0.069 | -0.037 | 0.000 |
| Ns(age)2 | 2 | -0.074 | -0.092 | -0.056 | 0.000 |
| Ns(age)3 | 2 | 0.029 | 0.015 | 0.043 | 0.000 |
| Ns(diabetes\_duration)1 | 2 | -0.042 | -0.053 | -0.031 | 0.000 |
| Ns(diabetes\_duration)2 | 2 | -0.107 | -0.118 | -0.095 | 0.000 |
| Ns(diabetes\_duration)3 | 2 | -0.051 | -0.060 | -0.042 | 0.000 |
| macrovasc\_compTRUE | 2 | 0.032 | 0.018 | 0.046 | 0.000 |
| dkdTRUE | 2 | -0.092 | -0.104 | -0.081 | 0.000 |
| employment\_statusRetired | 2 | 0.019 | 0.005 | 0.032 | 0.007 |
| employment\_statusUnemployed | 2 | 0.054 | 0.041 | 0.067 | 0.000 |
| Ns(household\_income)1 | 2 | -0.011 | -0.023 | 0.001 | 0.076 |
| Ns(household\_income)2 | 2 | -0.012 | -0.022 | -0.001 | 0.029 |
| Ns(household\_income)3 | 2 | -0.019 | -0.028 | -0.009 | 0.000 |
| duration\_of\_residence[10,15) | 2 | 0.077 | -0.009 | 0.163 | 0.079 |
| duration\_of\_residence[15,20) | 2 | 0.025 | -0.044 | 0.093 | 0.483 |
| duration\_of\_residence[20,Inf) | 2 | 0.028 | -0.032 | 0.089 | 0.363 |
| regionCentral Denmark Region | 2 | -0.027 | -0.036 | -0.018 | 0.000 |
| regionNorth Denmark Region | 2 | -0.052 | -0.063 | -0.041 | 0.000 |
| regionSouth Denmark Region | 2 | -0.021 | -0.030 | -0.011 | 0.000 |
| regionZealand Region | 2 | -0.016 | -0.026 | -0.006 | 0.001 |

### Pharmacological treatment: Analysis of antiplatelet therapy

| Pharmacological treatment: Analysis of antiplatelet therapy | Model | Estimate | Lower 95% CI | Upper 95% CI | P-value |
| --- | --- | --- | --- | --- | --- |
| (Intercept) | 0 | 0.348 | 0.345 | 0.352 | 0.000 |
| originMiddle East | 0 | 0.012 | -0.008 | 0.031 | 0.240 |
| originEurope | 0 | 0.011 | -0.011 | 0.033 | 0.336 |
| originTurkey | 0 | -0.016 | -0.041 | 0.010 | 0.222 |
| originFormer Yugoslavia | 0 | 0.027 | -0.001 | 0.055 | 0.062 |
| originPakistan | 0 | -0.062 | -0.092 | -0.032 | 0.000 |
| originSri Lanka | 0 | -0.018 | -0.062 | 0.026 | 0.428 |
| originSomalia | 0 | 0.183 | 0.102 | 0.264 | 0.000 |
| originVietnam | 0 | 0.041 | -0.027 | 0.109 | 0.240 |

| Pharmacological treatment: Analysis of antiplatelet therapy | Model | Estimate | Lower 95% CI | Upper 95% CI | P-value |
| --- | --- | --- | --- | --- | --- |
| (Intercept) | 1 | 0.647 | 0.628 | 0.665 | 0.000 |
| originMiddle East | 1 | 0.015 | -0.003 | 0.034 | 0.110 |
| originEurope | 1 | 0.017 | -0.004 | 0.038 | 0.112 |
| originTurkey | 1 | -0.018 | -0.043 | 0.007 | 0.155 |
| originFormer Yugoslavia | 1 | 0.027 | 0.000 | 0.054 | 0.051 |
| originPakistan | 1 | -0.034 | -0.063 | -0.005 | 0.021 |
| originSri Lanka | 1 | -0.025 | -0.066 | 0.016 | 0.230 |
| originSomalia | 1 | 0.133 | 0.056 | 0.211 | 0.001 |
| originVietnam | 1 | -0.015 | -0.077 | 0.046 | 0.623 |
| sexM | 1 | -0.053 | -0.060 | -0.046 | 0.000 |
| Ns(age)1 | 1 | -0.002 | -0.013 | 0.009 | 0.666 |
| Ns(age)2 | 1 | -0.068 | -0.079 | -0.057 | 0.000 |
| Ns(age)3 | 1 | 0.052 | 0.043 | 0.061 | 0.000 |
| Ns(diabetes\_duration)1 | 1 | -0.019 | -0.031 | -0.008 | 0.001 |
| Ns(diabetes\_duration)2 | 1 | -0.055 | -0.066 | -0.043 | 0.000 |
| Ns(diabetes\_duration)3 | 1 | -0.037 | -0.046 | -0.028 | 0.000 |
| macrovasc\_compTRUE | 1 | -0.319 | -0.336 | -0.302 | 0.000 |
| dkdTRUE | 1 | 0.026 | 0.013 | 0.040 | 0.000 |

| Pharmacological treatment: Analysis of antiplatelet therapy | Model | Estimate | Lower 95% CI | Upper 95% CI | P-value |
| --- | --- | --- | --- | --- | --- |
| (Intercept) | 2 | 0.585 | 0.521 | 0.649 | 0.000 |
| originMiddle East | 2 | 0.008 | -0.013 | 0.029 | 0.445 |
| originEurope | 2 | 0.015 | -0.006 | 0.037 | 0.170 |
| originTurkey | 2 | -0.026 | -0.052 | -0.001 | 0.046 |
| originFormer Yugoslavia | 2 | 0.022 | -0.005 | 0.050 | 0.112 |
| originPakistan | 2 | -0.051 | -0.080 | -0.021 | 0.001 |
| originSri Lanka | 2 | -0.013 | -0.054 | 0.028 | 0.545 |
| originSomalia | 2 | 0.127 | 0.048 | 0.206 | 0.002 |
| originVietnam | 2 | -0.007 | -0.069 | 0.054 | 0.812 |
| sexM | 2 | -0.053 | -0.060 | -0.046 | 0.000 |
| Ns(age)1 | 2 | -0.001 | -0.018 | 0.017 | 0.954 |
| Ns(age)2 | 2 | -0.069 | -0.087 | -0.051 | 0.000 |
| Ns(age)3 | 2 | 0.055 | 0.041 | 0.070 | 0.000 |
| Ns(diabetes\_duration)1 | 2 | -0.020 | -0.031 | -0.008 | 0.001 |
| Ns(diabetes\_duration)2 | 2 | -0.057 | -0.068 | -0.045 | 0.000 |
| Ns(diabetes\_duration)3 | 2 | -0.038 | -0.047 | -0.029 | 0.000 |
| macrovasc\_compTRUE | 2 | -0.319 | -0.335 | -0.302 | 0.000 |
| dkdTRUE | 2 | 0.027 | 0.013 | 0.040 | 0.000 |
| employment\_statusRetired | 2 | 0.021 | 0.006 | 0.035 | 0.005 |
| employment\_statusUnemployed | 2 | 0.029 | 0.016 | 0.042 | 0.000 |
| Ns(household\_income)1 | 2 | 0.008 | -0.005 | 0.021 | 0.235 |
| Ns(household\_income)2 | 2 | 0.015 | 0.004 | 0.025 | 0.009 |
| Ns(household\_income)3 | 2 | 0.022 | 0.012 | 0.032 | 0.000 |
| duration\_of\_residence[10,15) | 2 | 0.059 | -0.026 | 0.143 | 0.172 |
| duration\_of\_residence[15,20) | 2 | 0.084 | 0.018 | 0.151 | 0.013 |
| duration\_of\_residence[20,Inf) | 2 | 0.060 | 0.001 | 0.118 | 0.046 |
| regionCentral Denmark Region | 2 | -0.045 | -0.055 | -0.036 | 0.000 |
| regionNorth Denmark Region | 2 | -0.030 | -0.042 | -0.018 | 0.000 |
| regionSouth Denmark Region | 2 | -0.038 | -0.048 | -0.029 | 0.000 |
| regionZealand Region | 2 | -0.016 | -0.027 | -0.006 | 0.002 |
