# Supplementary material for: Guideline-level monitoring, biomarker levels and pharmacological treatment in migrants and native Danes with type 2 diabetes: Population-wide analyses
Source: PLOS Glob Public Health. 2023 Oct 18;3(10):e0001277. doi: 10.1371/journal.pgph.0001277 (PMC10584163; doi:10.1371/journal.pgph.0001277)
Supplement: S9 File — (HTML) [file pgph.0001277.s009.html]

S9. Biomarker data coverage in the background population across geographic regions of Denmark.


# S9. Biomarker data coverage in the background population across geographic regions of Denmark.

- S9. Biomarker data coverage in the background population across geographic regions of Denmark.
  - HbA1c (excl. POCT): Proportion of background population screened each quarter 2015 - 2018
  - HbA1c (incl. POCT): Proportion of background population screened each quarter 2015 - 2018
  - LDL-C: Proportion of background population screened each quarter 2015 - 2018
  - UACR: Proportion of background population screened each quarter 2015 - 2018

# S9. Biomarker data coverage in the background population across geographic regions of Denmark.

Proportion of population receiving laboratory testing during each quarter (out of all adults residing in each region at the end of the calendar year).

In the time-frame from Q4 2015 through Q4 2017, the proportions of the background population undergoing biomarker testing appear comparable across all regions for both HbA1c (after accounting for the use of point-of-care testing), LDL-C and UACR testing. Outside this time-frame, a few anomalies in the data were observed:

- South Denmark Region:

  - Before Q4 2015, the proportions in all tests were lower compared to the other regions.
- Capital Region:

  - From Q1 2018 onward, the proportion of individuals undergoing LDL-C testing drops dramatically. Further investigation (not shown) revealed this drop to be limited to specific municipalities in the region, where were almost no records could be found in the data from 2018 onward.

### HbA1c (excl. POCT): Proportion of background population screened each quarter 2015 - 2018

| Quarter of year | Capital Region | Central Denmark Region | North Denmark Region | South Denmark Region | Zealand Region |
| --- | --- | --- | --- | --- | --- |
| 2015\_Q1 | 134,356 (9.2) | 119,076 (11.3) | 61,238 (12.7) | 1,399 (0.1) | 88,291 (13.0) |
| 2015\_Q2 | 131,519 (9.0) | 116,515 (11.1) | 60,461 (12.5) | 1,270 (0.1) | 86,482 (12.7) |
| 2015\_Q3 | 117,941 (8.0) | 104,598 (9.9) | 55,947 (11.6) | 48,943 (4.9) | 80,423 (11.8) |
| 2015\_Q4 | 133,025 (9.1) | 115,894 (11.0) | 62,007 (12.8) | 126,325 (12.7) | 86,421 (12.7) |
| 2016\_Q1 | 133,960 (9.2) | 120,786 (11.5) | 65,187 (13.6) | 132,477 (13.5) | 91,369 (13.5) |
| 2016\_Q2 | 142,761 (9.8) | 125,709 (12.0) | 68,619 (14.3) | 140,058 (14.2) | 94,830 (14.0) |
| 2016\_Q3 | 122,676 (8.4) | 108,118 (10.3) | 59,197 (12.4) | 120,543 (12.2) | 84,201 (12.5) |
| 2016\_Q4 | 138,343 (9.5) | 120,184 (11.5) | 64,858 (13.5) | 132,022 (13.4) | 92,022 (13.6) |
| 2017\_Q1 | 148,244 (10.2) | 131,559 (12.6) | 70,512 (14.9) | 144,524 (14.8) | 100,715 (15.1) |
| 2017\_Q2 | 139,043 (9.5) | 124,581 (12.0) | 66,103 (13.9) | 134,558 (13.8) | 91,306 (13.7) |
| 2017\_Q3 | 132,168 (9.1) | 113,238 (10.9) | 62,241 (13.1) | 125,902 (12.9) | 85,800 (12.8) |
| 2017\_Q4 | 151,777 (10.4) | 124,888 (12.0) | 67,832 (14.3) | 137,658 (14.1) | 93,931 (14.1) |
| 2018\_Q1 | 152,501 (10.7) | 126,723 (12.4) | 67,907 (14.6) | 141,482 (14.8) | 95,234 (14.5) |
| 2018\_Q2 | 159,617 (11.2) | 133,170 (13.0) | 71,466 (15.4) | 147,353 (15.4) | 98,824 (15.0) |
| 2018\_Q3 | 134,613 (9.4) | 112,250 (11.0) | 60,296 (13.0) | 124,099 (13.0) | 85,054 (12.9) |
| 2018\_Q4 | 152,433 (10.7) | 130,094 (12.7) | 65,538 (14.1) | 133,229 (14.0) | 90,356 (13.7) |

### HbA1c (incl. POCT): Proportion of background population screened each quarter 2015 - 2018

| Quarter of year | Capital Region | Central Denmark Region | North Denmark Region | South Denmark Region | Zealand Region |
| --- | --- | --- | --- | --- | --- |
| 2015\_Q1 | 147,946 (10.1) | 119,083 (11.3) | 61,243 (12.7) | 1,410 (0.1) | 88,592 (13.0) |
| 2015\_Q2 | 146,046 (10.0) | 116,519 (11.1) | 60,467 (12.5) | 1,281 (0.1) | 86,754 (12.7) |
| 2015\_Q3 | 131,779 (9.0) | 104,602 (9.9) | 55,949 (11.6) | 48,957 (4.9) | 80,664 (11.9) |
| 2015\_Q4 | 147,722 (10.1) | 115,897 (11.0) | 62,009 (12.8) | 126,327 (12.7) | 86,698 (12.7) |
| 2016\_Q1 | 148,977 (10.2) | 120,796 (11.5) | 65,191 (13.6) | 132,491 (13.5) | 91,698 (13.6) |
| 2016\_Q2 | 158,441 (10.8) | 125,716 (12.0) | 68,620 (14.3) | 140,069 (14.2) | 95,145 (14.1) |
| 2016\_Q3 | 137,311 (9.4) | 108,122 (10.3) | 59,197 (12.4) | 120,550 (12.2) | 84,482 (12.5) |
| 2016\_Q4 | 153,768 (10.5) | 120,186 (11.5) | 64,859 (13.5) | 132,025 (13.4) | 92,292 (13.7) |
| 2017\_Q1 | 164,443 (11.3) | 131,570 (12.6) | 70,520 (14.9) | 144,541 (14.8) | 101,034 (15.1) |
| 2017\_Q2 | 154,938 (10.6) | 124,595 (12.0) | 66,110 (13.9) | 134,573 (13.8) | 91,645 (13.7) |
| 2017\_Q3 | 147,320 (10.1) | 113,246 (10.9) | 62,245 (13.1) | 125,908 (12.9) | 86,067 (12.9) |
| 2017\_Q4 | 167,698 (11.5) | 124,889 (12.0) | 67,832 (14.3) | 137,663 (14.1) | 94,244 (14.1) |
| 2018\_Q1 | 168,380 (11.8) | 126,735 (12.4) | 67,908 (14.6) | 141,493 (14.8) | 95,572 (14.5) |
| 2018\_Q2 | 176,332 (12.3) | 133,176 (13.0) | 71,468 (15.4) | 147,362 (15.4) | 99,146 (15.1) |
| 2018\_Q3 | 149,906 (10.5) | 112,255 (11.0) | 60,300 (13.0) | 124,105 (13.0) | 85,358 (13.0) |
| 2018\_Q4 | 169,403 (11.9) | 130,099 (12.7) | 65,539 (14.1) | 133,233 (14.0) | 90,665 (13.8) |

### LDL-C: Proportion of background population screened each quarter 2015 - 2018

| Quarter of year | Capital Region | Central Denmark Region | North Denmark Region | South Denmark Region | Zealand Region |
| --- | --- | --- | --- | --- | --- |
| 2015\_Q1 | 126,058 (8.6) | 102,476 (9.7) | 54,616 (11.3) | 1,178 (0.1) | 78,392 (11.5) |
| 2015\_Q2 | 121,990 (8.3) | 99,280 (9.4) | 53,532 (11.1) | 1,046 (0.1) | 75,854 (11.1) |
| 2015\_Q3 | 107,060 (7.3) | 86,144 (8.2) | 48,596 (10.0) | 48,276 (4.9) | 68,740 (10.1) |
| 2015\_Q4 | 121,113 (8.3) | 96,917 (9.2) | 52,449 (10.8) | 125,289 (12.6) | 74,510 (10.9) |
| 2016\_Q1 | 123,190 (8.4) | 99,998 (9.6) | 54,895 (11.5) | 129,673 (13.2) | 79,039 (11.7) |
| 2016\_Q2 | 130,919 (8.9) | 104,981 (10.0) | 57,906 (12.1) | 136,259 (13.8) | 81,699 (12.1) |
| 2016\_Q3 | 109,722 (7.5) | 84,485 (8.1) | 48,240 (10.1) | 114,413 (11.6) | 70,143 (10.4) |
| 2016\_Q4 | 125,008 (8.5) | 96,795 (9.2) | 53,724 (11.2) | 126,377 (12.8) | 77,833 (11.5) |
| 2017\_Q1 | 135,575 (9.3) | 106,084 (10.2) | 58,410 (12.3) | 138,210 (14.2) | 85,747 (12.8) |
| 2017\_Q2 | 125,487 (8.6) | 99,220 (9.5) | 53,880 (11.3) | 127,040 (13.0) | 77,874 (11.7) |
| 2017\_Q3 | 116,935 (8.0) | 88,137 (8.5) | 49,388 (10.4) | 117,324 (12.0) | 71,714 (10.7) |
| 2017\_Q4 | 123,652 (8.5) | 100,385 (9.7) | 55,378 (11.7) | 129,324 (13.3) | 78,970 (11.8) |
| 2018\_Q1 | 67,369 (4.7) | 102,505 (10.0) | 55,674 (12.0) | 132,628 (13.9) | 79,931 (12.2) |
| 2018\_Q2 | 70,731 (5.0) | 109,266 (10.7) | 59,370 (12.8) | 138,584 (14.5) | 83,428 (12.7) |
| 2018\_Q3 | 57,525 (4.0) | 88,420 (8.7) | 48,698 (10.5) | 113,971 (11.9) | 69,735 (10.6) |
| 2018\_Q4 | 65,817 (4.6) | 105,411 (10.3) | 56,378 (12.1) | 124,133 (13.0) | 76,293 (11.6) |

### UACR: Proportion of background population screened each quarter 2015 - 2018

| Quarter of year | Capital Region | Central Denmark Region | North Denmark Region | South Denmark Region | Zealand Region |
| --- | --- | --- | --- | --- | --- |
| 2015\_Q1 | 28,503 (1.9) | 29,480 (2.8) | 10,449 (2.2) | 199 (0.0) | 14,984 (2.2) |
| 2015\_Q2 | 27,932 (1.9) | 29,211 (2.8) | 10,357 (2.1) | 189 (0.0) | 14,517 (2.1) |
| 2015\_Q3 | 24,763 (1.7) | 25,157 (2.4) | 8,937 (1.8) | 8,264 (0.8) | 13,408 (2.0) |
| 2015\_Q4 | 28,048 (1.9) | 28,387 (2.7) | 10,560 (2.2) | 22,078 (2.2) | 15,220 (2.2) |
| 2016\_Q1 | 22,323 (1.5) | 30,603 (2.9) | 11,607 (2.4) | 24,935 (2.5) | 16,417 (2.4) |
| 2016\_Q2 | 23,105 (1.6) | 33,344 (3.2) | 12,314 (2.6) | 26,089 (2.7) | 17,309 (2.6) |
| 2016\_Q3 | 18,257 (1.2) | 26,842 (2.6) | 10,102 (2.1) | 23,130 (2.3) | 14,856 (2.2) |
| 2016\_Q4 | 22,142 (1.5) | 33,158 (3.2) | 11,725 (2.4) | 27,074 (2.8) | 17,035 (2.5) |
| 2017\_Q1 | 24,926 (1.7) | 38,010 (3.7) | 13,475 (2.8) | 30,714 (3.2) | 19,492 (2.9) |
| 2017\_Q2 | 23,483 (1.6) | 36,796 (3.5) | 12,290 (2.6) | 28,201 (2.9) | 17,329 (2.6) |
| 2017\_Q3 | 22,340 (1.5) | 32,702 (3.1) | 10,913 (2.3) | 26,198 (2.7) | 16,337 (2.4) |
| 2017\_Q4 | 27,889 (1.9) | 38,669 (3.7) | 12,556 (2.6) | 38,891 (4.0) | 18,913 (2.8) |
| 2018\_Q1 | 28,857 (2.0) | 40,146 (3.9) | 13,965 (3.0) | 40,640 (4.3) | 20,611 (3.1) |
| 2018\_Q2 | 30,944 (2.2) | 44,281 (4.3) | 15,152 (3.3) | 44,499 (4.7) | 21,426 (3.3) |
| 2018\_Q3 | 24,348 (1.7) | 35,347 (3.5) | 12,356 (2.7) | 35,494 (3.7) | 18,251 (2.8) |
| 2018\_Q4 | 28,452 (2.0) | 43,041 (4.2) | 13,988 (3.0) | 38,861 (4.1) | 21,088 (3.2) |
