# Supplementary material for: Guideline-level monitoring, biomarker levels and pharmacological treatment in migrants and native Danes with type 2 diabetes: Population-wide analyses
Source: PLOS Glob Public Health. 2023 Oct 18;3(10):e0001277. doi: 10.1371/journal.pgph.0001277 (PMC10584163; doi:10.1371/journal.pgph.0001277)
Supplement: S10 File — (PDF) [file pgph.0001277.s010.pdf]

S10: Overview of risks in migrants compared to native Danes

Absolute numbers of indicators with differences statistically significant at alpha = 0.05. Indicators of decreased risk shown in green, increased risk shown in red. Majorities within totals highlighted in **bold**

	Monitoring		Biomarker levels		Pharmacological treatment		Totals of migrant groups	
Crude model	(5)		(2)		(4)		(11)	
Middle East	0	5	0	2	0	1	0/11	**8/11**
Europe	0	5	0	1	0	2	0/11	**8/11**
Turkey	0	4	0	2	2	1	2/11	**7/11**
Former Yugoslavia	0	2	0	2	2	0	2/11	4/11
Pakistan	0	4	0	2	2	1	2/11	**7/11**
Sri Lanka	4	1	0	1	2	1	**6/11**	3/11
Somalia	0	5	0	2	0	4	0/11	**11/11**
Vietnam	0	2	0	0	1	0	1/11	2/11
Totals of indicator	4/40	**28/40**	0/16	**12/16**	9/32	10/32	13/88	**50/88**
Model 1								
Middle East	0	3	0	2	0	2	0/11	**7/11**
Europe	0	5	0	2	0	2	0/11	**9/11**
Turkey	1	3	0	2	1	1	2/11	**6/11**
Former Yugoslavia	2	2	0	1	1	1	3/11	4/11
Pakistan	0	4	0	2	1	2	1/11	**8/11**
Sri Lanka	4	1	1	1	1	1	**6/11**	3/11
Somalia	0	3	0	2	0	4	0/11	**9/11**
Vietnam	0	2	1	0	1	0	2/11	2/11
Totals of indicator	7/40	**23/40**	2/16	**12/16**	5/32	13/32	14/88	**48/88**
Model 2								
Middle East	3	1	0	2	0	1	3/11	4/11
Europe	0	5	0	2	0	2	0/11	**9/11**
Turkey	4	1	0	1	2	1	**6/11**	3/11
Former Yugoslavia	2	2	0	1	2	0	4/11	3/11
Pakistan	3	1	0	2	2	2	5/11	5/11
Sri Lanka	4	1	0	1	1	1	5/11	3/11
Somalia	0	2	0	1	0	4	0/11	**7/11**
Vietnam	0	2	2	0	1	0	3/11	2/11
Totals of indicator	16/40	15/40	2/16	**10/16**	8/32	11/32	26/88	36/88
