# Supplementary material for: Guideline-level monitoring, biomarker levels and pharmacological treatment in migrants and native Danes with type 2 diabetes: Population-wide analyses
Source: PLOS Glob Public Health. 2023 Oct 18;3(10):e0001277. doi: 10.1371/journal.pgph.0001277 (PMC10584163; doi:10.1371/journal.pgph.0001277)
Supplement: Attachment — Submitted filename: Response to Reviewers.docx [file pgph.0001277.s011.docx]

Point-by-point response to reviewers

PLOS Global Public Health

PGPH-D-22-01596

“Guideline-level monitoring, biomarker levels and pharmacological treatment in migrants and native Danes with type 2 diabetes: population-wide analyses”

Dear editor and reviewers

Thank you for the positive evaluation of our manuscript and the valuable comments provided. Our point-by-point response is inserted below marked with **bold**. Please refer to the “Revised Manuscript with Track Changes” file for highlighted corrections.

We believe that the suggestions from the reviewers have considerably improved the manuscript - particularly for a global audience unfamiliar with the Danish healthcare system and register data - and we hope that it is now acceptable for publication in *PLOS Global Public Health*.

We thank you very much for your time with the manuscript and look forward to receiving your response.

On behalf of the authors.

Yours sincerely,

Anders Aasted Isaksen, MD

Department of Public Health, Aarhus University

Bartholins Allé 2, DK-8000 Aarhus, Denmark

Email: [aai@ph.au.dk](mailto:aai@ph.au.dk)

**Review Comments to the Author:**

Reviewer #1:

It is a good work. I have only a comment on the abstract section that missed the result part.

The authors should include result part under the abstract.

**Response: We agree with the reviewer that the abstract should contain estimates, although we initially chose to omit them to keep the abstract short. To accommodate this comment, we have added estimates to the abstract, and revised a few sentences for brevity.**

Additionally, I am not comfortable with the text indentation and Alignment. Alignment should be Justified. **Response: The manuscript was written using the PLOS LaTeX template, which includes standards for formatting indentation and alignment of text according to the specifications of the journal. Therefore, we’ve chosen to keep the indentation format and left-alignment of text unchanged.**

Tables should be beneath its label (Table 3).

**Response: All tables, figures and their labels are formatted in accordance with the PLOS LaTeX template.**

Reviewer #2: An important study assessing the disparities in prevalence, monitoring, and implementation of clinical guidelines for type 2 diabetes comparing native Danes and immigrants using register-based/secondary data.

The justification to exclude adults younger than 25 years, second-generation descendants of immigrants or immigrants residing in Denmark for less than 3 years on the index date, and migrants from countries not in the selected origin categories, is not provided. Please clarify. Figure 1 was not legible/clear which means that I wasn’t able to read it.

**Response: The manuscript has been updated to provide a brief justification for exclusions (lines 48-54). A detailed point-by-point explanation is provided below:**

**- Exclusion of adults younger than 25 and older than 99 was done to avoid bias due to potential differences in diabetes management of these individuals (extreme age-outliers in the T2D population) that could lead to misclassification of register-based of diabetes type.**

**- Immigrants residing in Denmark for less than 3 years on the index date were excluded, as their data would either be missing or fail to accurately represent the independent variables (e.g. employment status, family income and prevalent complications) and the monitoring outcomes, which were assessed retrospective to the index date.**

**- Second-generation migrants were excluded to make interpretation of findings clear, as previous studies have shown that this group is distinct from first-generation migrants in terms of diabetes mortality. We refrained from conducting stratified analyses, as it would have been impossible for us to compute accurate estimates for second-generation immigrants with type 2 diabetes, as these were much younger and only constituted a few percent of the overall type 2 diabetes population.**

**- As the text and plots were already complex and content-heavy, we chose to omit the remaining origin countries, rather than including a mixed “other” group, which we assessed would have been too small and too heterogeneous to hold any meaningful information.**

**Unfortunately, fig. 1 appears to have been downscaled in the merged pdf file during the submission process. It is legible in the uploaded high-resolution image file.**

The authors state that they used datasets from various sources, which is commendable. However, information about how the data linking was achieved at the individual level is missing. Was there incomplete data linkage or did the investigators find challenges associated with data linkage such as inaccurate data linkage or missing unique identifiers on these datasets? Besides, I also observe that records were available from 5 different sources available at different times, for instance, data on diabetes-specific services are available from 1990 while biomarkers data were only available from 2015 onward. How you addressed potential biases associated with incomplete data linkage in your context requires clarification.

About the definition of diabetes based on the 4 events (lines 86-94). Consider event number (iv), the purchase of GLD drugs. Was the purchaser always a diabetes patient? Or somebody else [such as a family member/partner/friend] may have purchased the drugs for the patient? There is a need to clarify this assumption in the Danish context to prevent misclassification for diabetes, potentially overestimating the prevalence.

**Response: The manuscript has been updated to provide the reader with background information on the Danish healthcare system and the corresponding register data (lines 71-73). A detailed explanation is provided below:**

**- Data was linked using the unique personal identifier given to all Danish citizens at immigration or birth, which is used in all healthcare contacts in Denmark and enables exact individual-level linkage of all records. Thus, there were no incomplete data linkages.**

**- We do not believe that the differences in duration of availability between the data sources induced biases, as all data sources covered the intervals that were used to define the variables and outcomes that were used in the study, with the exception of diabetes status, where all available data was used. We assessed that the historic duration differences between the sources was unlikely to affect the composition of the diabetes population, as almost all individuals fulfilled multiple types of inclusion criteria, and any potential differences between migrant groups would be minimal and non-differential with regards to the outcomes under study.**

**- In Denmark, prescriptions are written to the unique personal identifier, and purchases are registered to this as well. Thus, the register data is not affected by the physical circumstances around the purchase, such as relatives or caregivers with authorization to pick up medications for the patient.**

ATC abbreviation is not defined at the point of initial mention making the reading difficult.

**Response: ATC abbreviation has been added to the corrected manuscript (line 81)**

While ethical approval is not required for register-based studies in Denmark, are there any implications concerning the use of patient data for this study? What about the privacy and confidentiality of patient information? Surprisingly, there are no ethical considerations for use of patient data for such types of studies in Denmark!

**Response: The manuscript has been updated to reflect that there are – of course – strict approval processes associated with using the Danish register data for research (lines 193-194).**

Regarding the adjusted models, what model selection approach did you use to identify the model that best fits your data? This information should be provided.

Model identification and selection. What necessitated the development of more than 1 adjusted model in each sub-analysis? Is there a statistical approach that can be used to find a single parsimonious adjusted model that best fits your dataset in each sub-analysis? I find it unnecessary to develop more than one adjusted model when you can develop a single parsimonious model that best fits your data to be compared with the crude model. This applies to figures 3, 4, and 5.

**Response:**

**The manuscript has been updated to explain the rationale for having two separate adjusted models (lines 170-178). A detailed explanation is provided below:**

**As the aim of the study was to map risks, not suggest causal relationships, we did not attempt to find a single best-fitting model.**

**We chose two separate adjusted models as a means to visualize relative risks in migrants from different perspectives, accounting for differences between migrants and Native Danes.**

**Migrants differ from the native Danish population in terms of biological variables (e.g. sex, age, diabetes duration, prevalent complications) that are well-established, clinically relevant risk factors and may explain differences in care in migrants. Model 1 was chosen to visualize risk disparities, accounting only for these factors that may affect clinicians’ decision-making when planning diabetes care with the individual patient.**

**In addition, migrants differ from native Danes in terms of socio-economic variables (e.g. employment status, income, duration of stay, region of residence), which, while not clinically relevant in a traditional sense, are well-established determinants of health behavior and healthcare service usage. Model 2 was chosen to visualize risk disparities, when also accounting for these factors.**

The discussion is well written but may change depending on how the authors address the comments raised on the methods and results.

**Response:**

**The discussion was updated to more explicitly convey the overall interpretation of the results of each adjusted model (lines 310-314).**
