# Supplementary material for: Guideline-level monitoring, biomarker levels and pharmacological treatment in migrants and native Danes with type 2 diabetes: Population-wide analyses
Source: PLOS Glob Public Health. 2023 Oct 18;3(10):e0001277. doi: 10.1371/journal.pgph.0001277 (PMC10584163; doi:10.1371/journal.pgph.0001277)
Supplement: Attachment — Submitted filename: Response to Reviewers.docx [file pgph.0001277.s012.docx]

Point-by-point response to reviewers

PLOS Global Public Health

PGPH-D-22-01596

“Guideline-level monitoring, biomarker levels and pharmacological treatment in migrants and native Danes with type 2 diabetes: population-wide analyses”

Dear editor and reviewers

Once again, thank you for a positive evaluation of our manuscript and valuable comments. Our point-by-point response is inserted below marked with **bold**. Please refer to the “Revised Manuscript with Track Changes” file for highlighted corrections. After these minor revisions, we hope that it is now acceptable for publication in *PLOS Global Public Health*.

We thank you very much for your time with the manuscript and look forward to receiving your response.

On behalf of the authors.

Yours sincerely,

Anders Aasted Isaksen, MD

Department of Public Health, Aarhus University

Bartholins Allé 2, DK-8000 Aarhus, Denmark

Email: [aai@ph.au.dk](mailto:aai@ph.au.dk)

**Specific reviewer comments:**

*Reviewer #3:*

1. Number of Keywords used in this paper is too many, it needs shortening

**Response: PLOS does not provide a number range for keywords, and as they are not published in the final paper, we see no reason to make changes to the keywords at this stage (they are only used to expedite processing, e.g. informing/attracting potential reviewers).**

2. Abstract is too long ,please adhere to Journal rule and regulation (world count < 300)

**Response: Abstract has been shortened to < 300 words.**

3. Methods : The study design was not clear and who is the provider of medical care for the migrants. Is there any difference between native and migrants in terms of facility they visit and level of professional involved and logistic and supply related issue?, Is migrants have equal access to clinical care as native people?

4. How is the Danish health system function means ,who cover the cost of medical care? is it free or out of pocket payment ?

**Response:**

**3: The abstract and methods section (lines 120-122) have been updated to explicitly state the study design.**

**4: The introduction (lines 21-25) has been updated to describe the healthcare setting and how it relates to migrants in the country.**

5. Discussion: There is discrepancy in terms of monitoring, biochemical level and use of diabetic lower agents and medication use in the two groups but why this happen was not clear and is migrants living in another country also have similar finding. Was their education level and income included in the analysis?

**Response: The introduction (lines 44-48) has been updated to convey the aim of the study more clearly. As the aim of the study was to map out risks, not establish causal associations, we only aimed to explore the observed discrepancies, not explain the ‘why’ behind them. As described under ‘Statistical analysis’ in the materials and methods section, the estimates from ‘Model 2’ are adjusted for equivalised household income. As discussed in the paper, while these estimates are adjusted for clinical and socioeconomic factors, causality should not be assumed.**

*Reviewer #5:*

1. The design of the study was not stated. Please state the study design and this should reflect in the abstract.

**Response: The abstract and methods section (lines 120-122) have been updated to explicitly state the study design.**

2.Line 48: " A study population for the analysis of T2D was defined by excluding ................. The study population must be explicitly defined by the authors before stating the exclusion criteria.

**Response: In this population-wide register-based setting, the study population (containing the individuals which statistical analyses are applied on) was obtained after applying inclusion and exclusion criteria on the source population. Thus, it is not possible to define the study population before applying exclusion criteria.**

3. The sampling approach employed in the retrieval of participants' health records from the data base was not clearly stated. Please briefly describe your method of selection (Sampling method). This is very important to clear doubts about the possibility of the authors using data that had the outcomes they wanted).

**Response: The ‘data sources’ section of the methods and materials section (lines 72-91) has been updated to emphasize the population-wide nature of the study. There was no sampling performed, as register data on the entire adult population of Denmark was retrieved. The data sources cover all outcome-related contacts across the entire Danish healthcare system. Thus, the data should contain all individuals in the country, and all applicable data on the study outcomes.**

4. How did you arrive at the study size? What was the size of the sampling frame?

**Response: Since we had access to data on the entire background population due to the population-wide register-based design of the study, there was no sampling frame. Thus, the study size was determined by the number of adults alive and residing in Denmark on the index date, and the prevalence of T2D among them.**

5. How was the prevalence of T2D assessed or calculated.

**Response: Prevalence of T2D was calculated as the proportion of individuals with T2D (classified as having T2D based on the register data using the described algorithm) in the study population for that analysis, as described in the ‘definition of study populations’ section of ‘methods and materials’ (lines 51-59) and illustrated in figure 1.**

6. Line 65: "information on hospital admission and outpatient contacts from 1994 onward..........." What does onward here mean? Does it mean from 1994 to 2022???? Please state the specific relevant dates or period of data extraction and please make the necessary changes from line 68 -78.

**Response: Manuscript (lines 79-91) has been updated to contain the extraction periods.**

7. I belief the possibility of medical data entry errors may be slim in advanced countries like Denmark, but that possibility cannot be ruled out. Please discuss how you dealt with this issue and its possibility of confounding the outcomes/results.

**Response: The discussion section (lines 449-458) has been updated with more discussion of data quality.**

*Reviewer #6:*

1. Regarding abstract: “In a population-wide, register-based T2D population of 254,097 individuals across nine origin groups (Native Danish, Middle East, Europe, Turkey, Former Yugoslavia, Pakistan, Sri Lanka, Somalia, Vietnam) care was assessed in 11 indicators corresponding to CG recommendations.”

Comment: The statement is difficult to comprehend, could you kindly revise it to improve readability?

**Response: For improved readability, the statement has been revised into two separate sentences: ‘*In a cross-sectional, register-based study on 254,097 individuals with T2D, 11 indicators of guideline-level care were analysed.’*… ‘*Migrants were grouped by countries of origin: Middle East, Europe, Turkey, Former Yugoslavia, Pakistan, Sri Lanka, Somalia, Vietnam.’*.**

2. The justification for conducting the study is missing. Authors must include the justification for conducting the study

**Response: The introduction (lines 44-48) has been updated to convey the justification and aim of the study more clearly.**

3. Line 196: It is not appropriate to commence a statement with numbers. Kindly reword or spell out the number.

**Response: Statement reworded to ‘*Among 3,864,528 individuals age 25 - 99 on 1 January 2018, 262,837 individuals (6.8 %) with T2D were identified.’***

4. Table 1: some of the variables in table 1 are not reported, kindly check.

**Response: To accommodate this comment, the results section (lines 206-223) has been updated to report more variables in the main text. We believe that the new revision strikes a good balance between guiding the reader in the main text without extensive repetition of the table contents.**
