# Supplementary material for: Guideline-level monitoring, biomarker levels and pharmacological treatment in migrants and native Danes with type 2 diabetes: Population-wide analyses
Source: PLOS Glob Public Health. 2023 Oct 18;3(10):e0001277. doi: 10.1371/journal.pgph.0001277 (PMC10584163; doi:10.1371/journal.pgph.0001277)
Supplement: Attachment — Submitted filename: Response to Reviewers_rev3.docx [file pgph.0001277.s013.docx]

Point-by-point response to reviewers

PLOS Global Public Health

PGPH-D-22-01596

“Guideline-level monitoring, biomarker levels and pharmacological treatment in migrants and native Danes with type 2 diabetes: population-wide analyses”

Dear editor and reviewers

Once again, thank you for a positive evaluation of our manuscript and valuable comments. We note that the paper has now been thoroughly evaluated in three rounds of review by *eight* independent reviewers. In order to accommodate some of the comments and changes requested by reviewers in this revision, we had to roll back changes requested by previous reviewers. Therefore we hope, with the current version, that you will be able to reach a decision on whether our results are suitable for publication.

Our point-by-point response is inserted below marked with **bold**. Please refer to the “Revised Manuscript with Track Changes” file for highlighted corrections. After these revisions, we hope that it is now acceptable for publication in *PLOS Global Public Health*.

We thank you very much for your time with the manuscript and look forward to receiving your response.

On behalf of the authors.

Yours sincerely,

Anders Aasted Isaksen, MD

Department of Public Health, Aarhus University

Bartholins Allé 2, DK-8000 Aarhus, Denmark

Email: [aai@ph.au.dk](mailto:aai@ph.au.dk)

**Specific reviewer comments:**

*Reviewer #7:*

1. Page 4 line 130 – is there a reason the start date for retinopathy screening is different than for podiatrist screening? Maybe a typo?

**Response: The difference between the two outcome definitions reflects the differences between the corresponding time-frames for timely monitoring recommended in national treatment guidelines monitoring. This difference is mentioned briefly in the introduction (lines 30-34), but in order to make the methodological differences more clear to the reader, the methods section has been revised slightly (lines 113-117):**

**“Diabetic retinopathy screening was identified by records of retinal photoscreening for diabetic retinopathy at hospital departments (in NPR) or diabetes-specific examinations at ophthalmologist practice between 1 October 2015 and 31 December 2017 (to match the two-year window recommended for retinopathy screening in Danish guidelines).”**

2. General – not sure if it is straightforward but it would be better to use HbA1c % throughout the document as that is the more familiar measure to most readers

**Response: Since 2007, the International Federation of Clinical Chemistry and Laboratory Medicine has recommended that HbA1c concentration be reported in mmol/mol worldwide. To make the paper accessible to readers more familiar with legacy units, the corresponding HbA1c concentration in % has been added in parentheses to any HbA1c levels in the main text, e.g. (line 120):**

**“The outcome measure for HbA1c was a level ≥53 mmol/mol (7.0%)”**

3. I am not particularly fond of page 5 line 155 – Danes as the maximally acculturated group. This study is not about integration or any related concepts to that but about diabetes care and its outcomes. You can take this statement out entirely. It adds no information and is potentially exclusionary towards migrants.

**Response: The description has been revised (lines 139-141):**

**“Duration of residence was calculated from the date of first immigration to Denmark, and categorized as 10 years, 10 - 15 years, 15 - 20 years, and ≥ 20 years, with native Danes categorized in the latter group.”**

4. Line 160 again the inclusion criteria dates for DKD do not line up with the other dates? Is there a reason for this?

**Response: The lower boundary of time-window for assessing DKD was capped at October 2015 for two reasons:**

**a) Data on albuminuria was only partially complete in some regions prior to this date (as mentioned briefly on lines 89-91 of the data sources section). Inter-regional bias might be induced by using albuminuria data prior to this.**

**b) The presence of albuminuria may be caused by acute conditions rather than chronic DKD. Lower-capping the time-window serves to limit the potential for false-positive cases of DKD caused by old records of albuminuria sampled during acute conditions.**

5. Lines 209-213 – I find it hard to follow the way these data are presented. Perhaps refer to the table earlier and do not include all the comparisons?

**Response: Previous reviewers requested more information in the main text, which were accommodated in the previous revision. The section has been rearranged in the updated manuscript, including an early reference to table 1, and some of the previously added text has been rolled back to strike a balance between readability and volume of information in the main text (lines 192-201):**

**“In this T2D study population, native Danes and migrants from Europe shared similar background characteristics, which differed from the remaining migrant groups in most variables. Characteristics of migrant groups in the study population for T2D care are shown in Table 1 (see S2 for characteristics of the study population for analysis of T2D prevalence). The difference was largest in terms of age and household income, as migrants from these groups were younger (median age in these groups ranged between 52 [IQR 47-59] years in the Somalia-group to 63 [55-70] years in the Former Yugoslavia-group vs. 69 [60-76] years in native Danes) and poorer (median household income percentile ranged between 12 [8-19] in the Somalia-group to 31 [17-49] in the Sri Lanka-group vs. 40 [25-66] in native Danes).”**

6. Line 224 – it is the odds of prevalent T2D that are higher, not the risk. This is not a longitudinal study. Indeed, I am a bit confused with regards to the analysis? Is this not a cross-sectional study? Shouldn’t all of these be ORs?

**Response: It is a cross-sectional study, and the metrics are risks and relative risks. Logistic regression/odds ratios would not provide interpretable estimates due to the high prevalence of most outcomes in the study, hence the need for risk/relative risk. While binomial regression is the most natural way to compute relative risk, it is prone to convergence failure. We used robust Poisson regression to avoid this inconvenience, as described in the methods section on statistical analysis (lines 151-153).**

7. Table 1 – Suggest you put cohort descriptions first (N, Sex, Age, Employment, Household income, duration of residence, region etc, then measures of diabetes, then monitoring) and maybe create some separation to make the table more readable. Also, report only % with (95%CI) in the cells and put the total N at the top. You do not need to put an n for each cell.

**Response: The current order of variables follows the variables included in the adjusted models: clinical variables, socioeconomic variables, followed by the distribution of outcomes in the lowest half. We chose not to report confidence intervals or p-values in the table, as the purpose of the table is to show distributions, not to conduct hypothesis-testing.**

**In the original manuscript, the table’s vertical separation followed the PLOS LaTeX template. In the revised manuscript, the vertical separation has been increased, greatly improving readability.**

8. Line 231, remove comma after T2D

**Response: Comma removed in the revised manuscript.**

9. Line 246 – Risk estimates were stable in the Europe – group : I don’t understand what this means. Stable? We do not have time trends here. Again. I think this should all be ORs?!

**Response: Wording has been expanded to specify the context: stability across models and adjustments, not over time (lines 231-233):**

**“Risk estimates were stable across models in the Europe-group, but attenuated with increasing adjustment in other migrant groups.”**

10. In general – I would avoid reporting crude estimates so much in the discussion. The important differences will emerge after controlling for confounders and it is these that need to be highlighted. The real effects are from social determinants of health and not from country of birth. If you do not make this more explicit the paper runs the risk of sounding discriminatory which I am sure is not your intention.

**Response: We agree with the reviewer that if the paper implied causation, it would run the risk of implicitly suggesting that migrants themselves were somehow causing/at fault of any disparities. In the revised manuscript, the discussion has been expanded to make it clear that the paper studies observed risks and does not imply causation. In addition, the paragraph now also explicitly discusses the important effects of adjustment while guiding the reader to consider how the crude estimates may be more relevant than the adjusted estimates, since the distribution of covariates between migrants and native Danes differs dramatically (lines 465-477):**

**”Migrant origin, socioeconomic position, lifestyle and healthcare utilization are intrinsically linked beyond the dimensions covered in our data. As the focus of this study was on risk rather than causality, we did not adjust for possible differences in GP-behavior. As this study maps the risks of sub-optimal care quality in migrant groups, the reported disparities cannot be interpreted as causal effects. The observed disparities between migrants and native Danes diminished considerably after adjustment for differences in clinical and socioeconomic characteristics, and could to some extent be attributed to a much higher prevalence of risk factors of poor T2D care in migrants; particularly young age, low household income and residence in the Capital Region. However, the adjusted risks in migrants should be interpreted with caution due to the extremely skewed distributions of risk factors between migrants and native Danes. Ultimately, the three models provide distinct perspectives on the risks in migrants and highlight the influence of the clinical and socioeconomic contexts in these risks.”**

**While it may be possible to break down the effect of migrant origin into smaller parts (e.g. language barriers, genetic predisposition to T2D at younger age, socioeconomic position, lifestyle), this was not possible in the data available, which was insufficient for causal inference analyses. In addition, even if the aim of the study was to establish causal effects on care quality in migrants, the adjustment strategy would not be straight-forward. E.g. in a causal framework, some covariates, such as having T2D and being young/poor could be considered intermediary variables on the causal pathway from being a migrant to receiving poor T2D care, in which case adjusting for these variables would lead to underestimation of the true effect.**

11. In general – suggest using Sri Lanka-origin, Somalia-origin throughout rather than “group”

**Response: We previously considered using “-origin”, but chose the suffix “-group” to acknowledge that these origin groups were categorized based on the available data, which may not reflect the individuals’ perceived origin (e.g. Kurdish or Tamil minorities immigrating from Turkey and Sri Lanka). Also, the “origin”-suffix alone was syntactically insufficient in some sentences, whereas the “-group”-suffix provided a consisted wording.**

Discussion

12. Could you discuss the implications of ethnicity-specific guidelines. For example, Southeast Asian populations like those in Sri Lanka and Pakistan are more likely to have a lean body mass type risk fo type 2 diabetes where they develop the disease earlier and at lower BMIs than a European population. Could this have an effect on how risk and prevention is handled in these populations if Danish physicians are not screening accordingly?

**Response: The Danish guidelines recognize migrant origin as a genetic risk factor for developing T2D and recommends that physicians particularly consider screening migrants for T2D depending on other risk factors.**

**The revised introduction has been updated to incorporate this context (lines 43-44):**

**“No migrant-specific recommendations are provided for these areas of care, but CG advise taking ethnicity into account as a risk factor when screening for T2D.”**

13. The discussion is largely repeating the findings. Could you perhaps provide some context? Do the Somali-origin migrants experience marginalization, is there a reason for their poor monitoring?

**Response: While we can only speculate on the exact causal reasons behind the observed disparities, the data suggests that migrants from Somalia are particularly marginalized in terms of employment status and household income, as presented in table 1. The discussion on this has been expanded in the revised manuscript to provide more context (lines 328-345):**

**“While HbA1c, LDL-C and UACR can be sampled at the GP, screening for diabetic retinopathy and diabetic foot disease is performed by an ophthalmologist or a podiatrist at an external location, and migrants may be particularly vulnerable to these services not being integrated locally. However, the proportion of the T2D population without timely monitoring varied substantially between the types of monitoring performed at the GP, suggesting that this distinction may be of less importance. Socioeconomic factors such as household income and region of residence were risk factors in all types of monitoring, and appear to contribute substantially to the observed disparities in migrants, despite diabetes monitoring being provided free of charge in all regions of Denmark. Screening for diabetic foot disease is the only service where patient expenditures are not fully covered by the public health insurance, which may explain why it was the indicator with the highest proportion of individuals without timely monitoring. Furthermore, the large disparity between migrants and native Danes in this indicator, which persisted after adjusting for socioeconomic variables, suggests that economic barriers may disproportionately limit access to care in migrant groups. However, the disparities observed in the freely offered monitoring services suggest that the monitoring of diabetes is affected by socioeconomic status beyond the patient's direct expenses.”**

14. Lines 326-330 are not clear.

**Response: This paragraph of the discussion has been reworded for brevity and clarity (lines 310-313):**

**“In the majority of indicators, the attenuation from crude to adjusted estimates was mainly due to adjustment for young age, low income, and residence in the Capital Region of Denmark, as these variables were risk factors of non-fulfillment in nearly all indicators and were much more common among migrants than native Danes.”**

15. General- should not all the estimates be age-standardized?

**Response: In order to account for differences in characteristics beyond age, we chose multiple regression rather than age-standardization as our strategy for adjustment.**

16. Figure 2 – I don’t understand how the colors relate to the figure. The plot is just a crude prevalence estimate? It should be a bar chart then. The model outcomes should be presented in a table.

**Response: Rather than crude prevalence, the plot depicts a binomial model of T2D prevalence as a function of age using restricted cubic splines, described in the main text (lines 153… 158-159): “In the analysis of T2D prevalence… age-specific T2D prevalence in each migrant group was modeled using a binomial model with log-link and restricted cubic splines as a function of age.”**

**The colors of the origin groups and estimates double as legends for the lines in the plot. The paper consistently maps each origin group to the same color throughout all figures.**

**While the estimates could have been presented in a separate table, we assessed that this would add unnecessary length to the paper and distract from its main focus: care quality in T2D.**

17. Figure 3 – Almost all effects much lower after controlling for your confounders. I think this is a really important message. It would also be interesting to consider migrants as a whole rather than country origin groups.

**Response: We agree that the role of adjustment is an important message, and address this in the paper’s conclusion (lines 501-504):**

**“To some extent, the disparities between migrants and native Danes could be attributed to a much higher prevalence of risk factors of poor T2D care in migrants; specifically young age, low household income and residence in the Capital Region.”**

**We believe it would be redundant to consider migrants as one group, as we know there is great heterogeneity in cultural-, social-, and physiological characteristics. Therefore, to gain the most from the available data, we chose to examine disparities between distinct groups of migrants identified by country of origin, as this captures the great heterogeneity within the overall group.**

18.Figure 4 does not add any information.

**Response: We consider the information on HbA1c and LDL-cholesterol levels presented in figure 4 to be important, as these biomarkers are critical risk factors for cardiovascular complications and often guide decision-making in clinical practice.**

*Reviewer #8:*

1. The manuscript presents the finding of a register-based study that examines the disparities in T2D care among migrants compared to native Danes in Denmark. The study analyzed 11 indicators of guideline-level care among a large sample including 254,097 individuals with T2D. The migrants were grouped by their countries of origin, and the results showed that in most migrant groups, T2D was more prevalent than in native Danes. The study found that non-fulfillment was common in eight indicators, with monitoring and biomarker control being inferior among migrants compared to native Danes. Migrants from Somalia received the poorest care overall, with elevated risks in all eleven indicators and exceedingly high lipid levels. The study concludes that T2D care is inferior among most migrant groups in Denmark, and there are significant disparities in care, with migrants from Somalia receiving the poorest care. The authors have adequately addressed the reviewer's concerns.

My comment is only on the reporting the prevalences. The hospital-based prevalence may not resemble the population-based prevalence. Are there any differences in the proportion of referred patients for the diagnosis of diabetes between native Danes and migrants? The possible reasons of such disparity such as structural racism, genetic predisposition, and lifestyle are the future area of interest.

**Response: As described in the methods section, T2D was defined using data from primary care (HbA1c samples, medication purchases, podiatrist services) in addition to hospital diagnoses. The revised manuscript has been reworked for brevity and readability, including a reference to the high validity of this approach (lines 100-104):**

**“T2D was defined using a previously validated classifier, the Open-Source Diabetes Classifier [18], based on the following inclusion events: i) HbA1c measurements of ≥48 mmol/mol (6.5%), ii) hospital diagnoses of diabetes, iii) diabetes-specific podiatrist services, iv) purchases of GLD. Diabetes type was classified as either type 1 or T2D based on patterns of insulin purchases and type-specific hospital diagnoses of diabetes.”**

**The high validity of this approach minimized the risk of disparity in identification of cases of diagnosed diabetes. However, we did not have data regarding who should have been tested (e.g. risk factors for developing T2D), and thus could not directly estimate a potential disparity in (under)diagnosis of T2D among native Danes and migrants.**
